# Kinetically Enhanced Access to a Dynamic Polyester Platform via Sequence Selective Terpolymerisation of Elemental Sulphur

**DOI:** 10.1002/anie.202501337

**Published:** 2025-06-04

**Authors:** Cesare Gallizioli, Peter Deglmann, Alex J. Plajer

**Affiliations:** ^1^ Makromolekulare Chemie, Universität Bayreuth Universitätsstraße 30 95447 Bayreuth Germany; ^2^ BASF SE Carl‐Bosch‐Straße 38 67056 Ludwigshafen am Rhein Germany; ^3^ Bayrisches Polymer Institut (BPI) Universität Bayreuth Universitätsstraße 30 95447 Bayreuth Germany

**Keywords:** Polymerisation catalysis, Ring‐opening (co/ter)polymerisation, Sulphur waste utilisation, Sulphur‐containing polymers

## Abstract

Elemental sulphur, a waste product of the oil refinement process, contains preformed dynamic sulphur–sulphur bonds promising to impart dynamicity onto polymers obtained from this monomer. Yet robust methodologies to access linear polymers with tuneable properties and functionality are rare. Addressing these problems, we here report a rare sequence selective terpolymerisation of elemental sulphur with aromatic thioanhydrides and epoxides under simple lithium alkoxide catalysis yields semi‐aromatic poly(ester‐alt‐S_x_). This enables access to terpolymers from a greatly improved range of epoxide comonomers compared to previous methodologies, including industrially relevant, flexible, rigid, functional and natural product‐derived variants, allowing tuning of glass transition temperatures across a *T*_g_ range of >150 °C. Mechanistic investigations reveal that the insertion of S_8_ leads to an unusual rate acceleration via coordinative participation of the polyester links sitting adjacent to the propagating chain end. The thermal stability of the polymers allows for post polymerisation backbone modification via ─S─S─ bond metathesis. After crosslinking, these can be applied as thermally reprocessable and acid degradable adhesives. Our contribution paves the way for the rational buildup of diverse and functional polymer structures from elemental sulphur waste.

## Graphical abstract

A rare sequence‐selective terpolymerisation of elemental sulphur, aromatic thioanhydrides, and epoxides yields dynamic poly(ester‐alt‐S_x_) polymers. Mechanistic insights reveal a lithium‐catalysed rate enhancement via ester coordination. The resulting polymers are thermally stable, backbone‐editable, and processable into degradable adhesives.

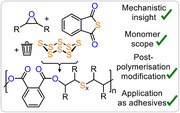

## Introduction

Covalent bonds between sulphur atoms exhibit lower bond dissociation energy than those between atoms of the second period, bonds that current commodity polymers are typically constructed of.^[^ ^1^ ^]^ Hereby, such ─S─S─ bond containing polymers address current sustainability challenges plastics face, as they can exhibit enhanced degradability as well as chemical and mechanical recyclability.^[^ ^2^, ^3^, ^4^ ^]^ Furthermore, the reversible opening and closing of the sulphur–sulphur bond introduces the potential for self‐healing or stimuli responsive behaviour, while the electronic nature of the sulphur centres bestows these materials with unique properties such as the ability for transition metal coordination and increased refractive indices.^[^ ^5^, ^6^, ^7^, ^8^, ^9^, ^10^, ^11^ ^]^ The controlled incorporation of sulphur–sulphur moieties remains, however, challenging, particularly when it comes to linear polymers, and often involves the employment of synthetically non‐trivial building blocks that require elaborate synthesis.^[^ ^12^, ^13^ ^]^

**Figure 1:**
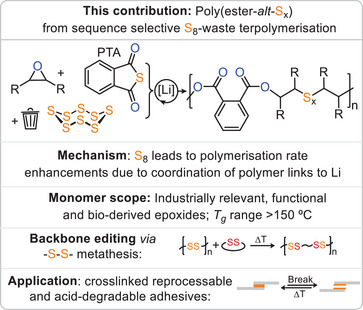
Outline of the current study.

**Figure 2:**
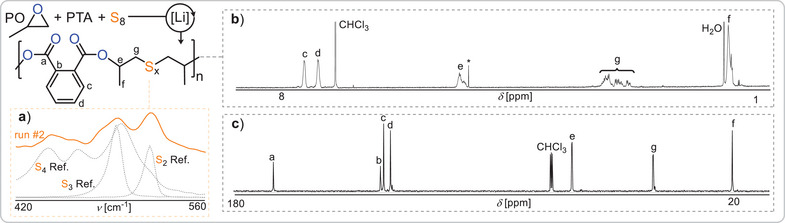
S_8_/PTA/PO ROTERP. a) Raman spectrum of the S_x_ region with S_2_─S_4_ reference spectrum. b) ^1^H NMR and c) ^13^C NMR spectrum (CDCl_3_) corresponding to Table 1 run #3. *Residual CH_2_Cl_2_.

In this regard, elemental sulphur (S_8_) represents a promising starting material, as the desired ─S─S─ bonds are already preformed. Utilisation is furthermore attractive, as S_8_ constitutes a major scale waste product of the petrochemical industry, which is produced as part of the desulphurisation process of crude oil. As S_8_ cannot be polymerised by itself into thermodynamically stable macromolecules, many recent efforts have focused on developing copolymerisation strategies forming polymers with oligosulphur S_x_ links. Typically, this relies on thermally activated radical pathways enabling copolymerisation with (multifunctional) alkenes via so‐called inverse vulcanisation, giving access to a wide range of crosslinked materials.^[^ ^14^, ^15^, ^16^, ^17^, ^18^, ^19^, ^20^, ^21^, ^22^, ^23^ ^]^ In contrast, the synthesis of linear chains from S_8_ in a controlled fashion is more challenging, in particular regarding chains exceeding the oligomer regime.^[^ ^24^, ^25^, ^26^, ^27^ ^]^ In this context the anionic copolymerisation of sulphur relies on the formation of thiolate intermediates that then achieve S_8_ ring opening. Unfortunately, although this mechanistic necessity enables copolymerisation of expensive sulphurated heterocycles, it excludes many commonly applied oxygenated comonomers, generating alkoxide intermediates upon insertion.^[^ ^28^, ^29^ ^]^ Here epoxides would not only be attractive comonomers due to the industrial relevance of propylene oxide (PO) but also because of the commercial availability of many others that potentially allows for a facile tuning of the material properties.^[^ ^30^ ^]^ In this regard we recently found that thiolate intermediates can also be formed during the copolymerisation of CS_2_ with epoxides and thereby enable incorporation of S_8_ and effectively make epoxides compatible monomers for elemental sulphur.^[^ ^31^ ^]^ The ring‐opening terpolymerisation (ROTERP) derives from anionic ring‐opening copolymerisation (ROCOP), in which two monomers are combined in a strictly alternating fashion, and sulphurated variants have recently been in increased focus due to the property and chemical recyclability benefits of sulphur containing polymers.^[^ ^32^, ^33^, ^34^, ^35^, ^36^, ^37^, ^38^, ^39^, ^40^, ^41^, ^42^, ^43^, ^44^, ^45^, ^46^, ^47^, ^48^, ^49^, ^50^, ^51^, ^52^, ^53^ ^]^ In ROTERP, however, three rather than two monomers are terpolymerised in a sequence selective fashion, and the reasons how and when to achieve this recently developed concept are largely unanswered. Unfortunately, though the S_8_ ROTERP depended on an excess of highly toxic carbon disulphide, it exhibited limited epoxide scope and produced thionocarbonate ─O─(C═S)─O─ links, which have poor thermal stability, decomposing into carbonyl sulphide. This hindered processing and hence any applications as well as other controlled utilisation of the ─S─S─ bond. Addressing these problems, we here report the incorporation of elemental sulphur into polyesters (Figure 1) via ROTERP with epoxides and thioanhydrides (Figure 2).

## Results and Discussion

Investigating whether phthalic thioanhydride (PTA) can compatibilise epoxides with elemental sulphur (Figure 1), we attempted ROTERP employing 1 eq. Lithiumbenzyloxide (LiOBn, generated in situ from Li[N(SiMe_3_)_2_] and BnOH), 500 eq. PO, 50 eq. PTA and 100 eq. S (i.e., 1/8 eq. S_8_) at room temperature for 24 h, resulting in full PTA conversion (Table 1 run #1). ^1^H NMR analysis of the product mixture reveals the formation of broad indicative polymer resonances in 97% selectivity alongside some sharp resonances for small molecule byproducts. The polymer could be easily isolated by precipitation in methanol and centrifugation. Gel permeation chromatography (GPC) shows an apparent weight averaged molecular weight of 22 kg mol^−1^ with a polydispersity of 1.5, which confirms the macromolecular nature of the product. NMR analysis reveals the aromatic region of the spectrum to feature two dominant CH resonances at 7.4 and 7.6 ppm, which correlate by HSQC to aryl esters at 166 ppm (see ESI Figure S1), and this is indicative of a symmetrically substituted aryl diester unit. Accordingly, the tertiary CH proton from PO ring‐opening likewise correlates to the quaternary carbon resonance at 166 ppm, implying that these so‐called head‐positions sit adjacent to the phthalate core. The CH_2_ tail position of the ring‐opened PO appear between 3.5 and 2.6 ppm which in comparison to literature known polymers can be tentatively assigned to be connected to oligosulphide ‐S_x_‐ links.^[^ ^29^, ^31^ ^]^ The formation of these is confirmed by Raman spectroscopy showing indicative absorbances between 560 and 420 cm^−1^. Close comparison of these regions to small molecule di‐ RSSR, tri‐ RSSSR and tetra‐ RSSSSR standards indicates that while some disulphide links appear to have formed, a substantial proportion of the ‐S_x_‐ links comprise tri‐ and tetrasulphides (ESI Figure S10). Taken together, the spectroscopic data suggest the selective formation of a poly(ester‐*alt*‐S_x_) sequence in which the ‐S_x_‐ are flanked by CH_2_ tail‐groups while the phthalate esters sit next to CH head groups of the ring opened epoxides. To investigate if the oligosulphide linkage distribution is affected by the amount of elemental sulphur supplied in the starting monomer feed, we halved the amount of supplied sulphur (run #2). Here the reaction rate slowed down substantially, necessitating longer reaction times to reach high monomer conversion with polymer formation in 93% selectivity. Raman spectroscopy now reveals a more prominent formation of disulphide links, suggesting tunability of the ‐S_x_‐ linkage distribution with the supplied initial sulphur feed.

**Table 1:** PTA/S_8_/PO ROTERP.

Run	LiOBn:PO:PTA:⅛S_8_	*T* (°C)	Time	Conversion^a)^ (%)	Polym.^b)^ (%)	Linkage selectivity^c)^ (%)	*M*_w_ ^d)^ (kDa)	*Đ* ^d)^
#1	1:500:50:100	30	2 h	99	93	96	22	1.5
#2	1:500:50:50	30	24 h	87	97	91	18	1.7
#3	1:500:50:200	30	2 h	99	93	97	18	1.5
#4	1:500:50:50	100	5 min	93	99	93	17	1.6
#5	1:500:50:100	100	2 min	99	96	99	11	1.5
#6	1:500:50:200	100	2 min	99	96	98	8	1.3
#7	1:500:200:200	30	72 h	90	99	75	43	1.6
#8	1:500:200:200	100	5 min	85	99	88	35	1.7
#9	1:5000:1000:1000	100	24 h	99	99	97	28	1.6
#10	1:250:25:25	100	2 min	99	99	99	6	1.5
#11	1:250:10:10	100	2 min	99	99	99	6	1.1
#12^e)^	1:500:50:50	30	7 days	70	99	20	4	1.2
#13^f)^	1:500:50:50	30	6 days	70	99	16	6	1.3

^a)^ Relative peak integrals in the normalised ^1^H NMR spectrum (CDCl_3_, 400 MHz) of the crude reaction mixture of aromatic polymer resonances versus unconsumed PTA.

^b)^ Relative peak integrals in the normalised ^1^H NMR spectrum (CDCl_3_, 400 MHz) of the crude reaction mixture of polymer resonances versus small molecule byproducts.

^c)^ Relative peak integrals in the normalised ^1^H NMR spectrum (CDCl_3_, 400 MHz) of polymer resonances corresponding to ester and polysulphide linkages relative to all linkages formed.

^d)^ Determined by GPC measurements conducted in THF with a narrow polystyrene standard.

^e)^ NaOBn was employed.

^f)^ KOBn was employed.

Close inspection of the NMR spectrum reveals minor aryl CH resonances at 7.5 and 7.7 ppm alongside the major set of resonances corresponding to the main poly(ester‐*alt*‐S_x_) sequence. These can be assigned to arylthioesters connected to ring‐opened PO tail positions.^[^ ^54^, ^55^ ^]^ Integration shows that 9% of polymer linkages are thioesters compared to 4% at higher sulphur loading. Formation of thioester links can be ascribed to co‐occuring PTA/PO ROCOP (vide infra). As doubling the sulphur loading (compared to run #1) again decreases thioester errors (run #3), sulphur loading also appears to not only enhance reaction rate but also linkage selectivity. Increasing the reaction temperature (run #4 to #6) to 100 °C results in a substantial improvement of reaction rate so that full conversion is achieved within minutes, albeit somewhat at the expense of molecular weight. Increasing the PTA to LiOBn loading (run #8 and #9) increases the final obtained molecular weights to a maximum of *M_w_ *= 43 kg mol^−1^ at a linkage selectivity of 97%. Conversely, at higher catalyst loadings (run #10 and 11), decreased molecular weights of *M_w_ *= 6 kg mol^−1^ are observed, demonstrating some level of control over the obtained degree of polymerisation with our methodology. From the combined results it appears that sequence selectivity positively correlates to higher S_8_ loading relative to PTA and an increase in reaction temperature. Confirming that lithium acts as a true catalyst, we performed runs with NaOBn and KOBn in place of LiOBn (runs #12 and #13), resulting in slower rates and low elemental sulphur incorporation.

**Figure 3:**
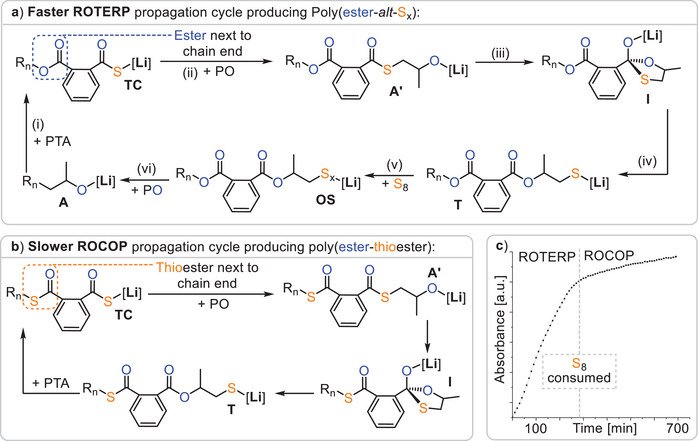
Mechanistic hypothesis for a) S_8_/PTA/PO ROTERP and b) co‐occurring PTA/PO ROCOP. c) Polymerisation performed at 1 eq. LiOBn: 100 eq. S: 200 eq. PTA: 1000 eq. epoxide and 80 °C showing a sudden decrease in polymerisation rate upon elemental sulphur consumption. Note that ethyl glycidyl ether (EGE) was employed in place of PO due to its increased boiling point facilitating analysis by in situ IR spectroscopy.

The structure of the terpolymers let us suggest a propagation mechanism as depicted in Figure 3a. Starting from a lithium alkoxide **A**, such as LiOBn or an intermediate resulting from PO ring opening, propagation occurs in (i) by PTA insertion to form a lithium thiocarboxylate **TC**, which regenerates a lithium alkoxide **A’** via PO insertion in (ii). From here the alkoxide chain end intramolecularly attacks the adjacent thioester group, forming a tetrahedral intermediate **I** that collapses in (iii) and (iv) into a lithium thiolate intermediate **T**. This exchanges the position of an oxygen and sulphur centre in a so‐called O/S exchange reaction. Thereafter S_8_ insertion and equilibration of the S_x_ chain lead to a distribution of lithium oligosulphide intermediates **OS** in (v). Finally, PO insertion leads to (vi), which closes the catalytic cycle and generates the poly(ester‐*alt*‐S_x_) sequence. Alternatively, as shown in Figure 3b, lithium thiolates **T** can insert PTA to close a PTA/PO ROCOP cycle, forming thioester errors. Hence, to achieve high sequence selectivity, thiolates **T** need to selectively insert S_8_ over PTA. Comparing ROTERP and ROCOP, we performed a polymerisation (see Figure 3c) with excess PTA and epoxide compared to S_8_ in the initial monomer feed so that elemental sulphur would deplete over the course of the reaction and monitored the reaction via in situ IR at 1127 cm^−1^ at which both ROTERP and ROCOP could be monitored in separated experiments. Interestingly, we observe a sudden eight fold decrease in reaction rate upon sulphur consumption. This indicates that the polymerisation is accelerated by elemental sulphur, which indeed could be confirmed in separate ROCOP and ROTERP runs under analogous conditions, and this observation is in line with the results of Table 1 (and Table 2, vide infra), in which higher sulphur loading positively correlates with polymerisation rate. Although no clear reaction order in S_8_ could be determined, performing an in situ IR experiment with excess S_8_ and epoxide with respect to PTA reveals an approximately linear correlation of polymer production with time, indicating a 0^th^ order dependence on PTA concentration in line with related terpolymerisations (see ESI Figure S21).^[^ ^56^ ^]^

**Table 2:** Monomer scope of the new methodology.

Run^a)^	Epoxide	LiOBn:Epox.:PTA:⅛S_8_	Time	Conversion^b)^	Polym.^c)^	Linkage select. (%)^d)^	*M*_w_ (kDa)^d)^	*Đ* ^d)^	*T*_g_(°C)^e)^	*T*_d(5%)_(°C)^f)^
#0	PO	1:500:200:200	5 min	85%	99%	88%	35	1.7	30	265
#1	BO	1:500:200:200	1 h	88%	98%	91%	56	1.7	26	276
#2	DO	1:500:200:200	16 h	72%	97%	92%	26	1.5	−23	282
#3	DO	1:500:200:400	16 h	99%	97%	97%	19	1.6	−25	270
#4	CHO	1:500:200:200	16 h	76%	95%	76%	23	1.6	85	270
#5	CHO	1:500:200:400	1h	90%	99%	98%	16	1.6	61	269^k)^
#6	DMO	1:500:200:200	40 h	66%	99%	94%	12	1.5	30	261
#7	IBO^g)^	1:500:200:200	1 h	72%	n.d.	94%	40	1.6	18	190
#8	IBO^g)^	1:500:200:400	1 h	70%	n.d.	96%	27	1.6	13	218
#9	SO^h)^	1:500:200:200	40 h	70%	99%	n.d.	7	1.4	40	250
#10	SO^h)^	1:500:200:400	44 h	86%	99%	n.d.	8	1.6	53	258
#11	PO^i)^	1:500:200:200	2 h	95%	99%	98%	10	1.8	61	279
#12	CHO^i)^	1:500:200:200	65 h	70%	99%	82%	11	1.6	128	270^k)^
#13	EGE	1:500:200:200	10 min	99%	98%	88%	24	1.7	8	274
#14	PGE	1:500:200:200	16 h	99%	95%	96%	25	1.7	47	289
#15	VGE	1:500:200:200	2 h	90%	99%	88%	26	1.7	66	265
#16	VGE^i)^	1:500:200:200	1.5 h	80%	99%	92%	9	1.5	85	273
#17	CE^h)^	1:500:200:200	16 h	66%	99%	n.d.	12	1.6	129	279
#18	ECH	1:500:200:200	5 min	98%	93%	93%	10	2.6	39	256
#19	DEO^j)^	1:500:200:200	1 h	n.d.	n.d.	n.d.	n.d.	n.d.	13	264
#20	ESBO^j)^	1:500:200:200	72 h	n.d.	n.d.	n.d.	n.d.	n.d.	−40	238

^a)^ ROTERP conducted at 100 °C.

^b)^ Relative peak integrals in the normalised ^1^H NMR spectrum (CDCl_3_, 400 MHz) of the crude reaction mixture of aromatic polymer resonances versus unconsumed PTA.

^c)^ Relative peak integrals in the normalised ^1^H NMR spectrum (CDCl_3_, 400 MHz) of the crude reaction mixture of polymer resonances versus small molecule byproducts.

^d)^ Relative peak integrals in the normalised ^1^H NMR spectrum (CDCl_3_, 400 MHz) of polymer resonances corresponding to ester and polysulphide linkages relative to all linkages formed.

^d)^ Determined by GPC measurements conducted in THF with a narrow polystyrene standard.

^e)^ Determined by DSC from the second heating curve at 10 K min^−1^.

^f)^ Determined by thermogravimetric analysis (TGA).

^g),h)^ Complete determination of selectivity not possible due to overlapping signals.

^i)^ NTA was employed in place of PTA.

^j)^ Cross‐linked network is obtained, preventing solution characterisation.

^k)^ minor degradation step observed at lower temperatures.

**Figure 4:**
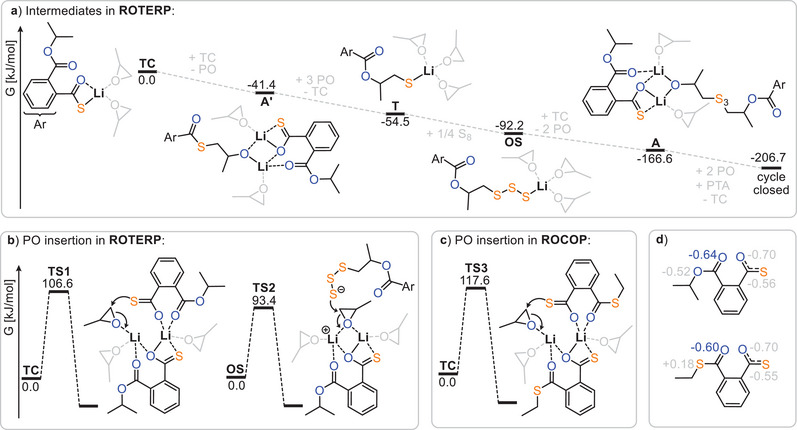
a) Intermediate speciation as well as PO insertion barriers for b) S_8_/PTA/PO ROTERP and c) PTA/PO ROCOP. d) NBO population analysis of thiocaboxylates modelled as monoanionic species produced by (top) ROTERP and (bottom) ROCOP. Isopropyl and ethyl groups at the chain ends were employed for computational simplicity. S_3_ groups were exemplarily employed for oligosulphide groups S_x_. For computational details see ESI Section S6.

To investigate the origin of rate enhancement and sequence selectivity, we turned to density functional theory (DFT, Figure 4) at a meta‐hybrid DFT level.^[^ ^57^, ^58^, ^59^, ^60^, ^61^, ^62^, ^63^, ^64^ ^]^ This involved conformational searches and structure optimisations at the TPSSH/def‐TZVP level (using the solvation model COSMO and assuming a dielectric constant of infinity), followed by M06‐2X/def2‐TZVPD single‐point calculations. For the final Gibbs free energies, the solvation model COSMO‐RS was employed, assuming a solvent mixture of 5:1 PO and PTA at 30 °C. Statistical thermodynamic contributions to Gibbs free energies were also taken into account. The most favoured structures were identified by assessing a multitude of species as exhaustively as possible, while also considering dimerisation equilibria at catalyst concentrations during ROTERP. The reader is referred to the supporting information (ESI Section S6) for a summary of these species and their associated Gibbs free energies.

Figure 4a shows the intermediate speciation and propagation energetics modelled according to the mechanistic hypothesis of Figure 3a, confirming its thermodynamic viability, as Gibbs free energy decreases along any polymer growth step. Based on previous work on related polymerisations, it can be assumed that propagation steps involving the insertion of these monomers, as well as O/S exchange, are faster than the PO insertion steps.^[^ ^31^, ^56^ ^]^ Here, PO ring‐opening by either the lithium thiocarboxylate **TC** (**TS1**, Δ*G* ^‡^ = 106.6 kJ mol^−1^) or the lithium oligosulphide **OS** (**TS2**, Δ*G* ^‡^ = 94.4 kJ mol^−1^, modelled exemplarily for a trisulphide) occurs fastest in a dinuclear fashion, clearly revealing a lithium thiocarboxylate resting state (Figure 4b). While in **TS1** the propagating chain end remains coordinated, **TS2** occurs in a zwitterionic fashion in which the oligosulphide chain end is decoordinated by an equivalent of PO at the dimeric Li_2_ core. We infer that this decoordination is enabled by the high oxophilicity of lithium, which at least partially explains its increased catalytic activity compared to other alkali metals.

Interestingly, the rate‐determining transition state is lowest in energy when the ester group adjacent to the chain end participates in the coordination of the lithium dimer. For PTA/PO ROCOP, the analogous rate‐determining transition state (**TS3** in Figure 4c) likewise involves a dimeric structure. However, in contrast to ROTERP, a thioester group rather than an ester coordinates the lithium, which is associated with a higher insertion barrier of Δ*G* ^‡^ = 117.6 kJ mol^−1^. Thus, we attribute the rate enhancement observed when moving from ROCOP to ROTERP to the presence of an ester, rather than a thioester, adjacent to the chain end. In fact, natural bond orbital (NBO) analysis of thioester and ester appended thiocarboxylates (Figure 4d) reveals the carbonyl oxygen of the latter to feature less accumulation of negative partial charge on the carbonyl oxygen than for the former. This renders esters a better neutral ligand for lithium than thioesters, which we infer to be responsible for the observed rate‐enhancement.

Regarding selectivity, i.e., the seemingly preferential S_8_ over PTA insertion by the lithium thiolate intermediate **T** (Figure 3), we found that PTA insertion is practically as exergonic as S_8_ insertion, with a ΔΔ*G* = 2.6 kJ mol^−1^ in favour of PTA incorporation. However, propagation via PO insertion thereafter is more favoured from oligosulphides **OS** by ΔΔ*G* ^‡^ = 24.2 kJ mol^−1^). This implies that PTA insertion by **T** must be reversible to allow for the correction of erroneous insertions during ROTERP, which we confirmed experimentally (see ESI Figure S17). Here, a model lithium thiolate (^i^BuSLi) was first reacted with PTA, and subsequently elemental sulphur was added. Indeed, ^1^H NMR analysis of the reaction mixture revealed initial consumption of PTA, which reformed after the addition of S_8_.

Importantly the crude reaction mixture also shows the presence of some minor resonances (1%–7% by integration) corresponding to small molecule byproducts which can be easily separated during work‐up (see ESI Figures S3 and S4; Scheme S1). GPC analysis shows these to be primarily species in the small molecule regime >1 kg mol^−1^, as for example, cyclised repeat units (or doubles and triples of these). This suggests the presence of some backbiting reactions as competitive depolymerisation pathways alongside propagation. In these **T** reacts with an oligosulphide link near the chain end within the same polymer chain instead of with S_8_ thereby leading to the formation of macrocycles. This pathway could be tentatively confirmed via exposure of a terpolymer to in situ generated thiolates that, upon thermal activation, lead to some depolymerisation as seen by ^1^H NMR. Furthermore, obtained weights are lower than theoretical molecular weights. We previously observed in terpolymerisations of S_8_ that sulphur–sulphur polymer linkages act as chain‐transfer sites, decreasing molecular weights and broadening polydispersities.^[^ ^31^ ^]^ Here thiolate **T** attacks more oligosulphide links or links of other polymers effectively catalysing sulphur–sulphur bond metathesis which can lead to the formation of multiple cyclic polymers instead of a single polymer chain and thereby reduce the obtained molecular weight. In fact, following the CH_2_ region of the crude ^1^H NMR spectrum reveals that longer S_x_ links decrease in proportion in favour of shorter S_x_ links as elemental sulphur is consumed, confirming that S_x_ undergoes metathesis during ROTERP. Furthermore, such a methathesis can also occur spontaneously, as very recently demonstrated.^[^ ^65^ ^]^ In fact, prolonged reaction of a terpolymer with dimethyltrisulphide MeS_3_Me in PO at 100 °C leads to a reduction of molecular weight by cleaving polymer chains and end‐capping these with a Me group (see ESI Figure, Scheme S1 and Figure S94). This suggests that sulphur–sulphur bond metathesis can also occur spontaneously under reaction conditions.

**Figure 5:**
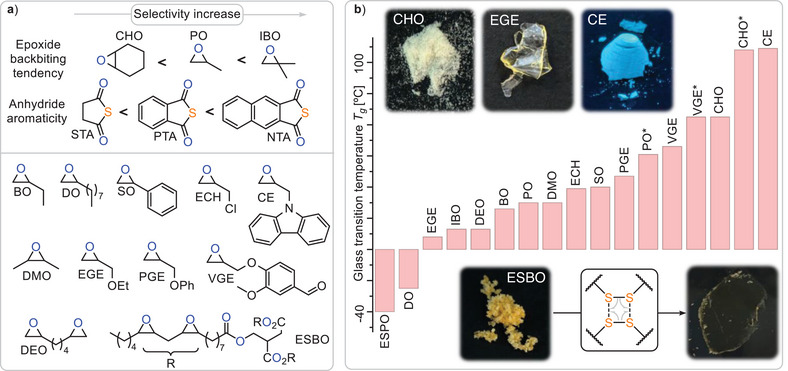
a) Monomer scope of the methodology elucidating selectivity governing factors. b) *T*_g_ range and optical photographs of obtained polymers for Table 2 run at 1 eq. LiOBn: 500 eq. epoxide: 200 eq. thioanhydride: 200 eq. S.

Next, we turned to exploring the monomer scope of our methodology (Figure 5). We decided to survey our methodology at an initial loading of 1 eq. LiOBn: 500 eq. epoxide: 200 eq. PTA: 200 eg. S at 100 °C which for PO (Table 2 run #0) delivers a linkage selectivity of 87%, hence allowing us to study how monomer choice affects selectivity, shedding further mechanistic light on our methodology.

Moving from PO to its longer chain variants BO (run #1) and DO (run #2) results in a slight improvement of linkage selectivity, while as expected lowering the glass transition temperature from 30 °C for the PO terpolymer to −23 °C for the DO terpolymer. As for PO, the addition of more sulphur to the initial monomer feed ratio (run #3) results in an increase to 97% selectivity. Moving to bicyclic CHO (run #4) results in 76% linkage selectivity and an increased *T*_g_ of 85 °C, and both can be attributed to the rigidity of the cyclohexyl backbone. Mechanistically this leads to a strained bicyclic intermediate upon backbiting, thereby disfavouring O/S exchange and S_8_ incorporation.^[^ ^66^ ^]^ Nevertheless, an increase in sulphur loading achieves near perfect linkage selectivity (run #5), which has been challenging to achieve in related sulphurated polymerisations.^[^ ^67^, ^68^ ^]^ In line with this interpretation, the geminally disubstituted epoxide IBO (run #7), that favours backbiting due to the Thorpe–Ingold effect, leads to an increase in sequence selectivity compared to monosubstituted PO.^[^ ^65^, ^66^ ^]^ Notably, IBO does not contain diastereotropic protons, simplifying ^1^H NMR analysis that again supports an increase in longer oligosulphide links with sulphur loading (run #8, see ESI Figure S89) by this complementary technique. Moving to SO (run #9 and #10), an intrinsically challenging monomer in this class of polymerisation also results in successful polymer formation, albeit with no head‐to‐head or tail‐to‐tail regioselectivity. As it has been previously reported that SO ring opening occurs both at the head CHPh and tail CH_2_ positions of the epoxide, this demonstrates that the regioselectivity of the initial epoxide ring opening explains the head‐to‐head‐*alt*‐tail‐to‐tail regioselectivity observed for PO and others epoxides, thus further supporting the mechanistic hypothesis.^[^ ^69^ ^]^

Employing naphthalic thioanhydride (NTA), featuring a larger aromatic system compared to PTA, improves sequence selectivity both in combination with PO (run #11) and CHO (run #12). We attribute this to the larger aromatic system favouring O/S exchange by stabilisation of the accumulating negative charge on the carbonyl centre during backbiting. Furthermore, polymers derived from NTA exhibit increased glass transition temperatures compared to those from PTA and thereby result in a maximum *T*_g_ of 128 °C. Conversely, moving to electron richer aliphatic thioanhydrides, such as stearic thioanhydride (STA), does not result in any reactivity at all, completing the mechanistic picture that ROTERP is best and most selectively achieved for monomers that favour the backbiting step for either steric or electronic reasons. Epoxides bearing heteroatom containing and functional substituents are likewise tolerated, such as glycidyl ethers bearing an ethyl (EGE, run #13) and phenylether (PGE, run #14) substituent. Glycidyl ethers are particularly attractive as they allow for the use of natural product derived epoxides such as vanillin glycidyl ether (VGE, run #15), which likewise undergo ROTERP, producing a terpolymer with functional pending aldehyde substituents useful in, e.g., postfunctionalisation reactions (vide infra). Also, in this case NTA (run #16) increases glass transition temperatures and sequence selectivity compared to PTA.

Demonstrating the high functional group tolerance of the methodology, we employed a carbazole functionalised epoxide (run #17), producing a high *T*_g_ polymer, which, due to the intrinsic luminescence of carbazole, fluoresces under UV light. As for SO, regio unselective epoxide ring‐opening renders the polymer regio‐random. Even epichlorohydrin bearing electrophilic CH_2_Cl groups is tolerated, albeit with decreased molecular weight and broadened polydispersities (run #18), indicating some branching due to co‐occurring nucleophilic substitution with the side chains.^[^ ^70^ ^]^ Motivated by this circumstance, we employed a bifunctional epoxide DEO (run #19), which indeed yields an insoluble network with PTA and S_8_. We could even extend this to epoxidised soybean oil, yielding a crosslinked network with a very low *T*_g_ of −40 °C. Despite the insolubility of these networks in good solvents for linear terpolymers (THF, CHCl_3_) they can be compression moulded into brittle films at 80 °C due to the dynamicity of the incorporated ‐S_x_‐ bonds.

Considering the combined monomer scope, our methodology can yield polymers with ‐S_x_‐ functionalities across an impressive *T*_g_ range of ca. 170 °C. Particularly high *T*_g_ polymers exceeding 100 °C have been challenging to obtain from elemental sulphur, as, e.g., inverse vulcanisation techniques typically yield low *T*_g_ materials.^[^ ^71^, ^72^ ^]^

**Figure 6:**
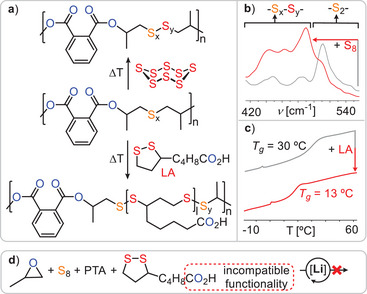
a) Thermal backbone editing of S_8_/PTA/PO terpolymer with S_8_ or lipoic acid (LA). b) Zoom into the S_x_‐ region of the Raman spectrum (black) before and (red) after thermal S_8_ insertion (10 w%). c) DSC data (black) before and (red) after lipoic acid insertion (50 w% feed ratio). d) Attempted polymerisation of LA during ROTERP fails.

**Figure 7:**
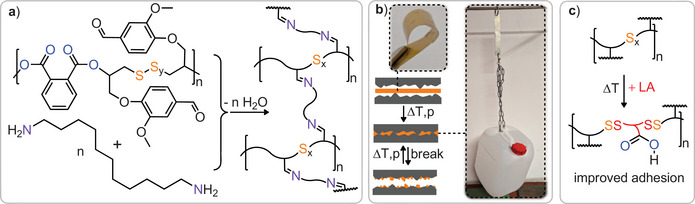
a) Crosslinking of S_8_/PTA/PO terpolymer via imine condensation with 1,12‐dodecyldiamine. b) Application of compression mouldable polymer films as adhesives on steel with photographs of bonded steel plates holding a filled water canister. c) Schematic representation of backbone edited material obtained from lipoic acid insertion, which improves adhesion on aluminium.

In terms of thermal stability, the vast majority of materials are stable to above 250 °C which represents an approximate 100 °C improvement over previous ROTERP polymers from elemental sulphur, which were prone to decomposition via loss of COS as soon as sulphur–sulphur bonds opened.^[^ ^31^ ^]^ In contrast, the polymers reported in this contribution are stable at temperatures where sulphur–sulphur bonds can be thermally activated. Hereby the polymer backbone becomes amenable to modifications, a concept which recently attracted attention under the term “skeletal or backbone editing”.^[^ ^73^, ^74^, ^75^ ^]^ In order to modify the polymer backbone, we heated a finely ground mixture of a PO terpolymer (Table 1 run #9) and 5 or 10 w% elemental sulphur at 140° for 120 min in an attempt to incorporate further sulphur into the polymer. Assessing the ^1^H NMR spectrum indeed shows diagnostic changes of the CH_2_‐S_x_‐ region with signals increasing in relative intensity that correspond to longer oligosulphide chains (Figure 6b). This observation could be more clearly observed in the ^1^H NMR spectrum of the IBO terpolymer, which does not feature diastereotopic protons (see ESI, Figure S136). Raman spectroscopy confirms this observation. As shown in Figure 6, while the Raman band at 510 cm^−1^ corresponding to disulphide links is most intense in the starting polymer, this decreases in intensity upon reaction with elemental sulphur with bands below 490 cm^−1^ corresponding to higher order oligosulphides increasing in intensity; these observations are more pronounced at 10 w% than at 5 w% elemental sulphur in the starting mixture. GPC analysis confirms that the polymer remains intact upon sulphur insertion with *M*_w_ shifting from 28 kDa (*Đ = 1.6*) to 29 kDa (*Đ = 1.6*) with 5 w% S_8_ and 23 kDa (*Đ = 1.7*) with 10 w% S_8_, indicating some chain cleavage or rearrangement via sulphur bond metathesis in the process. Backbone editing could also be expanded to cyclic disulphide, such as lipoic acid. Hence, we heated a 1:1 mixture in w% of lipoic acid with an S_8_/PTA/PO terpolymer at 140 °C for 240 min followed by precipitation of the product mixture to remove unconsumed lipoic acid. The so‐formed polymer exhibits additional resonances in its ^1^H NMR spectrum in the aliphatic region between 1.8 and 3.6 ppm (see ESI Figure S138). These correspond to ring‐opened lipoic acid and are broadened and shifted relative to the starting lipoic acid reactant in 47% of the repeat units by integration. Editing of the polymer backbone is also supported by a redistribution of the Raman S_x_ bands (see ESI Figure S139). GPC analysis shows slightly broadened molecular weight distribution (see ESI Figure S141), which we again attribute to chain cleavage and rearrangement via sulphur–sulphur bond metathesis. As can be seen in Figure 6c, insertion of lipoic acid does decrease the glass transition temperature from 30° to 17 °C. Hence, backbone editing provides a means to alter the thermal properties after synthesis. Notably this also allows the incorporation of functional groups which are intrinsically incompatible with the polymerisation methodology, as attempting to perform ROTERP in the presence of lipoic acid (Figure 6d) results in no reaction at all (Figure 7).

The large monomer scope of the ROTERP methodology enables the incorporation of functional groups into the polymer side chains that also allow for post polymerisation modification of these. In this regard the S_8_/PTA/VGE terpolymer (Table 1 run #15) features aldehyde functionalities that can undergo imine condensation, offering an opportunity for the incorporation of degradable crosslinks to enhance mechanical properties.^[^ ^76^, ^77^, ^78^ ^]^ The reaction of the VGE terpolymer (Table 2 run #14) with (see ESI Methods section) leads to gelation of the reaction mixture. Removal of the solvent yields a solid that is entirely insoluble (> 99% gel fraction) in common organic solvents which the starting linear polymer is soluble in (DCM, THF, DMF), clearly substantiating crosslinking (see ESI Figure S146). However, due to the dynamicity of sulphur–sulphur and imine bonds, this network can be processed by compression moulding into flexible films that exhibit a maximum stress of 49.9 ± 4.6 MPa at 2.7% ± 0.3% elongation at break (see ESI Table S3). The thermally reprocessed material recovers 88% and 77% of maximum stress and strain resistance respectively. The potential for thermal reprocessing of these networks enables their application as reusable adhesives as has been for example demonstrated for polymers from inverse vulcanisation.^[^ ^18^, ^21^, ^79^ ^]^ In contrast to other approaches to such networks, our stepwise access allows to combine polymer links during synthesis to rationally impart functionality onto the material. Accordingly, to evaluate the adhesion performance of the imine crosslinked polysulphide, it was tested on wood as well as abraded steel and aluminium sheets (100 × 25 mm) with the polymer applied to an overlapping area of 25 × 16 mm, and the two sheets were bonded together by compression moulding (see ESI methods section). Lap shear experiments were subsequently performed at a pull rate of 5 mm min^−1^ showing a typical maximum stress of 5.4 ± 1.2 MPa on steel (three run average, both adhesive and cohesive type failure). These performance properties are competitive, when compared to other sulphur based adhesives reported previously and, for example, exceed the performance of commercial glues such as epoxy glue reference (see ESI Table S3).^[^ ^18^, ^21^, ^79^, ^80^, ^81^, ^82^ ^]^ Importantly, after material failure, the respective parts can be rebound by compression moulding under restoration of the adhesion performance, achieving 6.6 ± 1.1 MPa. We attribute the slight increase in performance to some curing occurring via completion of imine condensation during reprocessing. Adhesive testing on wood reveals that material failure of the wood specimen occurs before failure of the adhesive. On aluminium, however, a lower maximum stress of 2.8 ± 0.8 MPa (adhesive type failure) was achieved. We opted to improve performances by the introduction of polar groups which are expected to aid interaction with the substrate surface and applied the above established backbone editing protocol, as lipoic acid features polar carboxylic acid groups. Gratifyingly, heating a finely ground mixture of the crosslinked material with 20 w% lipoic acid at 140 °C results in the formation of a homogenous material. Only trace amounts of lipoic acid could be identified in the sol‐fraction of a sol‐gel test supporting incorporation into the network. Although Raman spectroscopy was prevented by the high absorbance of the material, differential scanning calorimetry (DSC) shows a decrease in *T*_g_ from 53° to 36 °C further substantiating incorporation (see ESI Figure S152). Testing the modified material as an adhesive on aluminium gratifyingly reveals a more than threefold improvement of adhesion performances with a stress at break of 5.6 ± 0.3 MPa (cohesive type failure). Hence, the backbone modifiability can be used to improve material performance.

Yet not only the S_x_ bonds but also the imine crosslinks provide additional functionality to the material: they impart acid degradability. As can be seen in the supplementary video, exposing the adhesive to hydrochloric acid results in the debonding of the sheets within 30 min. Hence, the stepwise synthetic approach allows tailoring adhesives with respect to several target properties.

## Conclusions

In conclusion, we have reported a new sequence selective terpolymerisation of elemental sulphur delivering polyester featuring dynamic oligosulphide units. Sequence selectivity is achieved on kinetic grounds in which coordinative participation of polyester links adjacent to the chain end leads to a rate increase, as they represent better donors. The exceptionally broad monomer scope gives straightforward access to a wide glass transition temperature range as well as functional side chains. The improved thermal stability renders the backbone editable via insertion into the ─S─S─ bonds. Crosslinking of aldehyde functionalised polymer via imine condensation yields thermally reprocessable adhesive in which backbone editing can be used to enhance adhesive performance while the imine bond grants acid degradability. Our contribution demonstrates how mechanistic understanding achieves to impart the dynamicity of elemental sulphur into a popular class of polymers, namely polyester, with tuneable properties and functionality.

## Supporting Information

Additional references cited within the Supporting Information.^[^ ^83^, ^84^, ^85^, ^86^, ^87^, ^88^ ^]^

## Conflict of Interests

The authors declare no conflict of interest.

## Supporting information



Supporting Information S1

Supporting Information S2

## Data Availability

The data that support the findings of this study are available in the supplementary material of this article.
