# Supplementary material for: Kinetically Enhanced Access to a Dynamic Polyester Platform via Sequence Selective Terpolymerisation of Elemental Sulphur
Source: Angew Chem Int Ed Engl. 2025 Jun 4;64(30):e202501337. doi: 10.1002/anie.202501337 (PMC12281093; doi:10.1002/anie.202501337)
Supplement: Supplementary file 1 — Supporting Information S1 [file ANIE-64-e202501337-s002.pdf]

Supporting information for

Kinetically Enhanced Access to a Dynamic Polyester Platform via Sequence Selective Terpolymerisation of Elemental Sulfur

Section S1: Methods……………………………………………………………………….….2

Section S2: Polymerisation and post functionalization procedure………….…….….…4

Section S3: S_8/PTA/PO ROTERP……………………………………………………….…...6

Section S4: Monomer scope…...……………………….………………………….….…15

Section S5: Post functionalization ………………………………………….…………….....70

Section S6: DFT calculations 81

Section S7: References………………………………………………………………….…….88

Section S1: Methods

Solvents and reagents were obtained from commercial sources and used as received unless stated otherwise. NMR spectra were recorded by using a Jeol JNM-ECA 400II, Bruker Advance 600 and 700 MHz spectrometer. ^{1}H and $^{13}C\{^{1}H\}$ chemical shifts are referenced to the residual proton resonance of the deuterated solvents. Epoxides were dried over calcium hydride at room temperature for 2 days followed by distillation under argon atmosphere. BnOH was dried over 4Å molecular sieves and degassed. Elemental sulfur (99.99% purity) was sublimed under dynamic vacuum at 100°C. Phthalic thioanhydride (PTA) was synthesized according to the literature procedure and then purified by recrystallisation from ^{t}BuOMe followed by recrystallisation from $CHCl_3$ and two sublimations under dynamic vacuum at 90°C and was stored inside an argon filled glovebox prior to use.[1] VGE (vanillin monoglycidyl ether) and 9-(Oxiran-2-ylmethyl)-9H-carbazole (CE) were prepared following previously reported procedures.[2],[3] Naphthalic thioanhydride (NTA) was prepared following the synthetic procedure used for Phthalic anhydride (PTA) with the difference that the product precipitates from the solution. It was isolated via filtration and washed with boiling H_2O. Characterisation agreed with the literature.[4] The obtained crystals were sublimed under vacuum at 120°C and stored in the glove box. All other reagents were used as received if not stated otherwise. All reagents were stored and used inside an Ar filled glovebox.

A confocal WITec Alpha 300 RA + Raman imaging system equipped with a UHTS 300 spectrometer and a back-illuminated Andor Newton 970 EMCCD camera together with the WITec Suite SIX 6.1 software package were employed for Raman measurements. All spectra were acquired with an excitation wavelength of λ = 532 nm, using integration times of 0.3–0.35 s and a laser intensity of 20 mW for Raman imaging. For large area Raman imaging (typically 180 × 150 μm^2) a 50× long working distance objective (Zeiss LD EC Epiplan-Neofluar Dic 50×, numerical aperture NA = 0.55) and a step size of 0.5 µm $pixel^{-1}$ were used. Raman imaging with a 100× objective (Zeiss EC Epiplan-Neofluar Dic 100×, NA = 0.9, typically 50 × 50 μm^2) was conducted with a step size of 0.2 µm $pixel^{-1}$. All spectra were corrected for cosmic ray spikes and subjected to a background removal routine.

TGA data was measured using a Netzsch TG 209. DSC was measured on a Netzsch 204 F1 “Phoenix” at 10 K/min heating rate.

Size exclusion chromatography (SEC) was performed using a Waters 515 HPLC pump and THF as eluent at a flow rate of 0.5 mL/min. A volume of 100 µL of polymer solution (1-2 mg/mL) was injected with a 707 Waters autosampler into a column setup comprising a guard column (MesoPore Guard 5 x 0.75 cm, particle size 3 µm). Polymer size distribution was monitored with a Waters 414 refractive index detector. Narrow distributed polystyrene standards were used for calibration and 1,2-dichlorobenzene as internal reference. Each polymer sample was dissolved in HPLC-grade THF (2 mg/mL) and filtered through a 0.45 µm porous filter frit prior to analysis. Elmann’s reagent was added to the mixture before filtration, this was done to end cap the polymer chains and prevent depolymerization in THF.

Polymer films were made using a Weber Hot press at 130°C and 5 metric tons for 30 min, after that the plates were left to cool down slowly while keeping the pressure, that way we assured to have flat films and avoid any bending or cracking due to inhomogeneous cooling.

Tensile tests were performed on an Instron 5565 universal tester using a 1 kN load cell and pneumatic clamps. The thickness of the specimens was measured with a digital micrometre (Mitutoyo 293–831, digimatic MDC Lite) in the range of 600 to 650 µm. The E-Moduli of all specimens were investigated at a strain rate of 0.2 mm min^{-1} and calculated between 0.1% and 0.3% strain. To ensure a better evaluation of the properties, the measure was conducted on 3 dog bones cut from the same film.

Lap shear test was run on a universal testing machine Zwick 1485 with a pull rate of 5 mm/min. Samples of 100x25 mm with an overlay of 25x16 mm were tested.

In situ IR measurements were done with a ReactIR 15 from Mettler Toledo equipped with a DST Series AgX Fiber Conduit probe. All monomers were dissolved in an excess of epoxide and preheated at 80°C to ensure dissolution. Once the mixture was at the required temperature, the baseline was set and after that a solution containing the catalyst was injected and the evaluation started. Due to the necessity of running this reaction at 80°C, Ethyl glycidyl ether (EGE) was chosen as the epoxide due to its' increased boiling point compared to propylene oxide.

Section S2: Polymerisation and post functionalization procedure

General Polymerization Protocol: Inside an argon-filled glovebox, LiHMDS and BnOH were dissolved in the epoxide, and the mixture was transferred to an oven-dried vial equipped with an oven-dried stirrer bar. S_8 and PTA were then added, and the vial was sealed with a melamine cap containing a Teflon inlay. The vial was then brought outside the glovebox, placed in a pre-heated aluminum block at the specified temperature, and maintained for the specified time. To ensure reproducibility, the polymerization was conducted at 30°C, representing "room temperature" during summer.

At the end of the reaction, the polymer was isolated by dilution in DCM (5 mL), filtered through a 0.5 µm syringe filter to remove traces of unreacted solids, such as elemental sulfur, and then the mixture was successively added to 40 mL of MeOH. This caused the polymer to precipitate making it possible to isolate it through centrifugation.

To ensure the stability of the samples during GPC measurements, Ellman's reagent (ca. 10 mg/mL) was added to the THF solutions for analysis to quench reactive thiol chain ends. [5][6] Note that if this step is omitted, slow depolymerization occurs during GPC measurements.

Metathesis experiment: An isolated polymer obtained from isobutylene oxide (IBO, due to more facile NMR analysis of the S_x region) was dissolved in PO (to model the medium during polymerization), subsequently an excess (0.2 mL) of dimethyl trisulfide was added to the solution and the mixture was heated to 100° for 3h before precipitation with MeOH. Both the crude mixture and the precipitated polymer were analyzed with GPC to evaluate the changes in the molecular weight. Note that recently spontaneous trisulfide metathesis has been reported.[7]

Insertion protocol: The terpolymer was frozen in liquid nitrogen and manually grinded to a powder, after that it was left in a drying oven to remove any water that condensed on it during the cooling process. Once the powder was dried, it was mixed with different amount of sulfur or lipoic acid and the whole mixture was heated to 140°C to melt the components and enable polysulfide activation. After 2h for sulfur and 4h for lipoic acid, the mixture was cooled to room temperature, dissolved in DCM and precipitated with MeOH followed by isolation via centrifugation.

Cross linking protocol: The VGE terpolymer was dissolved in DCM and 1,12-dodecandiamine was added. Knowing that for each repeating unit of polymer there are 2 aldehyde groups stemming from VGE, we added 0.9 eq. of diamine per repeat unit to avoid partially reaction of diamines. The mixture was left stirring at room temperature overnight and afterwards opened to the atmosphere. Once DCM was completely evaporated, the network was dried at 80°C overnight under vacuum to further promote imine condensation through the removal of water.

Lipoic acid insertion into cross linked material: The network was grinded with the aid of a cryo-ball mill (-195.8°C, 25 mm diameter sphere, frequency of 30 oscillation per second). The resulting powder was then mixed with lipoic acid, and the mixture was heated at 80°C for 30 minutes to melt lipoic acid and allow the liquid to swell into the network powder. After that, the mixture was heated at 140°C for 2h to promote disulfide activation allowing the insertion lipoic acid in the network. The resulting material was then hot pressed at 130°C to promote further curing.

Adhesion test: 3 different substrates were tested: Steel S235, aluminium and oak wood (total area 100×25 mm with an overlapping area of 25×16 mm). For metals, the plates were first abraded with sandpaper to remove any oxidized layer and promote interaction with the surface. This was followed by cleaning with acetone. After that the polymer film was placed in between the two plates that were then hot pressed under 2 metric tons at 130°C for 30 min. After that, the temperature was switched off and the plates were left to cool down slowly to room temperature. Since the wood plates were too fragile to be hot pressed under the same condition, adhesion was achieved by clamping the two plates together and then the whole setup was left inside an oven at 125°C. After 30 min clamps were readjusted and then the setup was left in the oven overnight. Excess polymer that squeezed out was removed with a scalpel. The materials were also compared with a commercial epoxy glue: WEICON – epoxy minute adhesive. In this case the sealant was also applied after abrading and cleaning, the bicomponent epoxy glue was put on one side of the plate and then the other side was pressed under 2 metric tons at room temperature for 30 minutes. In each case the performance and associated errors were calculated from 3 distinct samples.

Section S3: S_8/PTA/PO ROTERP

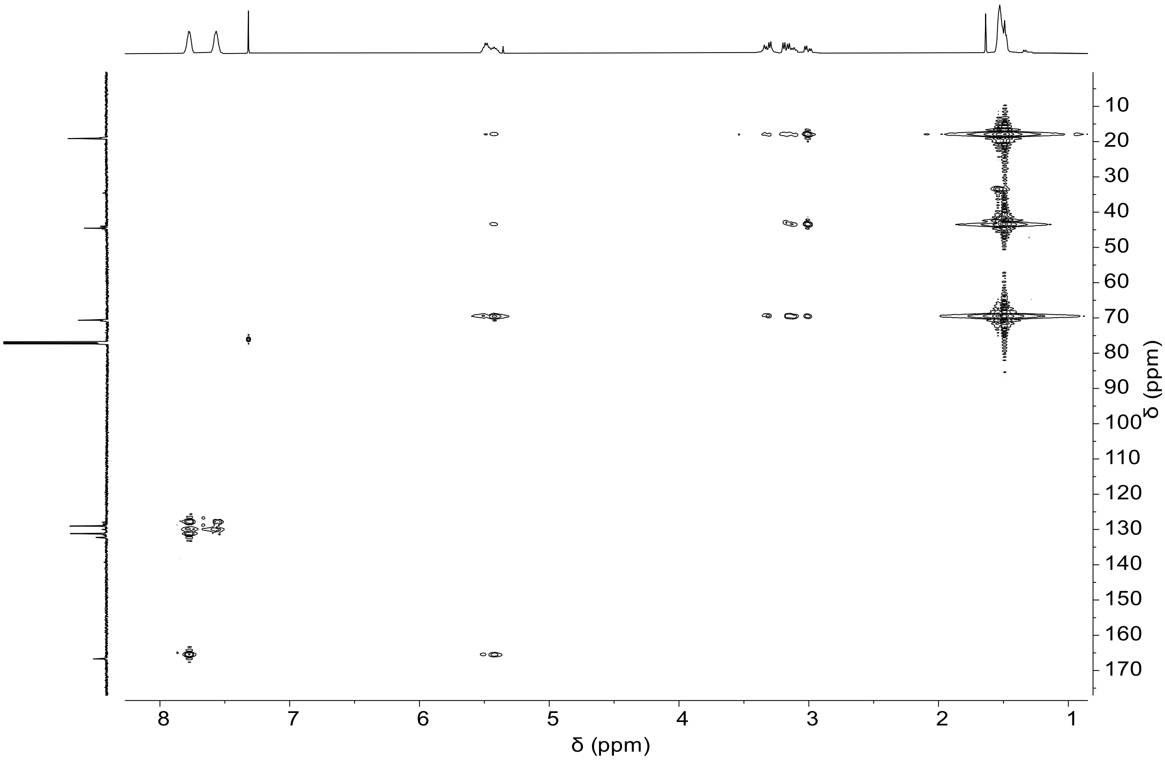

Figure S1: ^{1}H-^{13}C HMBC NMR spectrum (400 MHz, $CDCl_3$, 25°C) of the precipitated polymer corresponding to table 1 run #5.

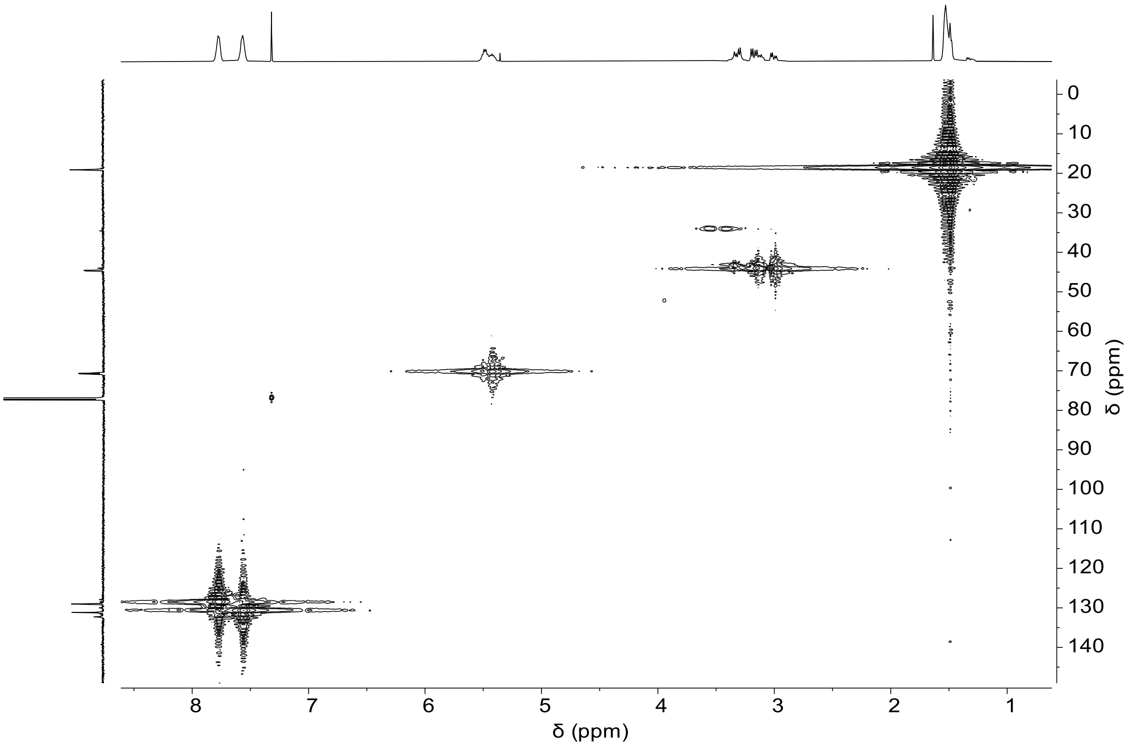

Figure S2: ^{1}H-^{13}C HSQC NMR spectrum (400 MHz, $CDCl_3$, 25°C) of the precipitated polymer corresponding to table 1 run #5.

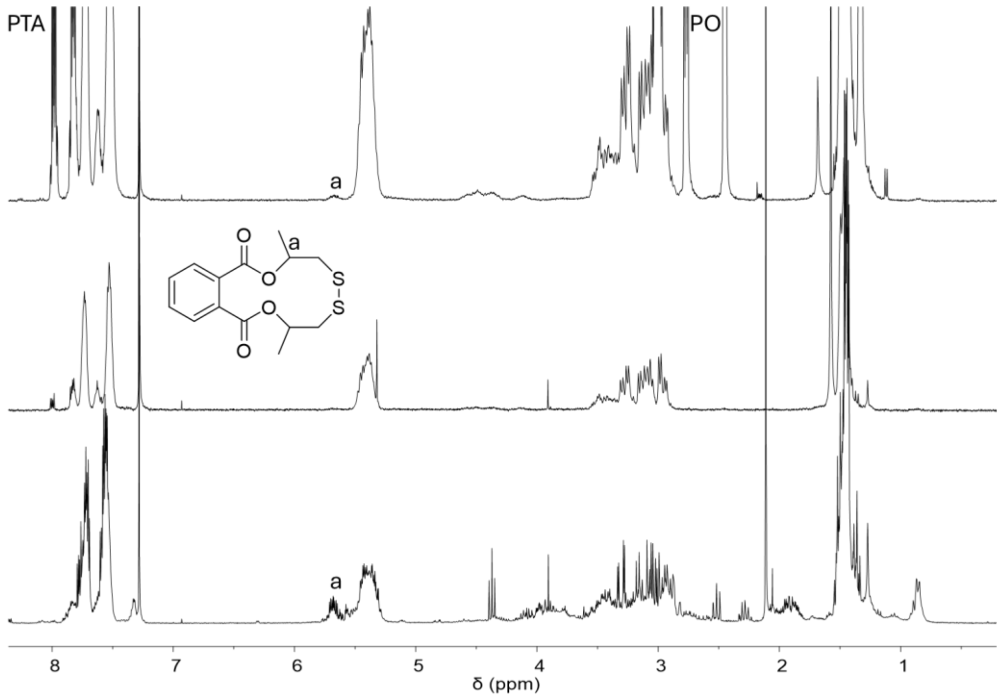

Figure S3: ^{1}H-NMR spectra of (top) crude mixture (b) isolated terpolymer and (c) depolymerization attempt. For the depolymerization experiment thiolate species (5 mol% per repeat unit of polymer) were prepared from benzyl mercaptan and LiHMDS following by prolonged heating (reflux overnight) of an isolated terpolymer with the so generated thiolate species in THF producing resonances that correspond to macrocyclic species in line with depolymerisation hypothesis.

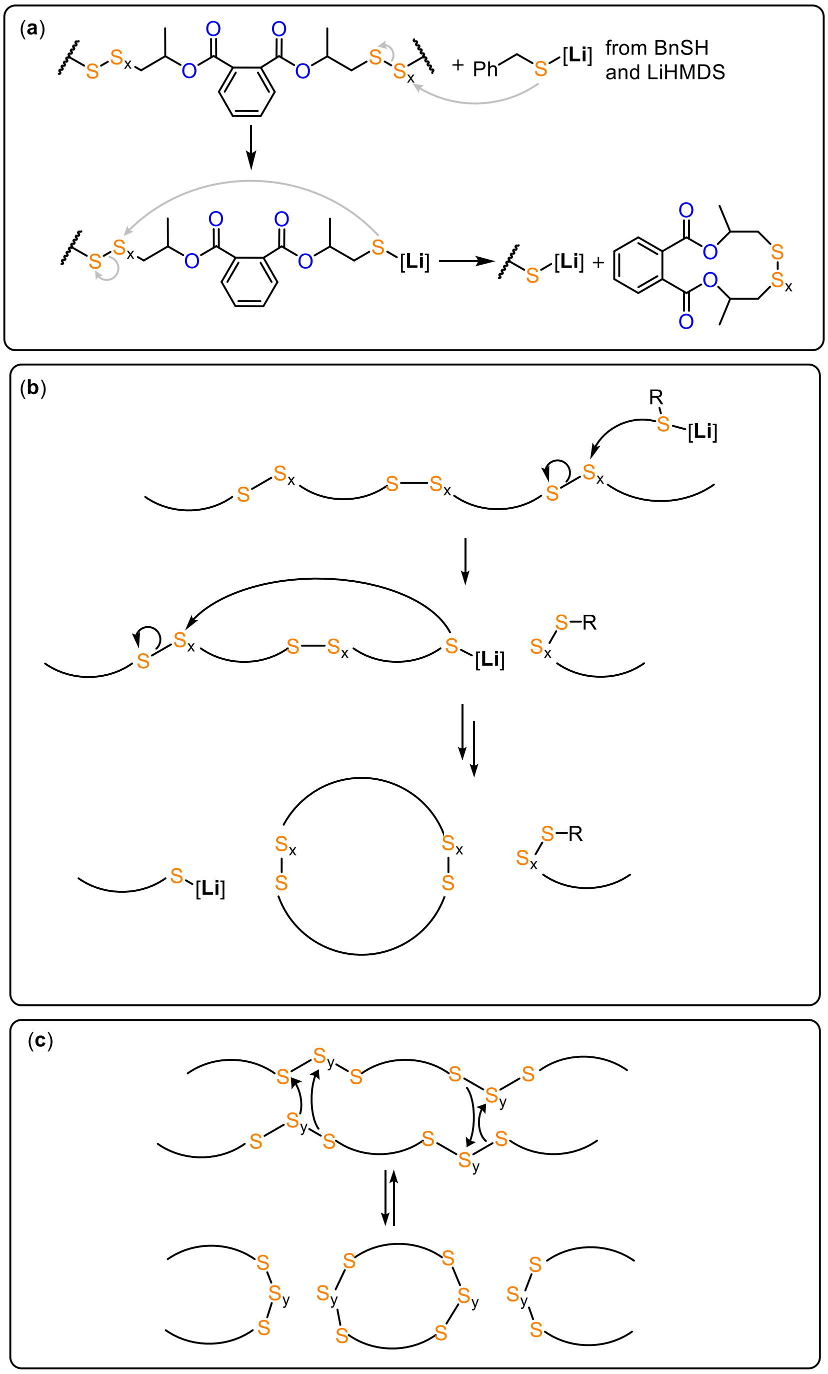

Scheme S1: Mechanistic hypothesis for (a) backbiting/depolymerisation reaction evoked by LiSBn; note that this can likewise occur from thiolates intermediates during the ROTERP cycle. (b) Formation of larger macrocycles following the same mechanism; note that these can be in principle any size including macrocyclic polymers when the attack of the thiolate occurs to very distant S_x units. (c) Macrocyclisation following the recently discovered spontaneous metathesis mechanism.[7]

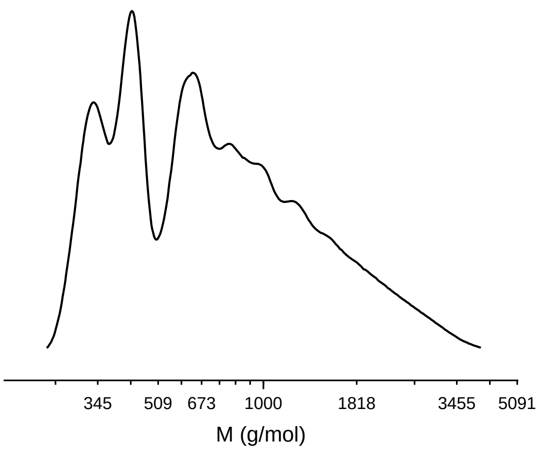

Figure S4: GPC analysis of the dried liquid phase obtained after the precipitation of terpolymer from DCM/MeOH. Molecular weight of one repeat unit S_x = S_2 is 312 g/mol (344 g/mol for S_3 , 376 g/mol for S_4, 408 g/mol for S_5). Macrocycles comprising 2 or 3 repeat units tentatively correspond peaks around 600 and 900 g/mol.

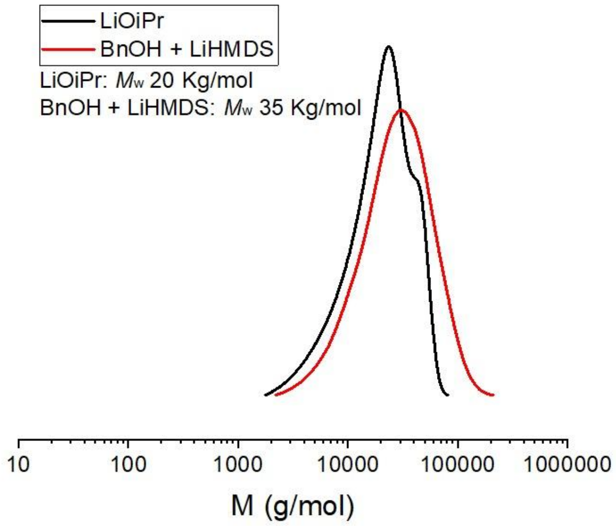

Figure S5: Comparison of the GPC signals obtained from two terpolymerisation having same loading and conditions (1 eq. LiOR: 500 eq. PO: 200 eq. PTA: 200 eq. S at 100°C) indicating that *in situ* produced $HN(SiMe_3)_2$ does not have negative effects on the polymerisation outcome.

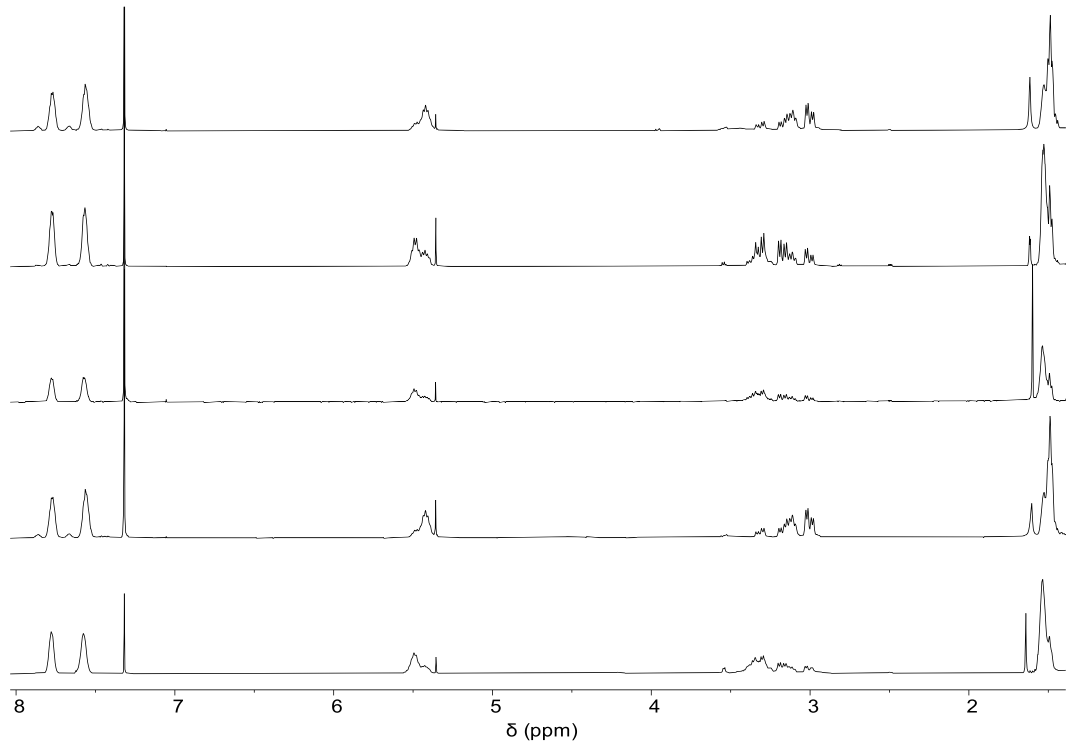

Figure S6: Overlaid ^{1}H-NMR spectra (400 MHz, $CDCl_3$, 25°C) of the precipitated polymers corresponding to table 1 run #1,2,3,4,6.

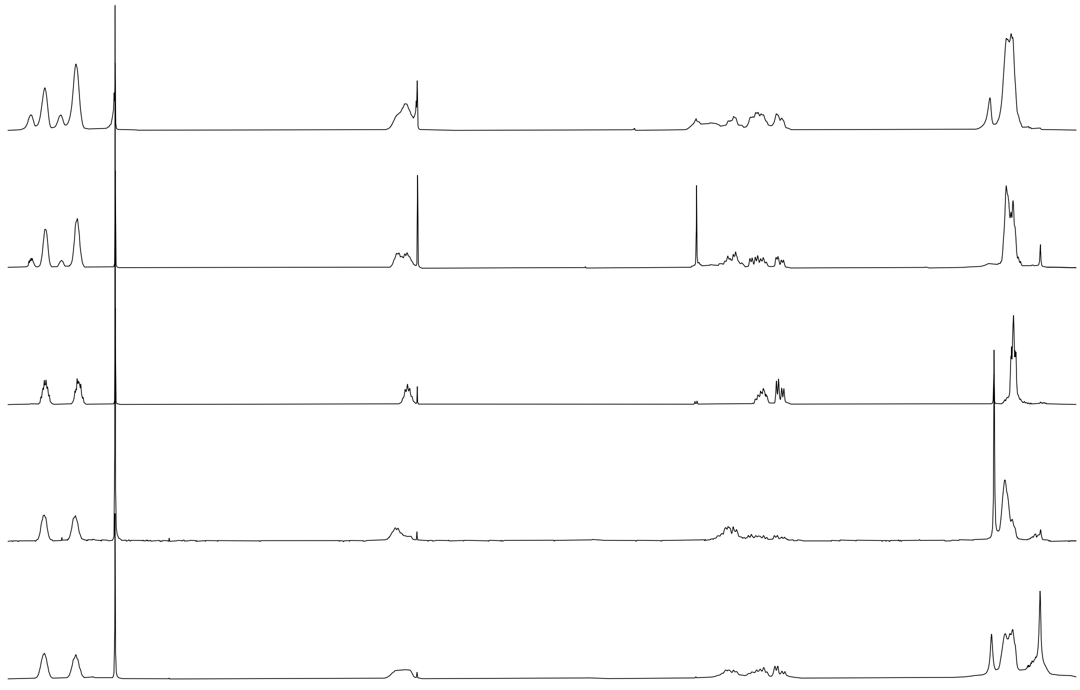

Figure S7: Overlaid ^{1}H-NMR spectra (400 MHz, $CDCl_3$, 25°C) of the precipitated polymers corresponding to table 1 run #7,8,9,10,11.

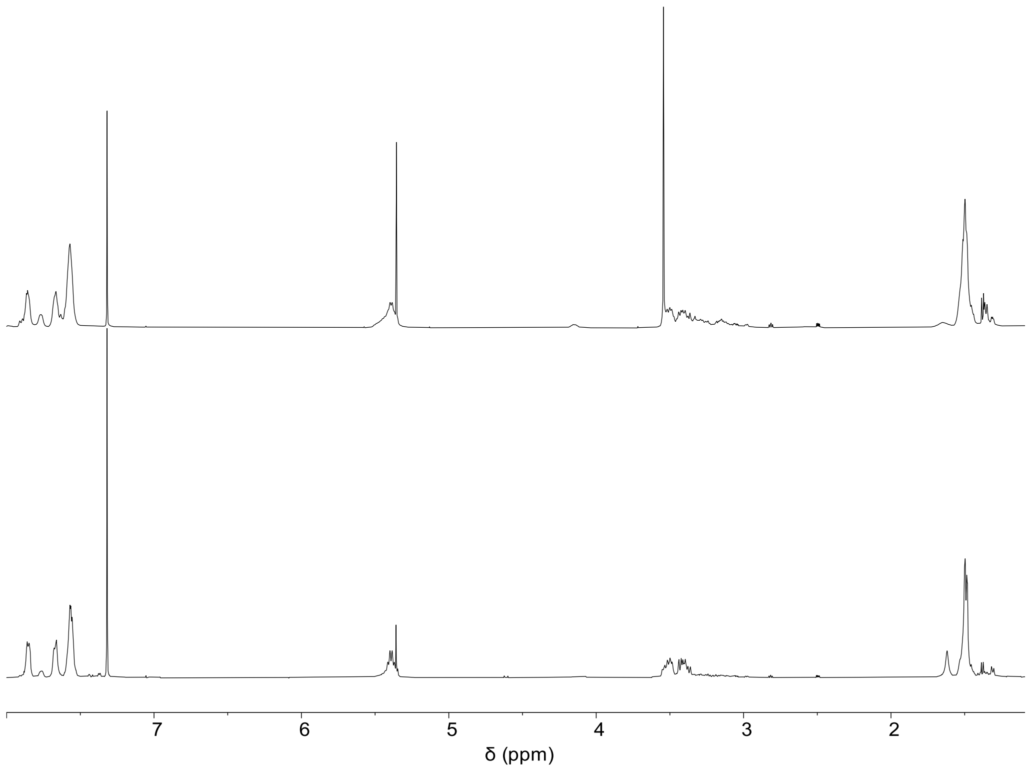

Figure S8: Overlaid ^{1}H-NMR spectra (400 MHz, $CDCl_3$, 25°C) of the precipitated polymers corresponding to table 1 run #12,13.

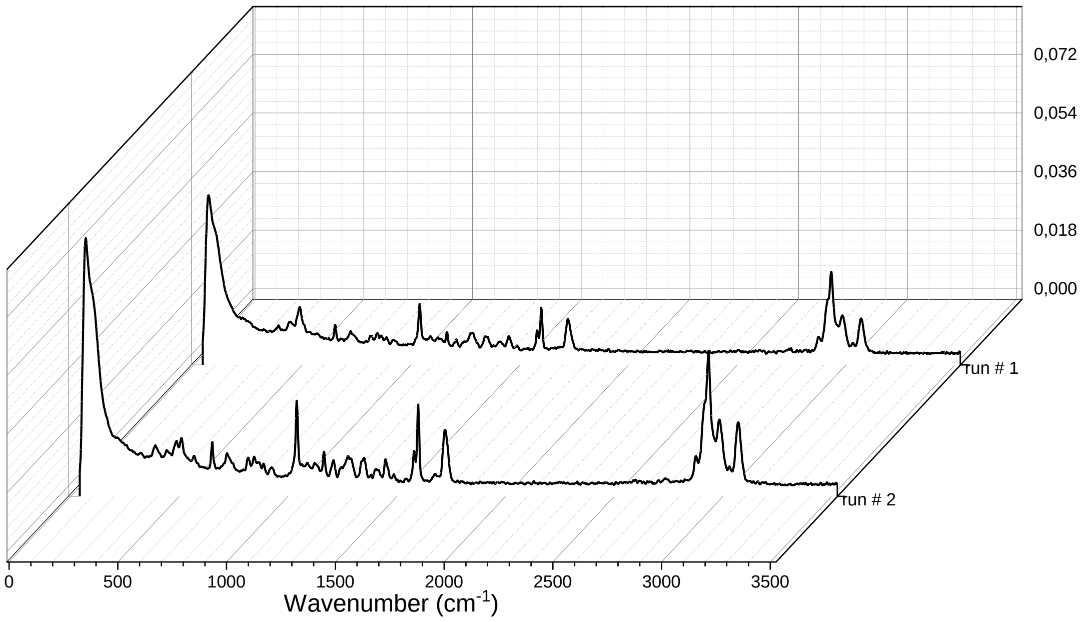

Figure S9: Overlaid Raman spectra of the polymers corresponding to table 1 run #1 and #2.

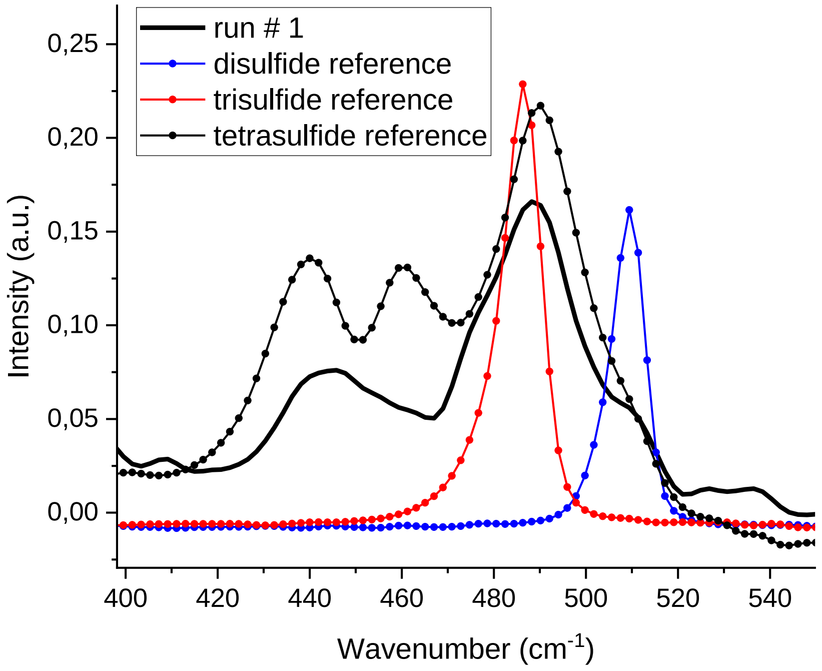

Figure S10: Zoom into the C-S_x-C stretching vibrations of the Raman spectra of the polymer corresponding to table 1 run # 1, compared with dimethyl disulfide, dimethyl trisulfide, and R_2-tetrasulfide [R= -$CH_2CH_2CH_2Si(OCH_2CH_3)_3$] reference compounds.

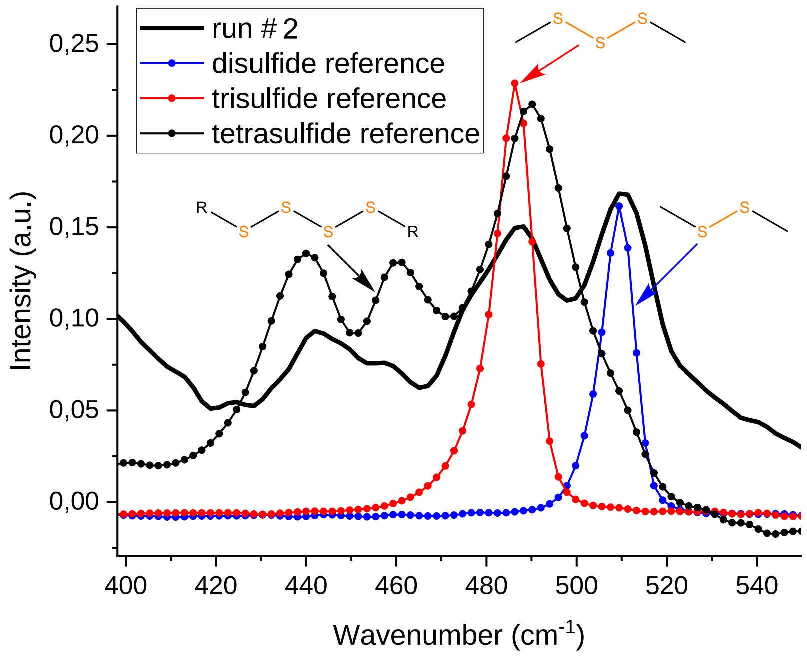

Figure S11: Zoom into the C-S_x-C stretching vibrations of the Raman spectra of the polymer corresponding to table 1 run # 2, compared with dimethyl disulfide, dimethyl trisulfide, and R_2-tetrasulfide [R= -$CH_2CH_2CH_2Si(OCH_2CH_3)_3$] reference compounds.

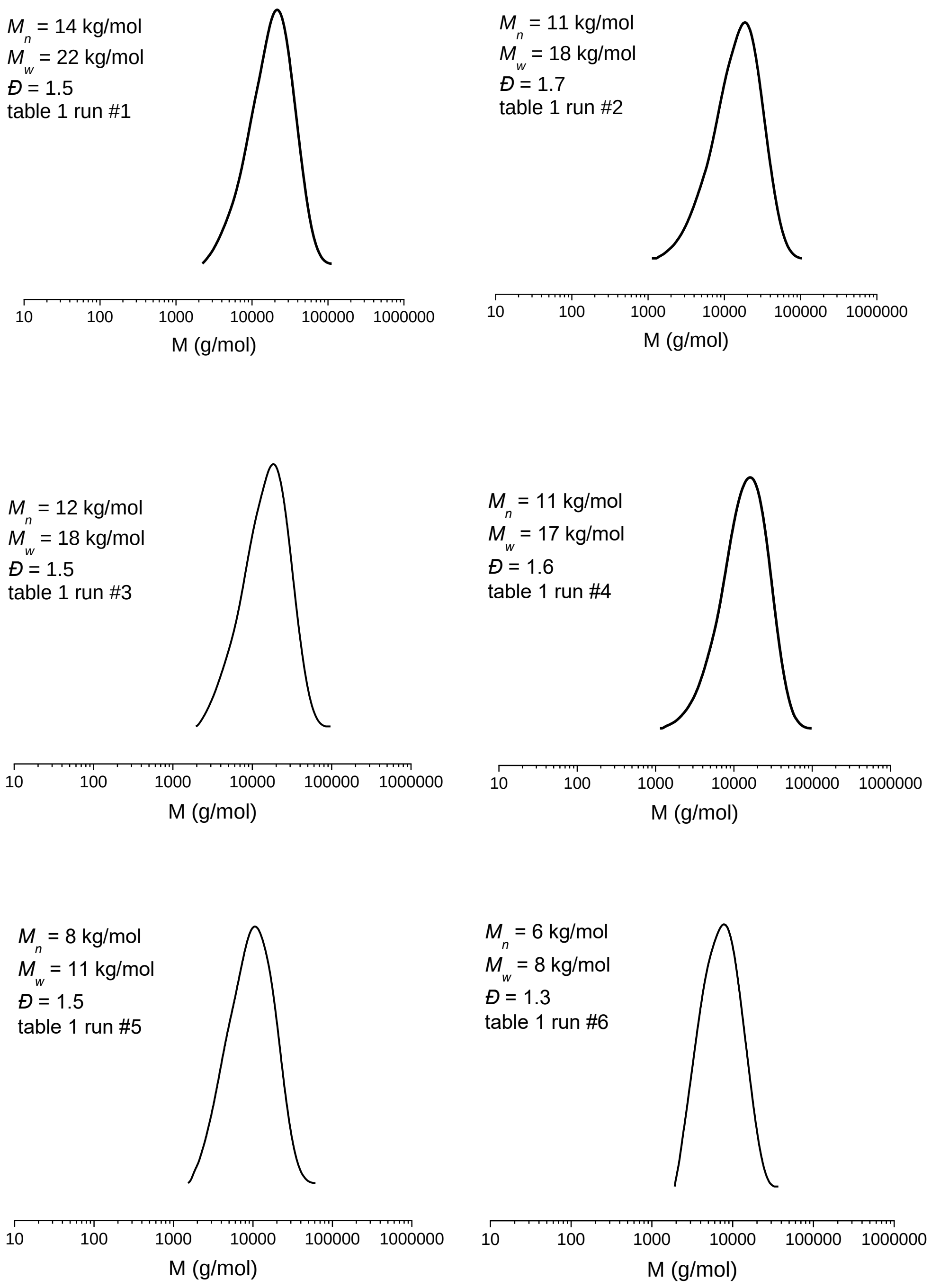

Figure S12: GPC curves corresponding to table 1 run #1,2,3,4,5,6.

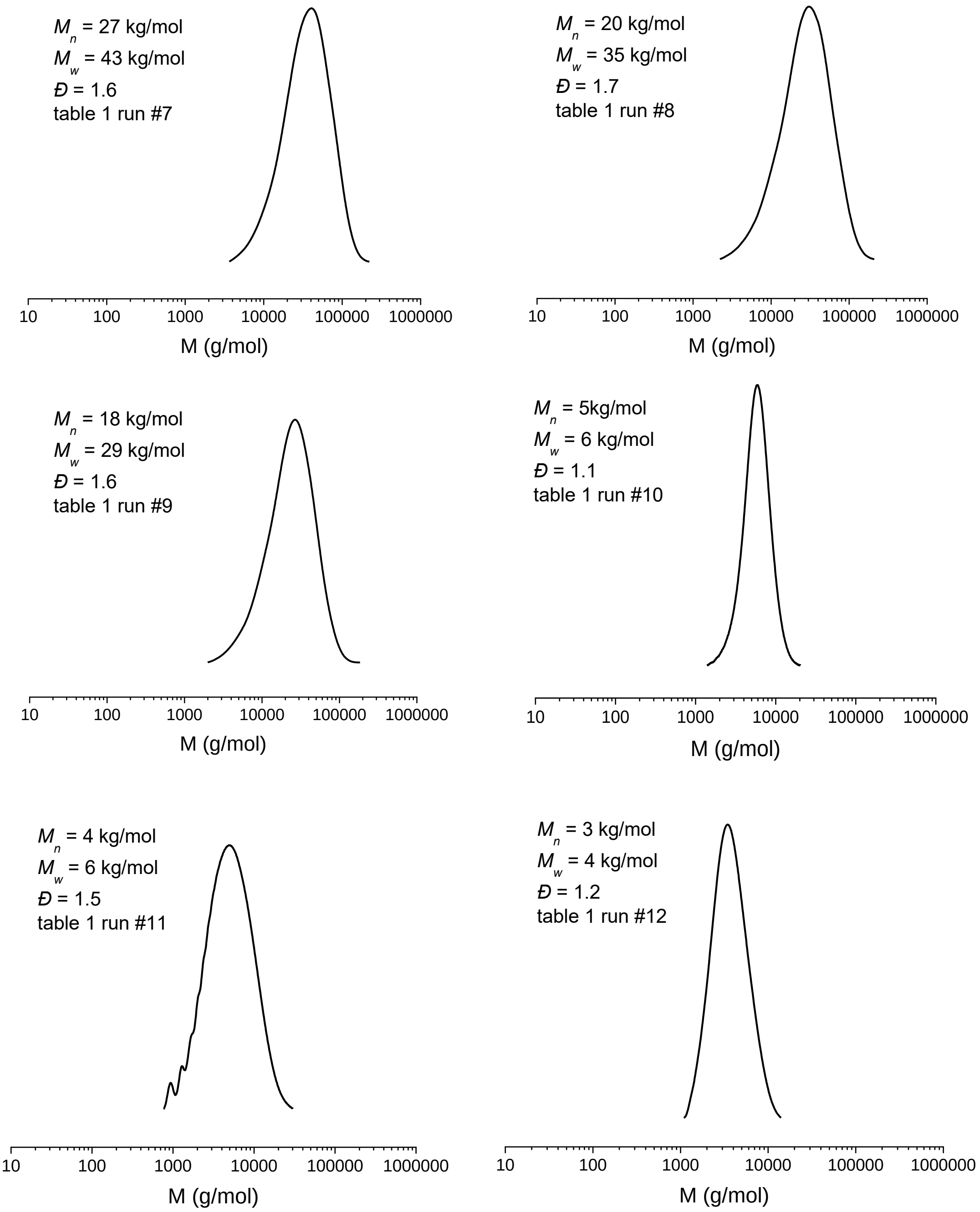

Figure S13: GPC curves corresponding to table 1 run #7,8,9,10,11,12.

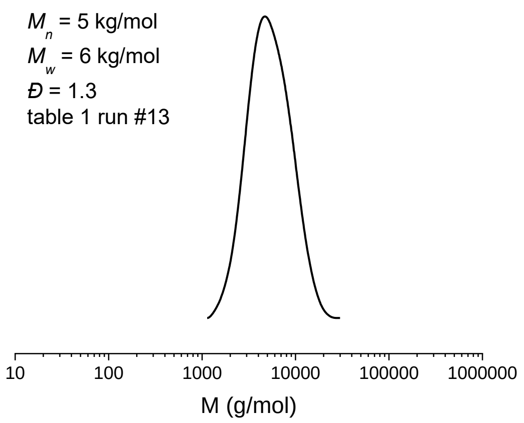

Figure S14: GPC curve corresponding to table 1 run #13.

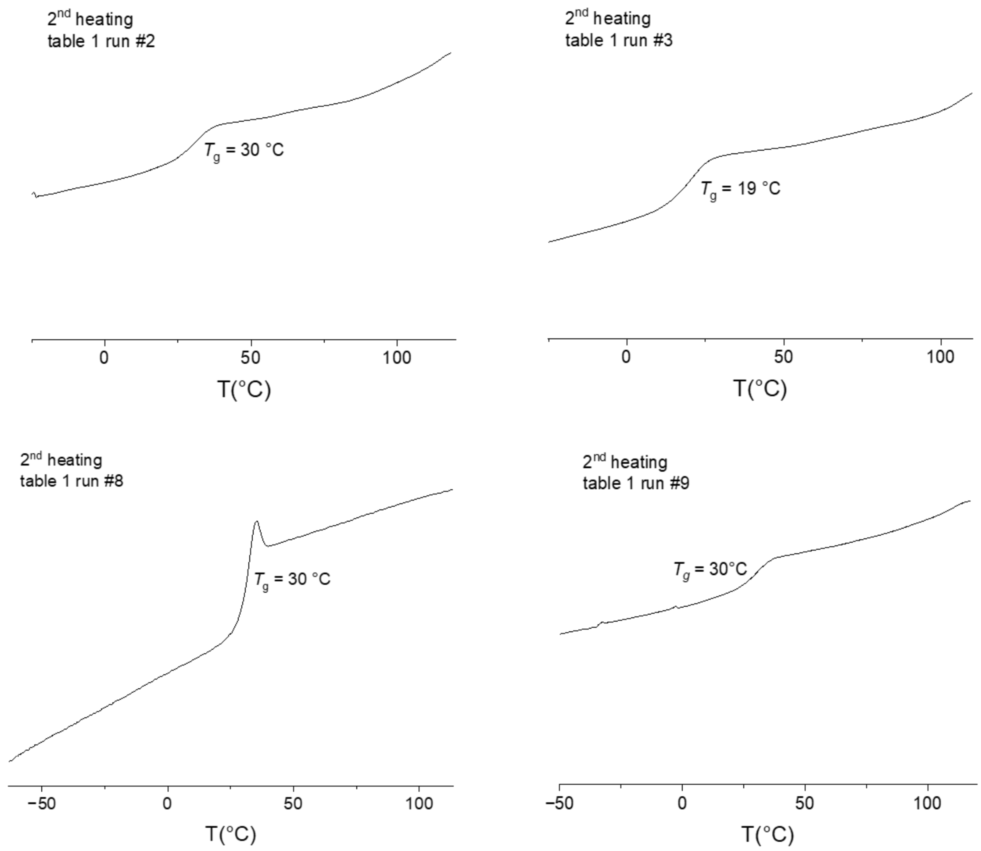

Figure S15: DSC 2nd heating curves of the polymers corresponding to table 1 run #2,3,8,9.

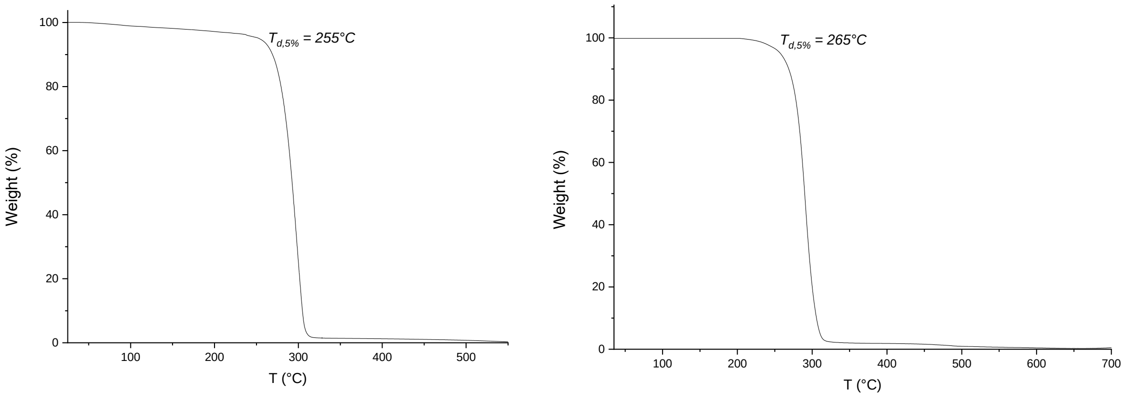

Figure S16: TGA data of the polymers corresponding to table 1 run # 1 (left) and #8 (right).

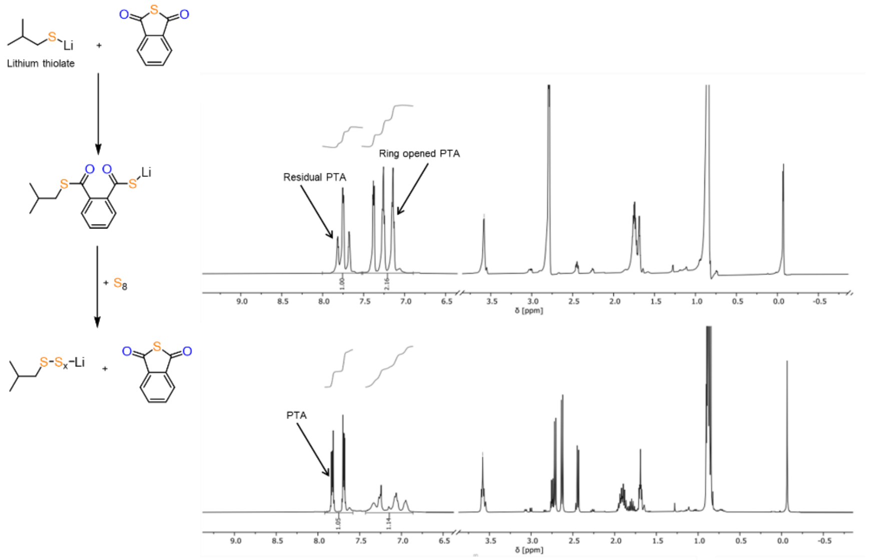

Figure S17: Zoom into the ^{1}H-NMR (400 MHz, d_8-THF, 25°C) of the model reaction between lithium thiolate, PTA and S_8.

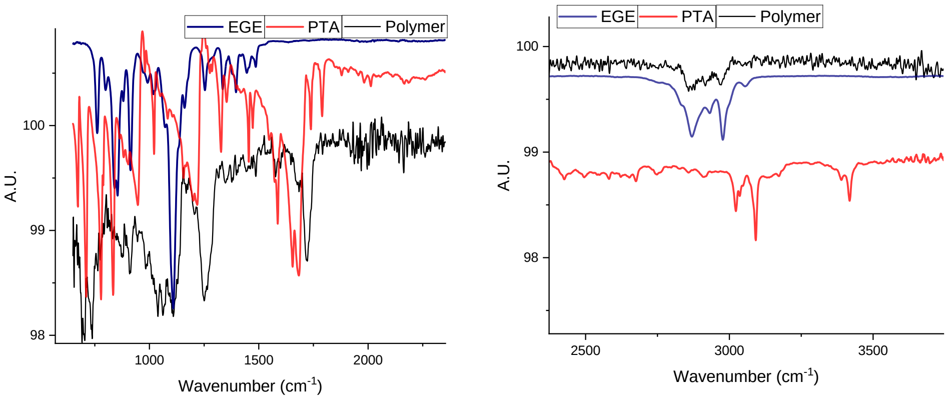

Figure S18: Overlayed IR spectra of ethyl glycidyl ether (EGE), phthalic thioanhydride (PTA) and the polymer obtained from these two monomers and elemental sulfur.

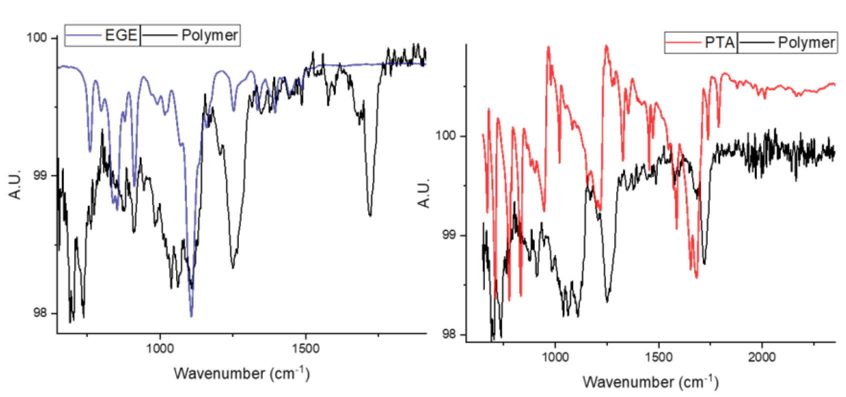

Figure S19: Overlayed IR spectra of the polymer and parent monomers. Left: polymer and EGE. Right: polymer and PTA.

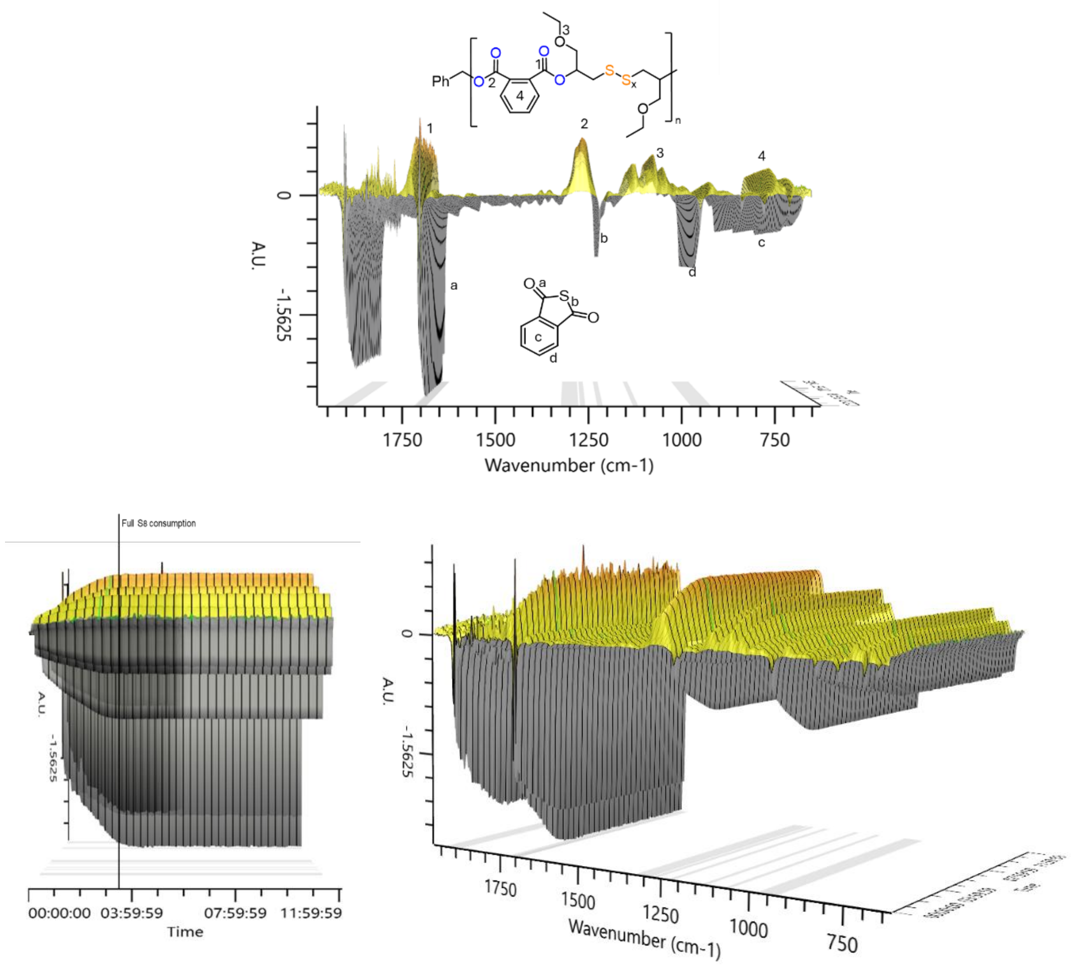

Figure S20: Different views on the overlayed *In situ* IR measurements of epoxide(EGE)-PTA-S_8 terpolymerisation at 1 eq. LiOBn: 1000 eq. EGE: 200 eq. PTA : 100 eq. S and 80ºC. Note that the baseline was set before polymerisation so that the cosumption of monomers results in negative intensity. Assignment according to reference spectra.

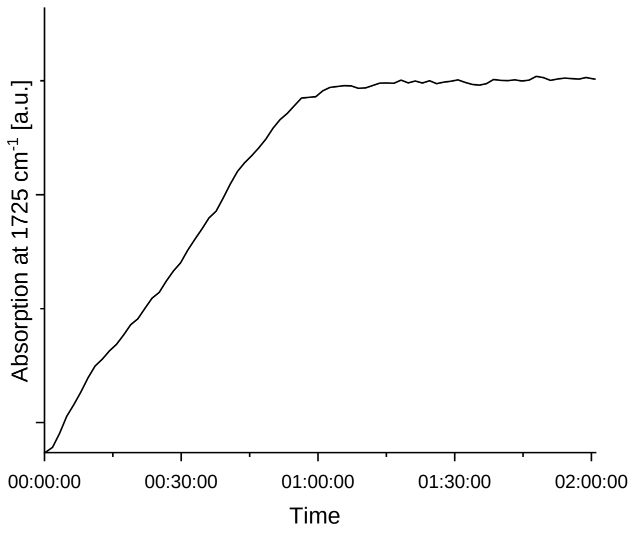

Figure S21: Increase of the polymer IR signal at 1725 cm^{-1} over time at 1 eq. LiOBn: 1000 eq. epoxide (EGE): 100 eq. PTA : 500 eg. S and 80ºC.

Section S4: Monomer scope

Monomer scoping was conducted with the same polymerisation methodology described in **Section S2;** all the reactions were run at 100°C.

Table S1: list of epoxides deployed in the monomer scope.

Run	Epoxide	Run	Epoxide
#1	BO	#11	DMO
#2	DO	#12	IBO
#3	DO 400 eq. S	#13	IBO 400 eq. S
#4	CHO	#14	VGE
#5	CHO 400 eq. S	#15	ECH
#6	CE	#16	DEO
#7	EGE	#17	ESBO
#8	PGE	#18	PO-NTA
#9	SO	#19	VGE-NTA
#10	SO 400 eq. S	#20	CHO-NTA

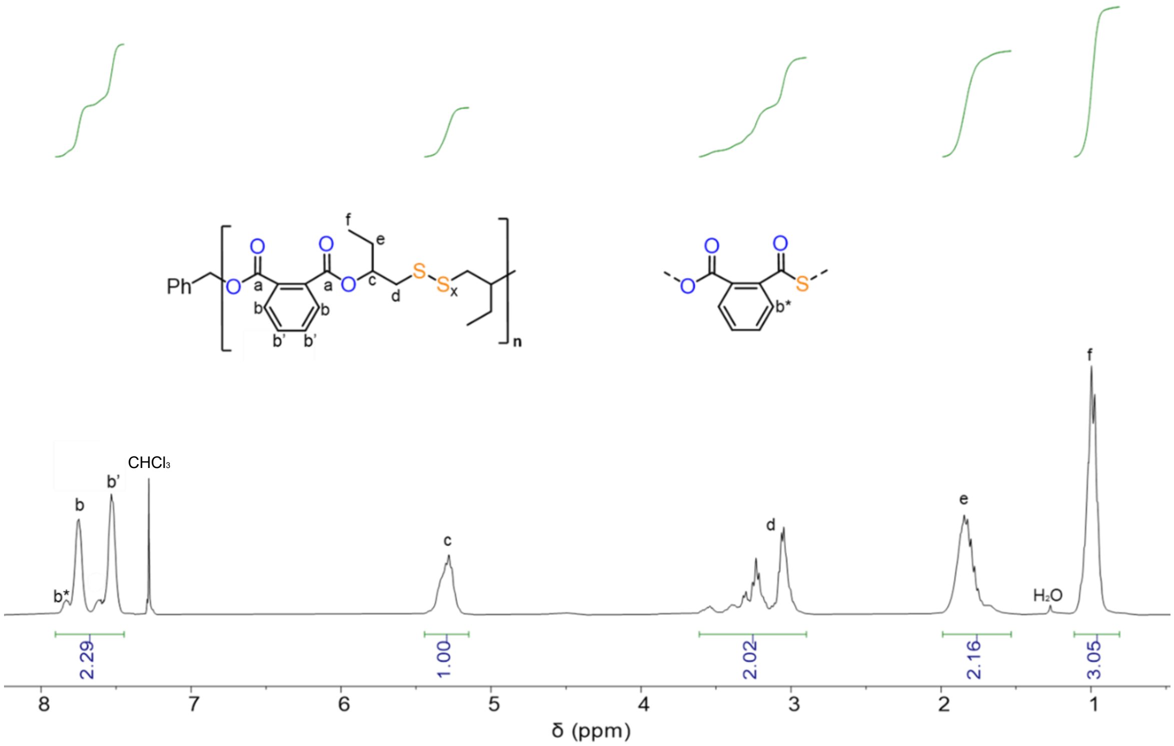

Figure S22: ^{1}H-NMR spectrum (600 MHz, $CDCl_3$, 25°C) of the precipitated polymer corresponding to ESI table 1 run #1.

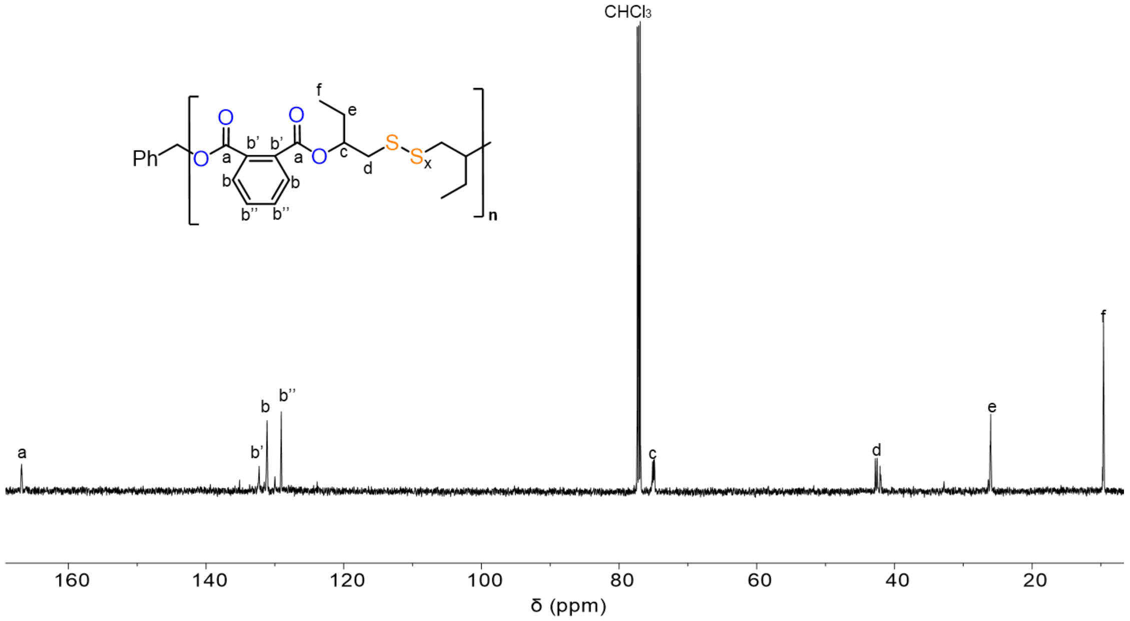

Figure S23: ^{13}C-NMR spectrum (151 MHz, $CDCl_3$, 25°C) of the precipitated polymer corresponding to ESI table 1 run #1.

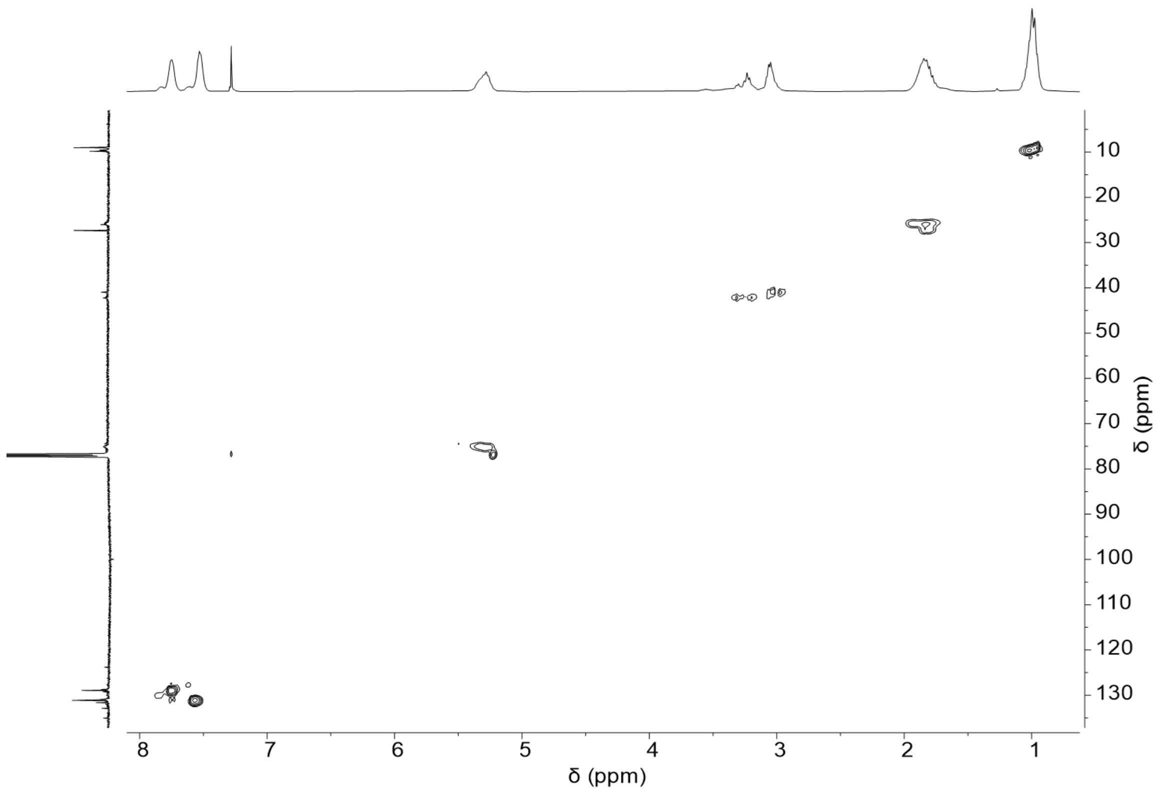

Figure S24: ^{1}H-^{13}C HSQC NMR spectrum ($CDCl_3$, 25°C) of the precipitated polymer corresponding to ESI table 1 run #1.

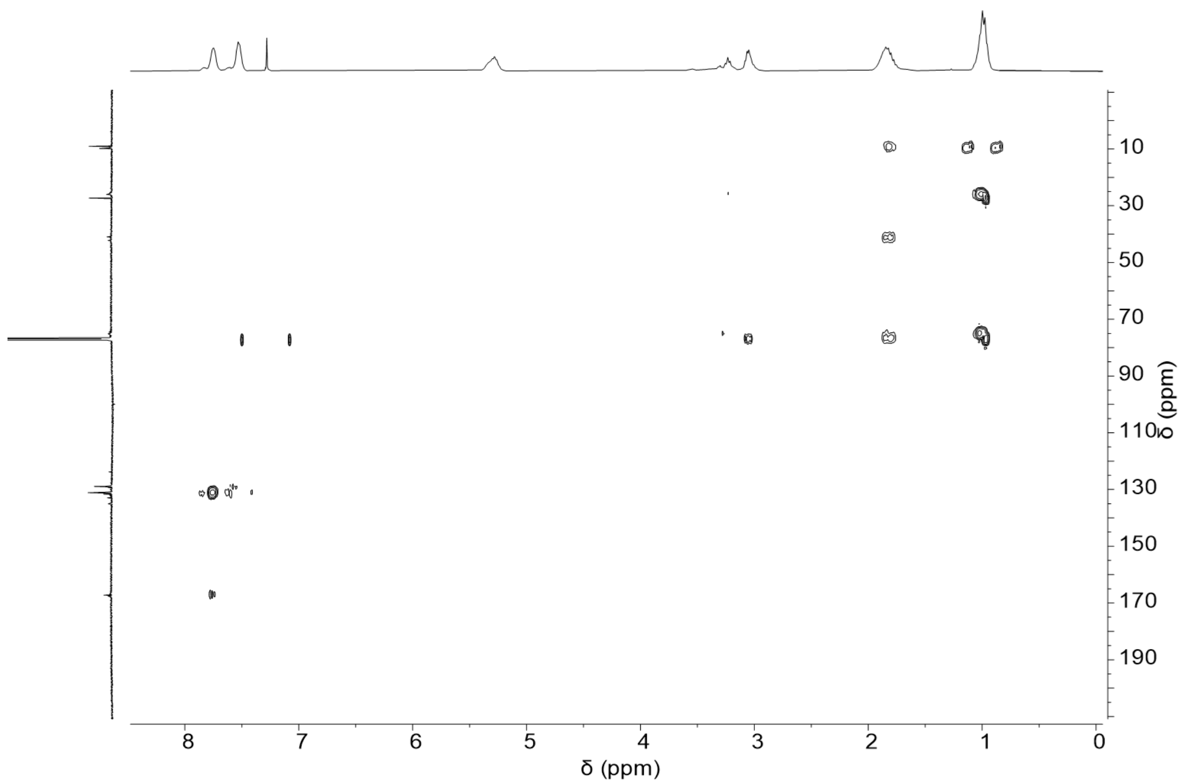

Figure S25: ^{1}H-^{13}C HMBC NMR spectrum ($CDCl_3$, 25°C) of the precipitated polymer corresponding to table run #1.

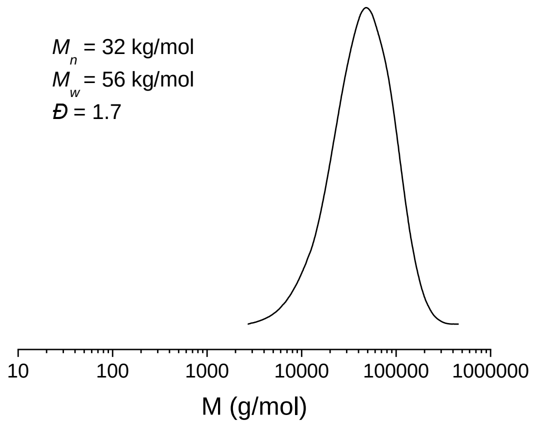

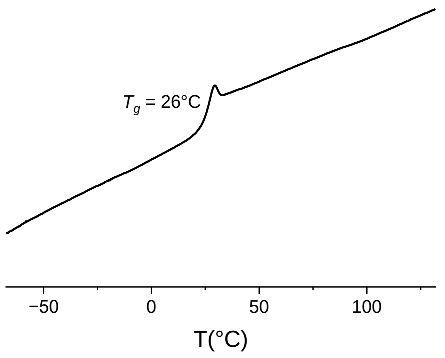

Figure S26: (left) GPC curve and (right) DSC 2nd heating curve of the precipitated polymer corresponding to ESI table 1 run #1.

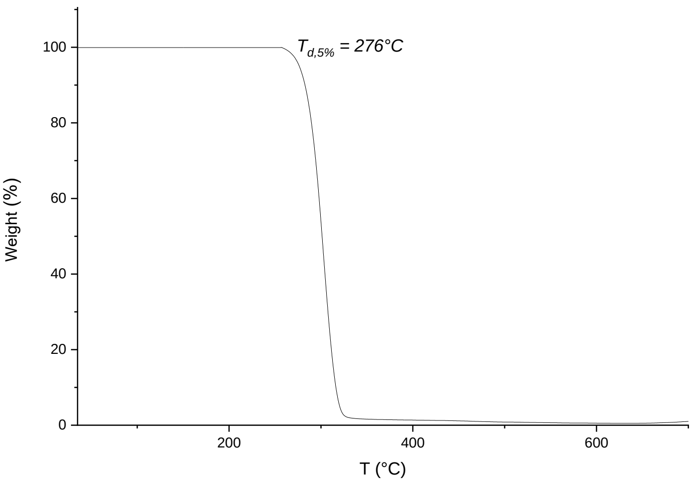

Figure S27: TGA data of the polymer corresponding to ESI table 1 run # 1.

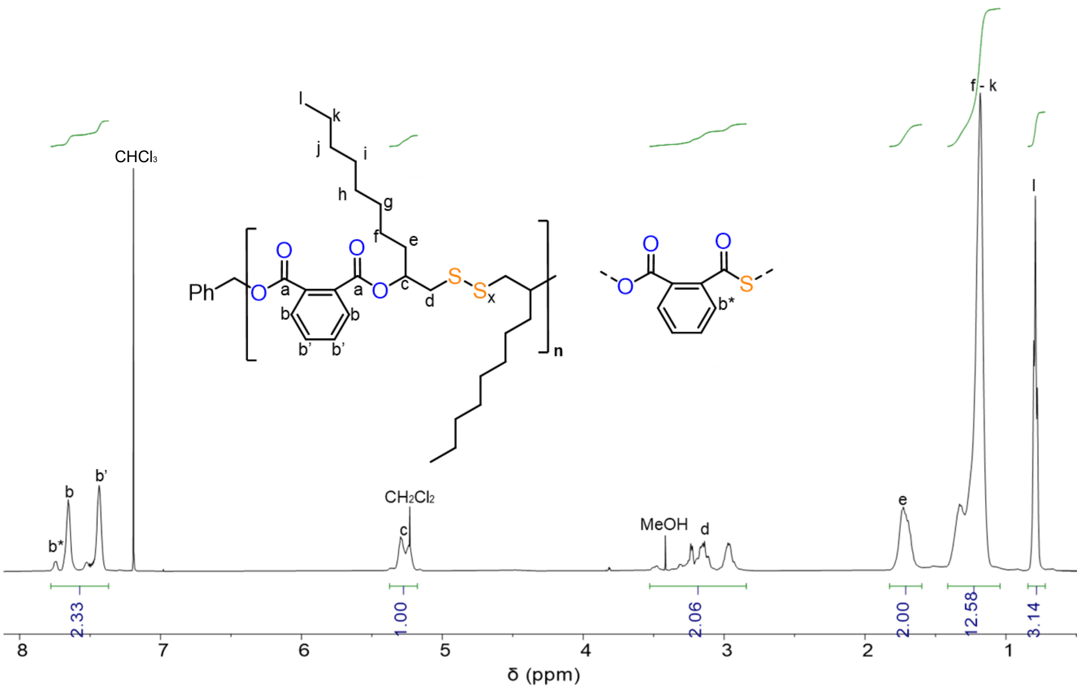

Figure S28: ^{1}H-NMR spectrum (600 MHz, $CDCl_3$, 25°C) of the precipitated polymer corresponding to ESI table 1 run #2.

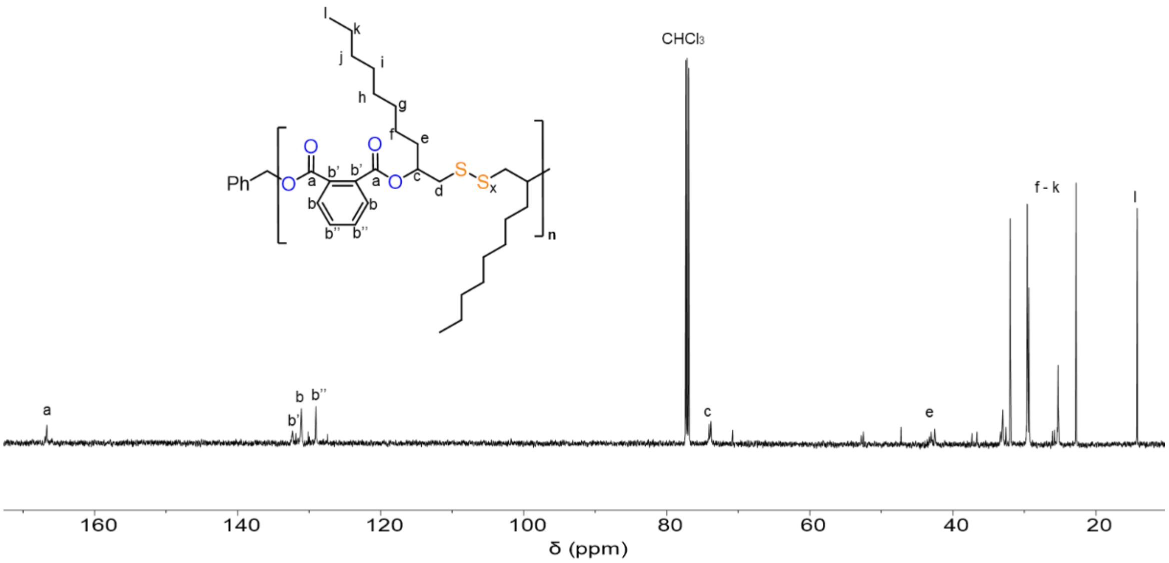

Figure S29: ^{13}C-NMR spectrum (151 MHz, $CDCl_3$, 25°C) of the precipitated polymer corresponding to ESI table 1 run #2.

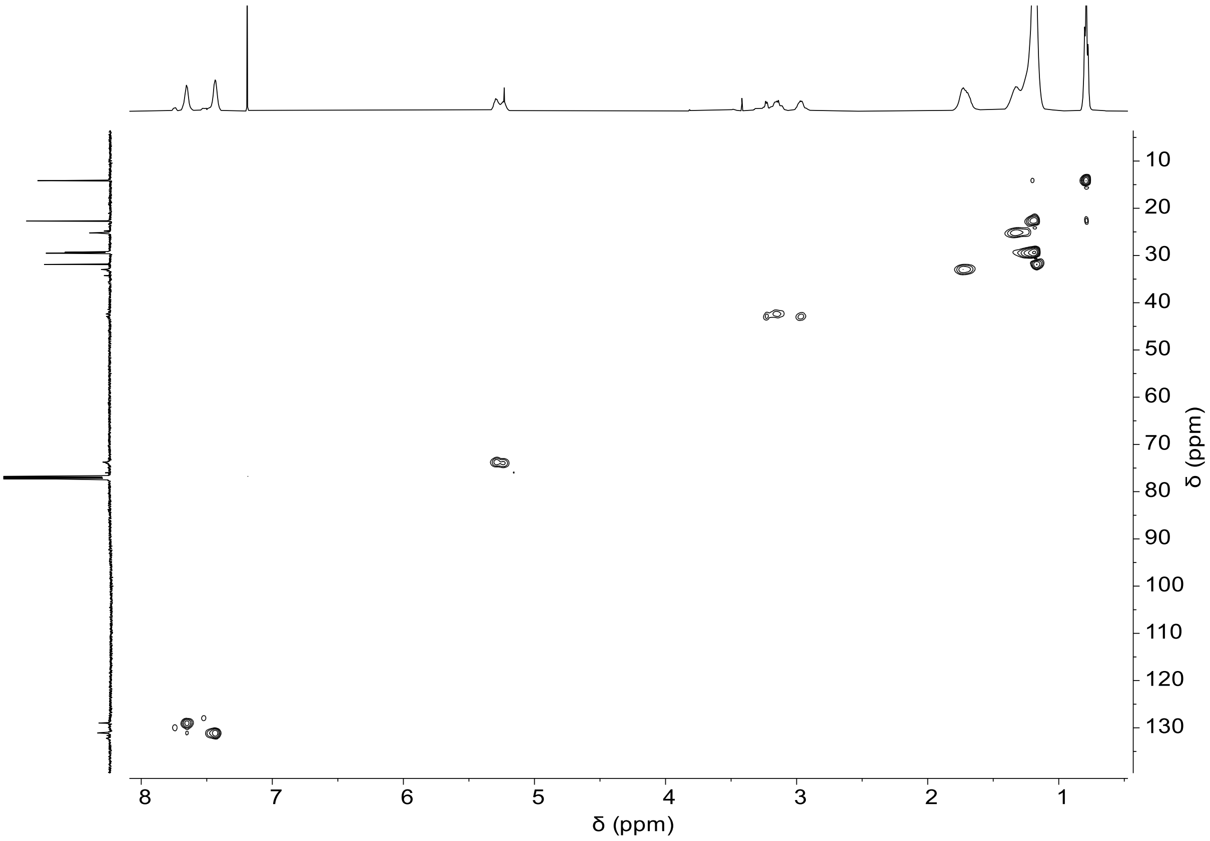

Figure S30: ^{1}H-^{13}C HSQC NMR spectrum ($CDCl_3$, 25°C) of the precipitated polymer corresponding to ESI table 1 run #2.

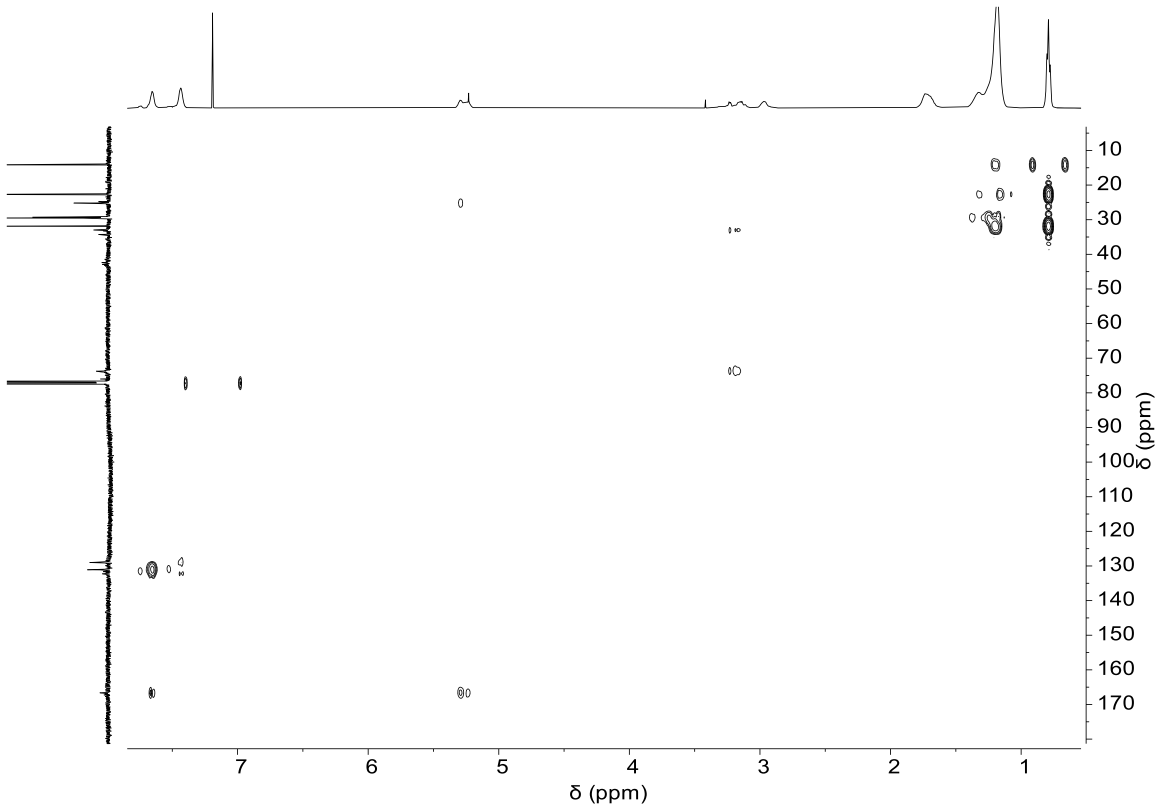

Figure S31: ^{1}H-^{13}C HMBC NMR spectrum ($CDCl_3$, 25°C) of the precipitated polymer corresponding to ESI table 1 run #2.

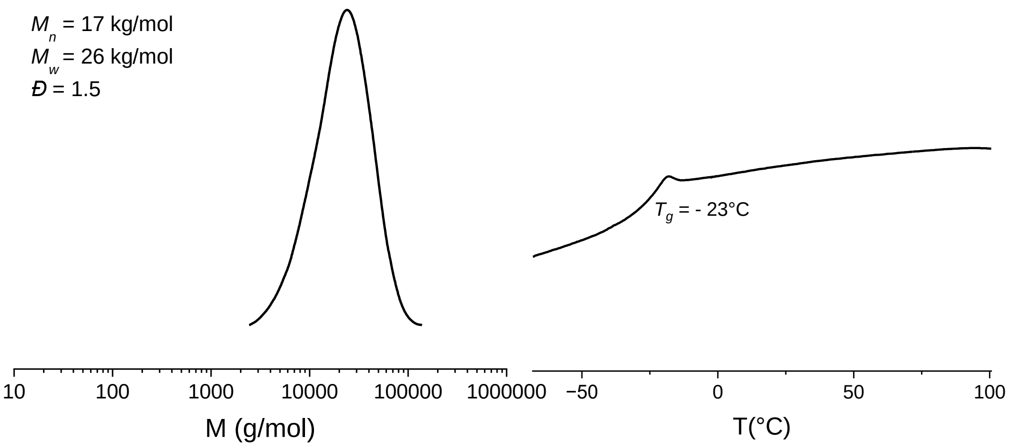

Figure S32: (left) GPC curve and (right) DSC 2nd heating curve of the precipitated polymer corresponding to ESI table 1 run #2.

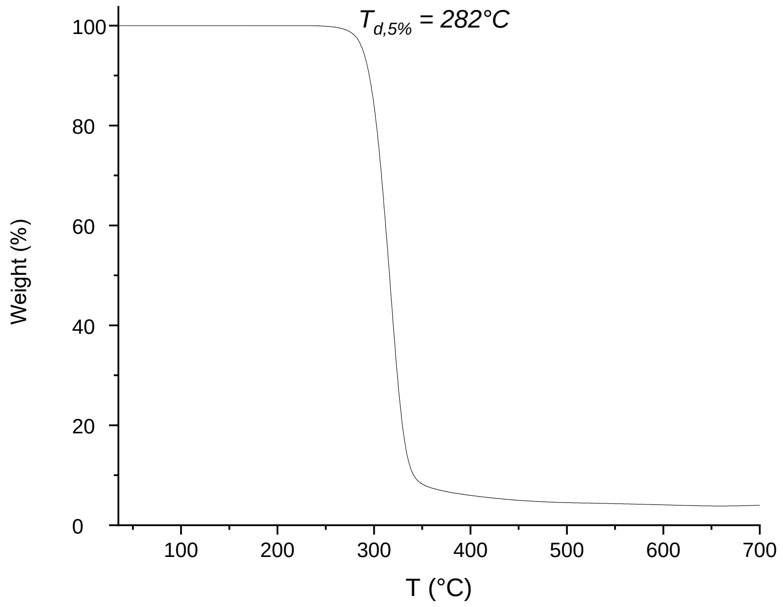

Figure S33: TGA data of the polymer corresponding to ESI table 1 run #2.

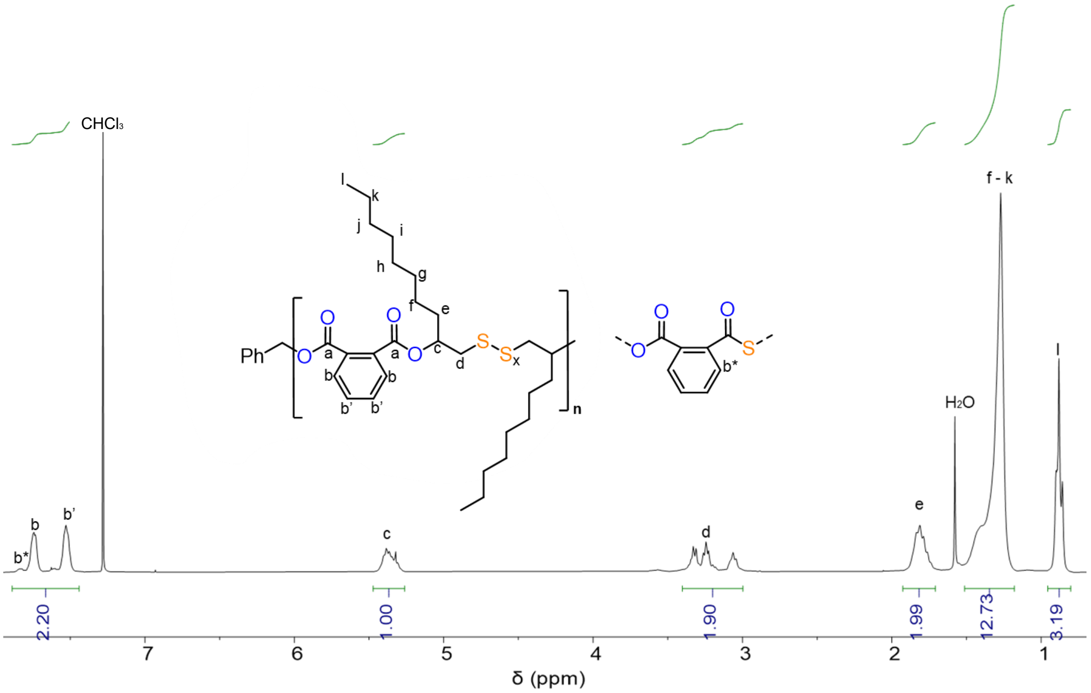

Figure S34: ^{1}H-NMR spectrum (600 MHz, $CDCl_3$, 25°C) of the precipitated polymer corresponding to ESI table 1 run #3.

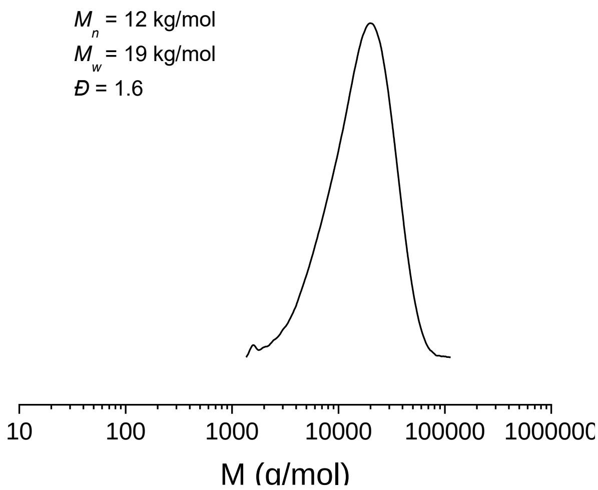

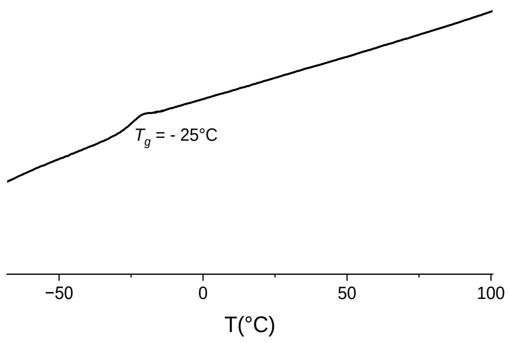

Figure S35: (left) GPC curve and (right) DSC 2nd heating curve of the precipitated polymer corresponding to ESI table 1 run #3.

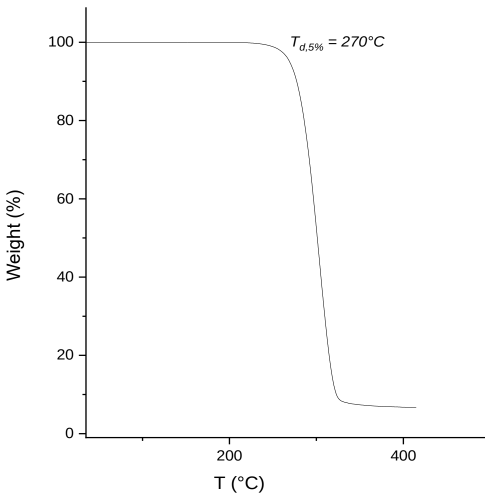

Figure S36: TGA data of the polymer corresponding to ESI table 1 run #3.

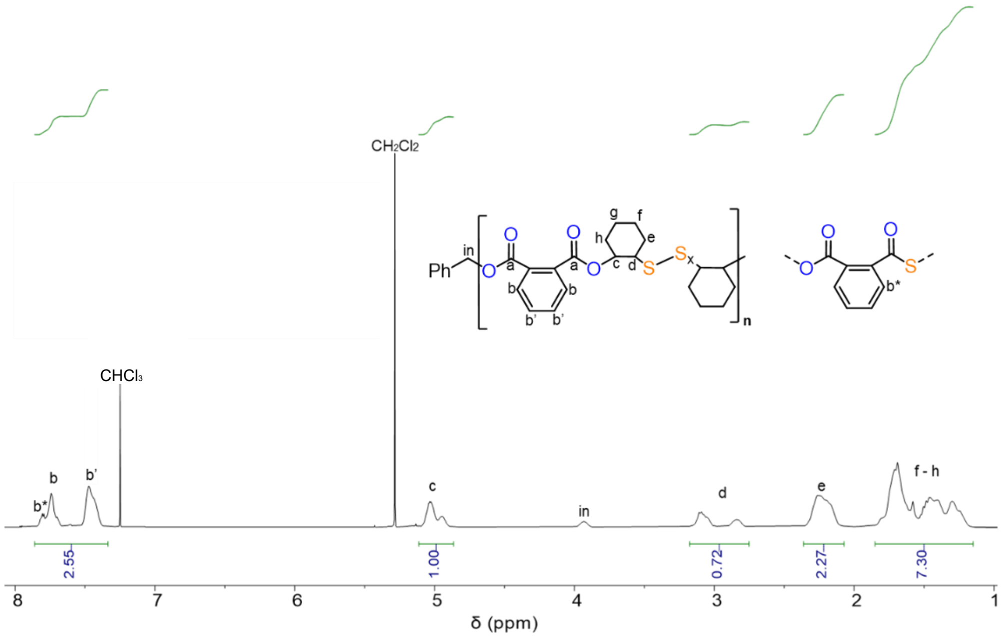

Figure S37: ^{1}H-NMR spectrum (600 MHz, $CDCl_3$, 25°C) of the precipitated polymer corresponding to ESI table 1 run #4.

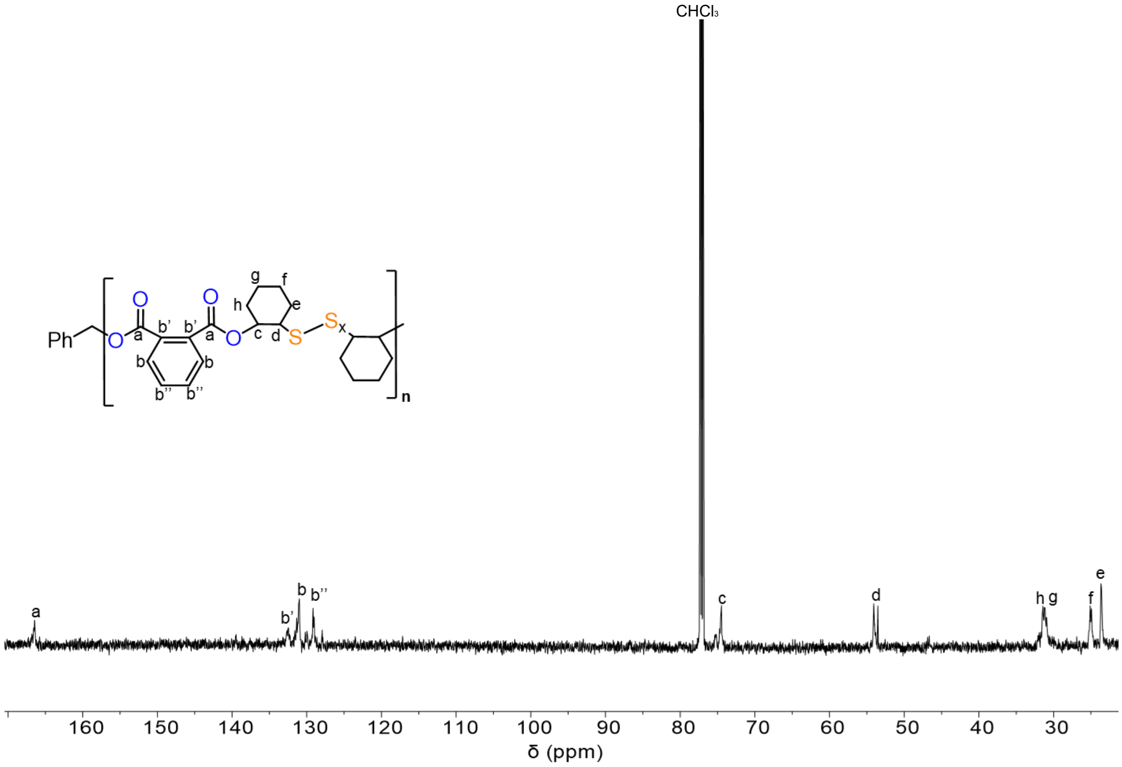

Figure S38: ^{13}C-NMR spectrum (151 MHz, $CDCl_3$, 25°C) of the precipitated polymer corresponding to ESI table 1 run #4.

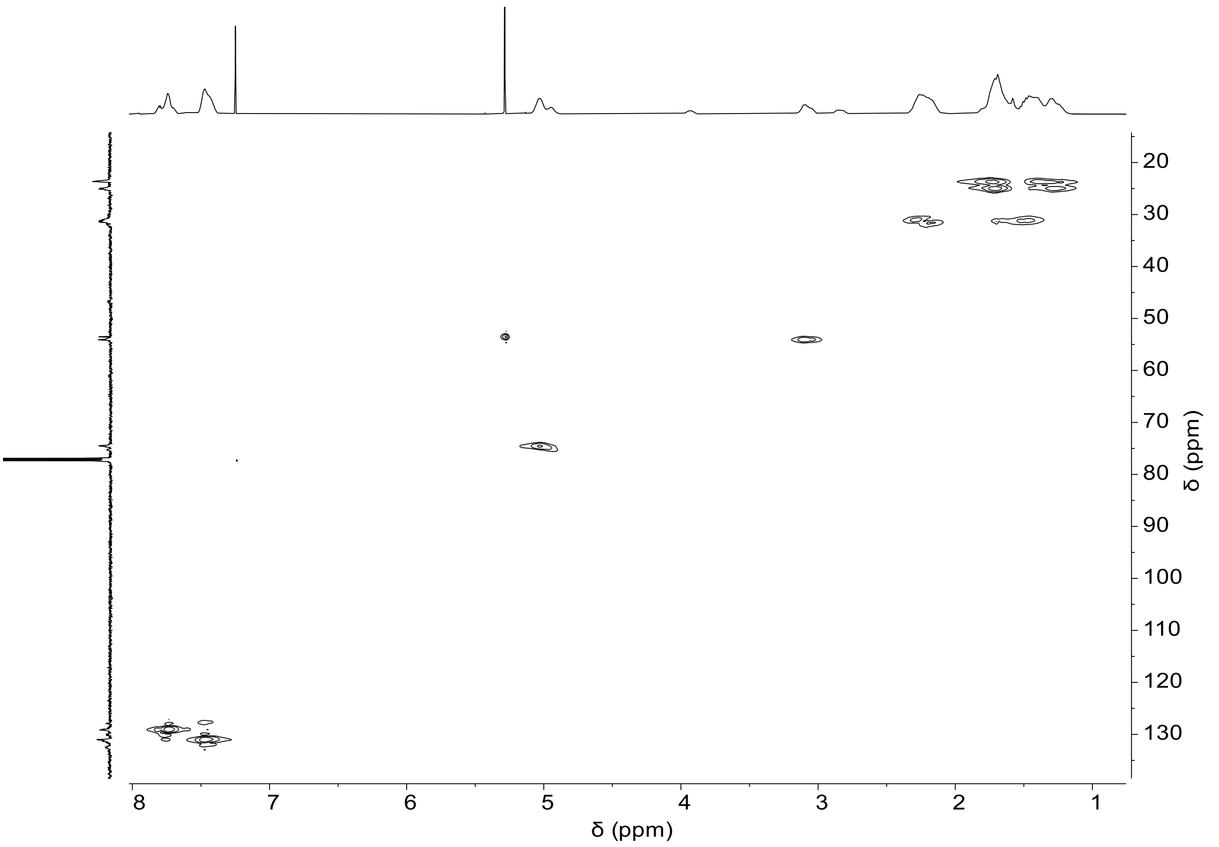

Figure S39: ^{1}H-^{13}C HSQC NMR spectrum ($CDCl_3$, 25°C) of the precipitated polymer corresponding to ESI table 1 run #4.

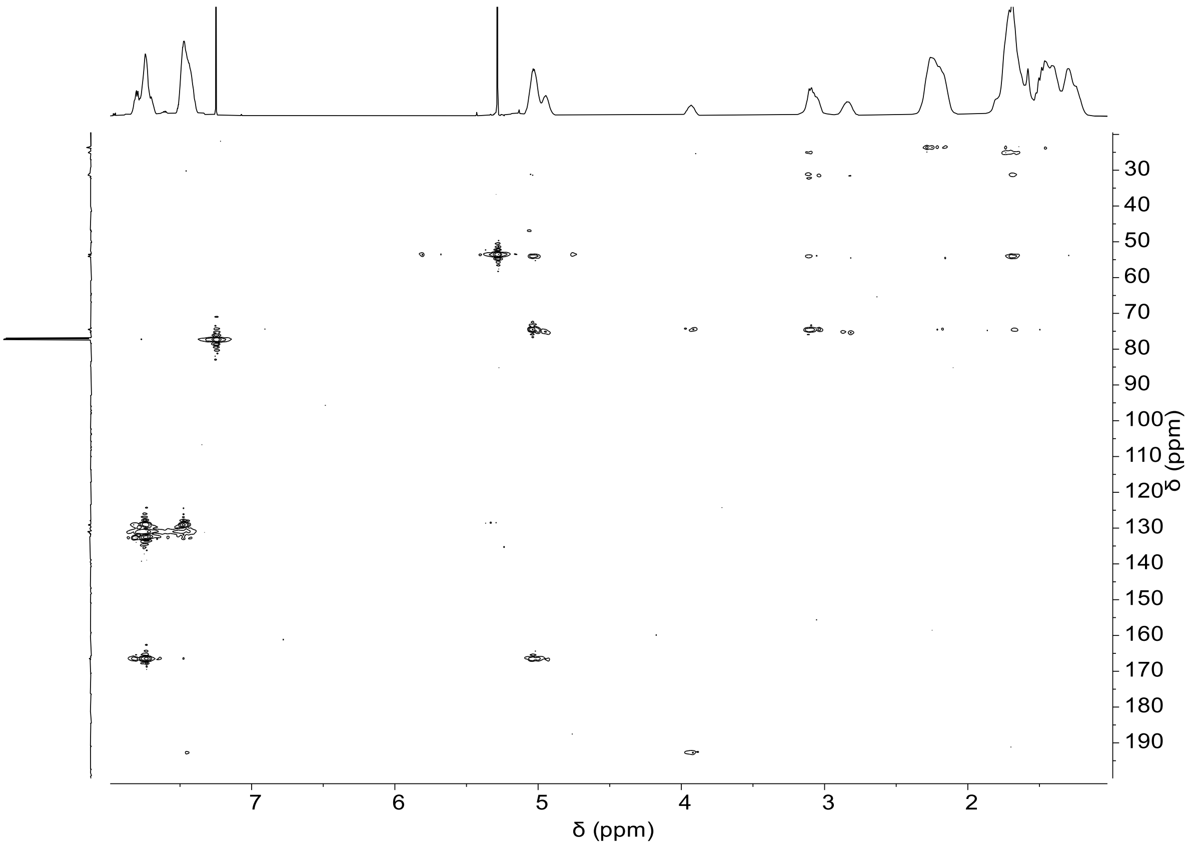

Figure S40: ^{1}H-^{13}C HMBC NMR spectrum ($CDCl_3$, 25°C) of the precipitated polymer corresponding to ESI table 1 run #4.

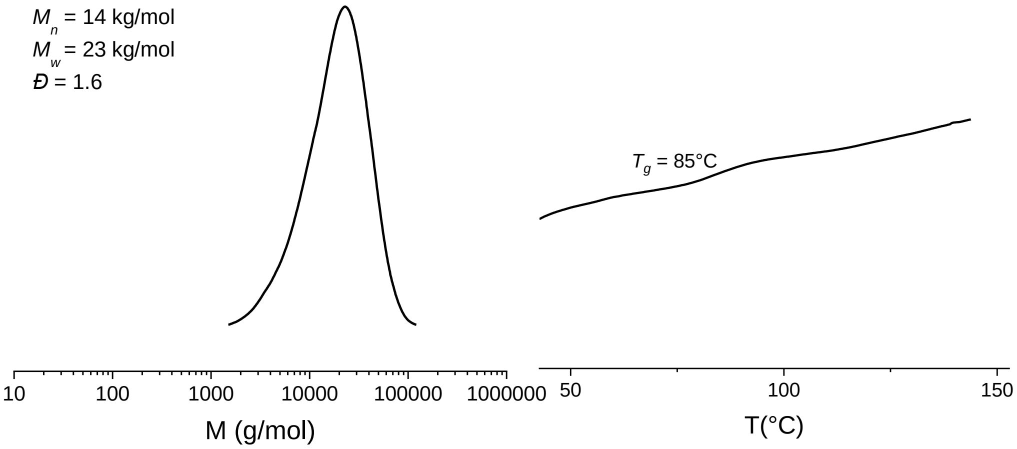

Figure S41: (left) GPC curve and (right) DSC 2nd heating curve of the precipitated polymer corresponding to ESI table 1 run #4.

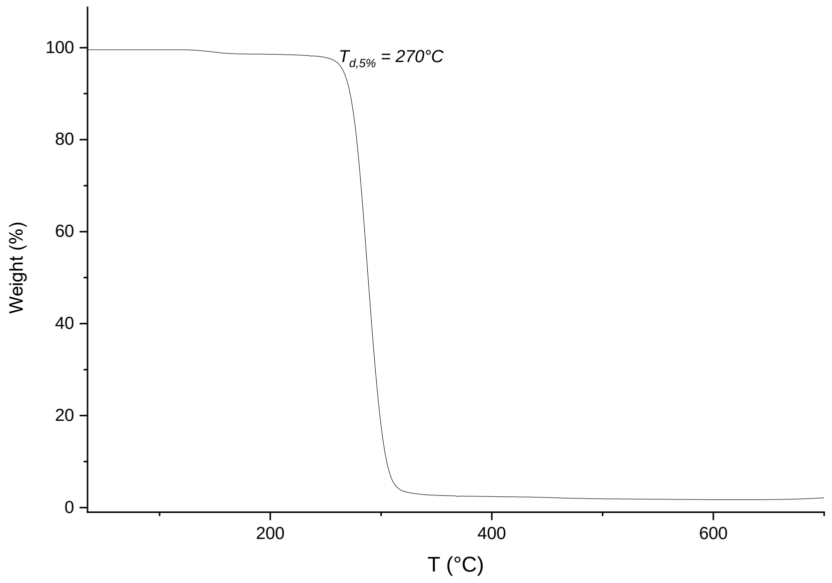

Figure S42: TGA data of the polymer corresponding to ESI table 1 run #4.

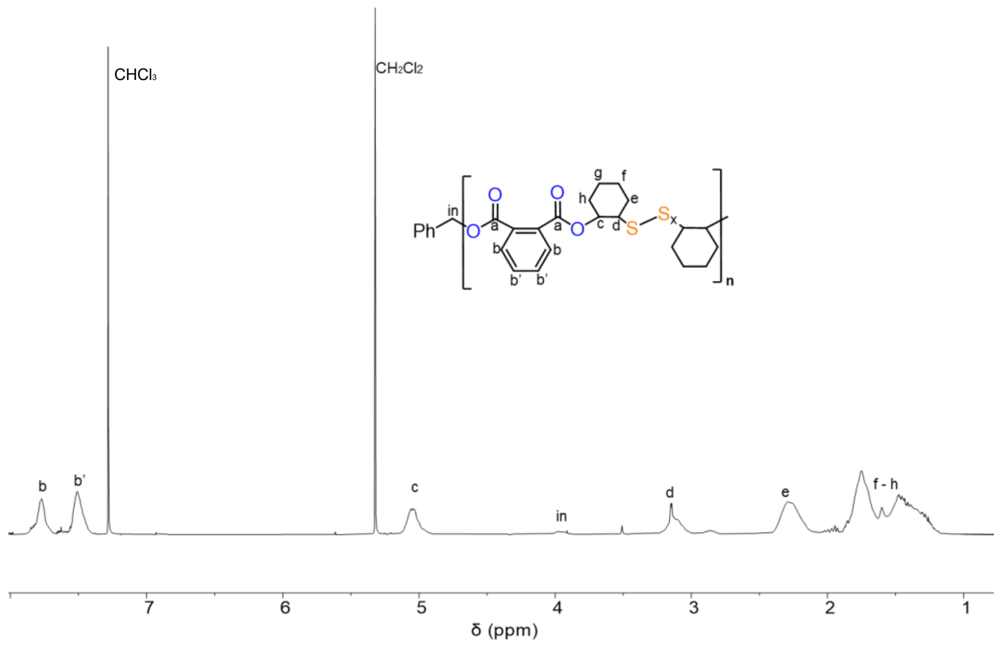

Figure S43: ^{1}H-NMR spectrum (600 MHz, $CDCl_3$, 25°C) of the precipitated polymer corresponding to ESI table 1 run #5.

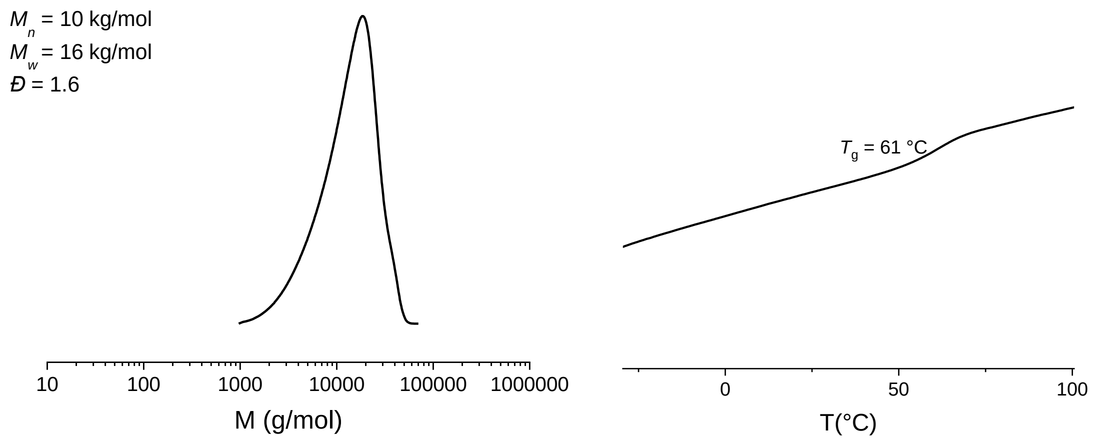

Figure S44: (left) GPC curve and (right) DSC 2nd heating curve of the precipitated polymer corresponding to ESI table 1 run #5.

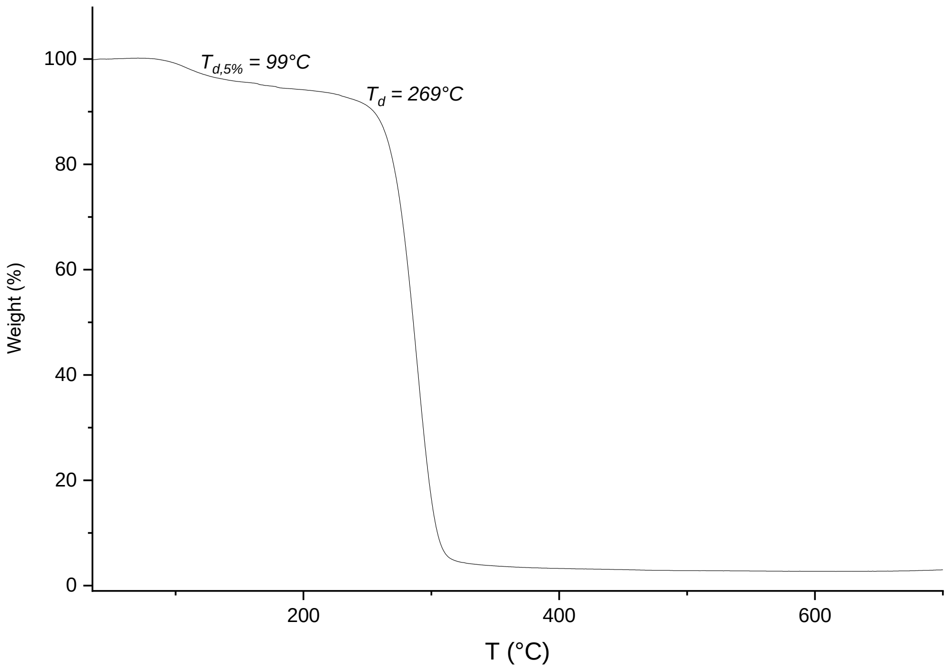

Figure S45: TGA data of the polymer corresponding to ESI table 1 run #5.

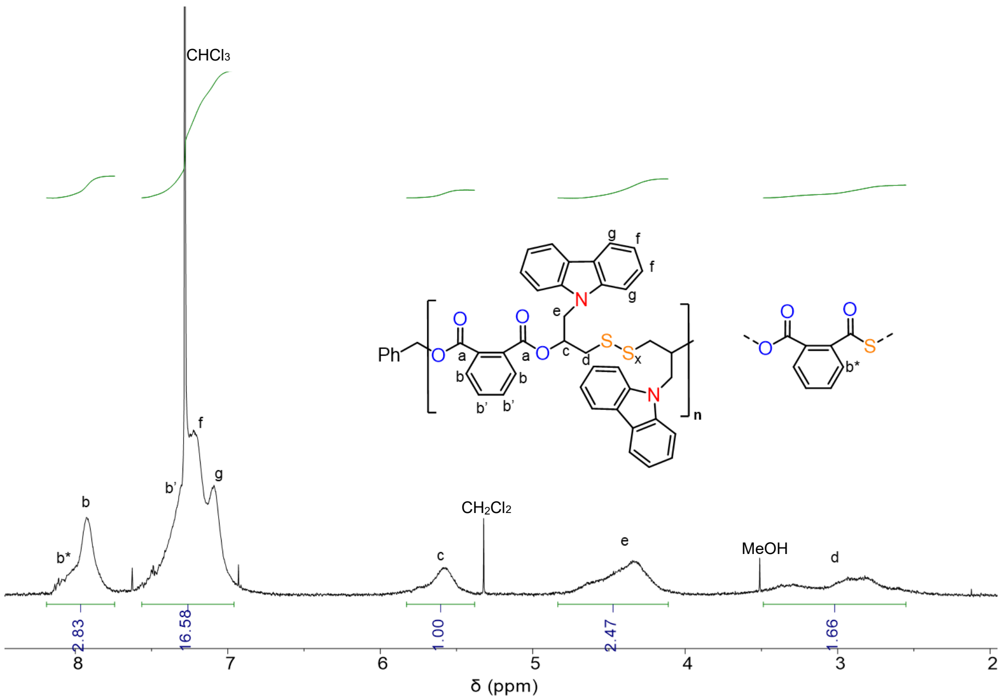

Figure S46: ^{1}H-NMR spectrum (600 MHz, $CDCl_3$, 25°C) of the precipitated polymer corresponding to ESI table 1 run #6.

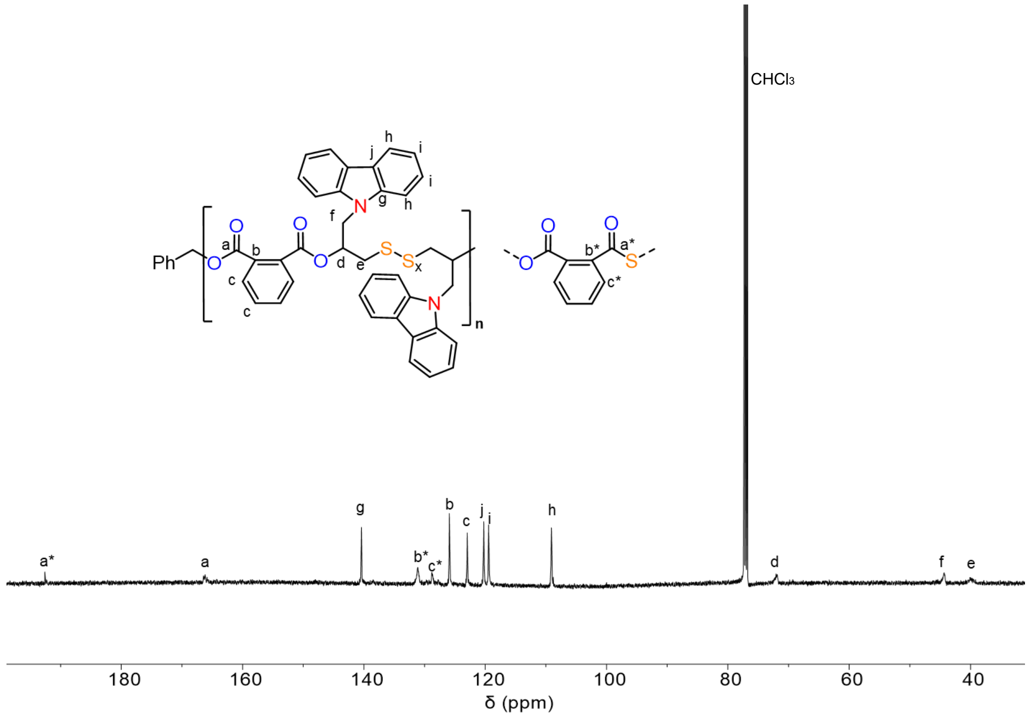

Figure S47: ^{13}C-NMR spectrum (151 MHz, $CDCl_3$, 25°C) of the precipitated polymer corresponding to ESI table 1 run #6.

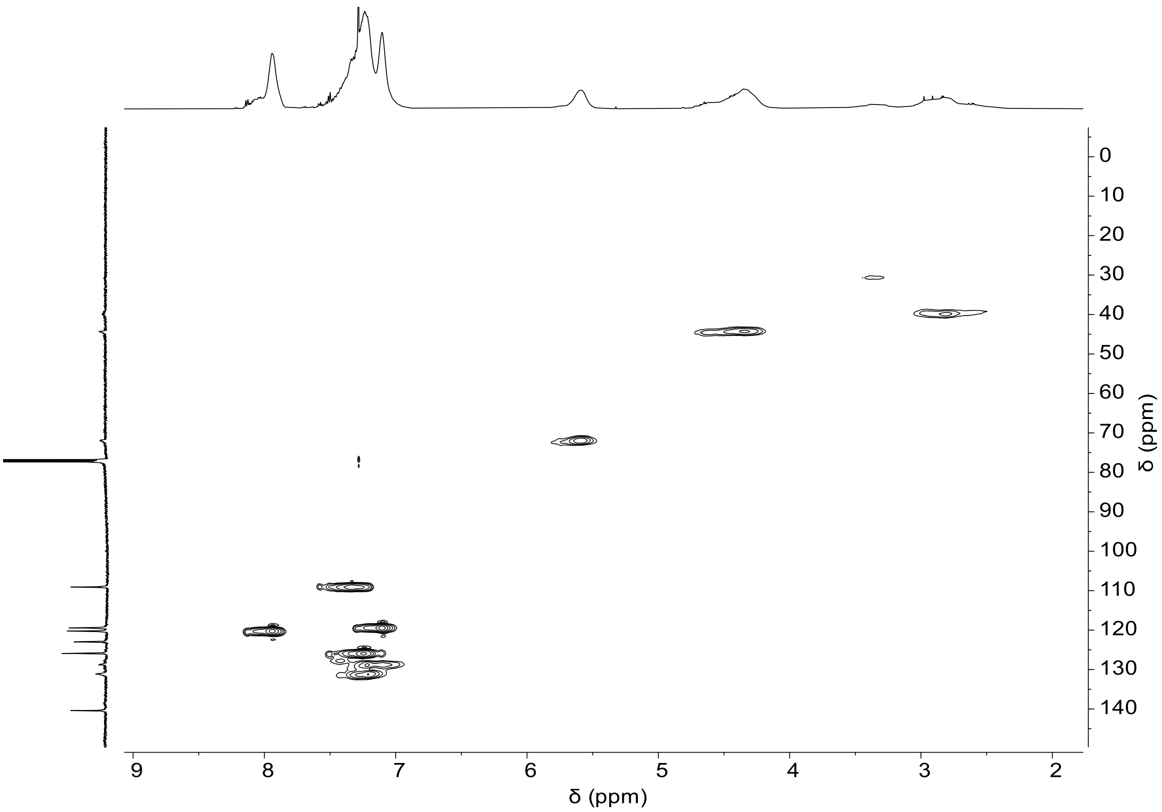

Figure S48: ^{1}H-^{13}C HSQC NMR spectrum ($CDCl_3$, 25°C) of the precipitated polymer corresponding to ESI table 1 run #6.

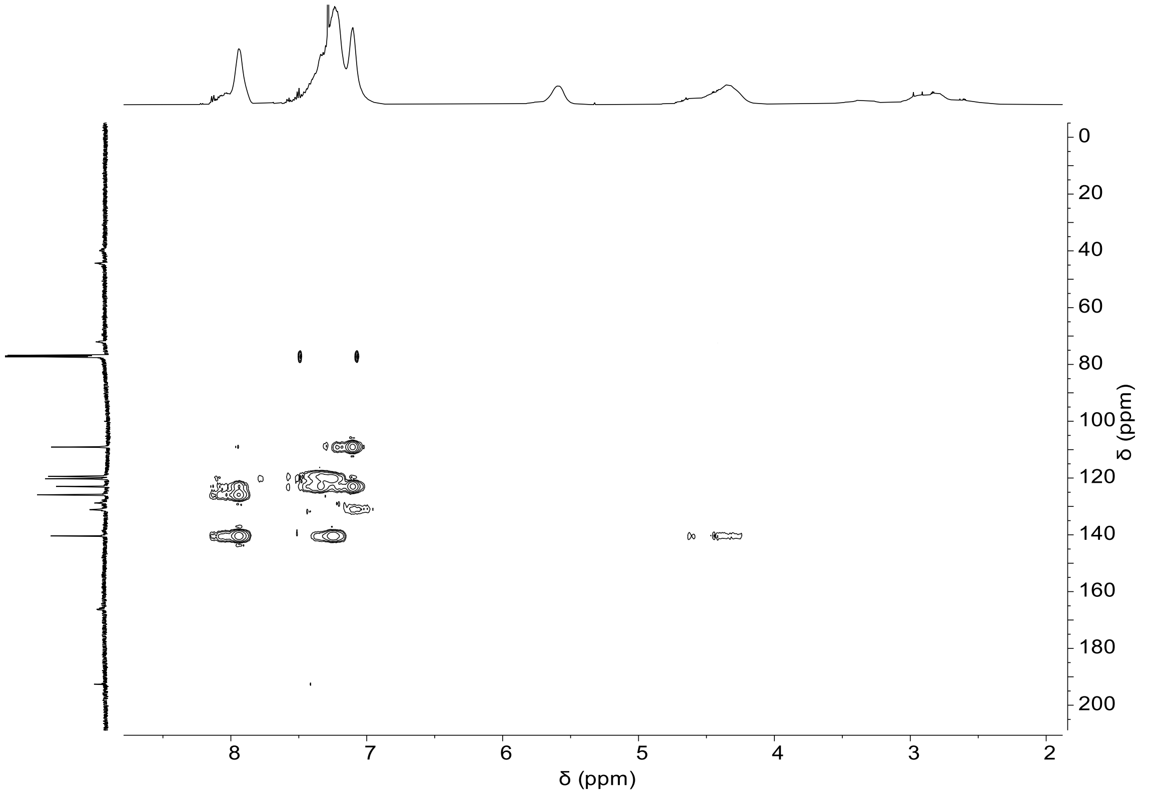

Figure S49: ^{1}H-^{13}C HMBC NMR spectrum ($CDCl_3$, 25°C) of the precipitated polymer corresponding to ESI table 1 run #6.

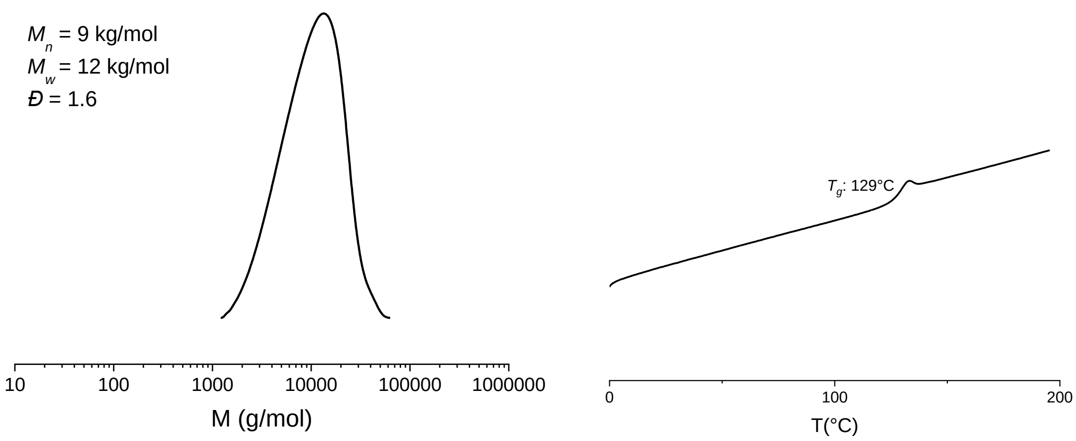

Figure S50: (left) GPC curve and (right) DSC 2nd heating curve of the precipitated polymer corresponding to ESI table 1 run #6.

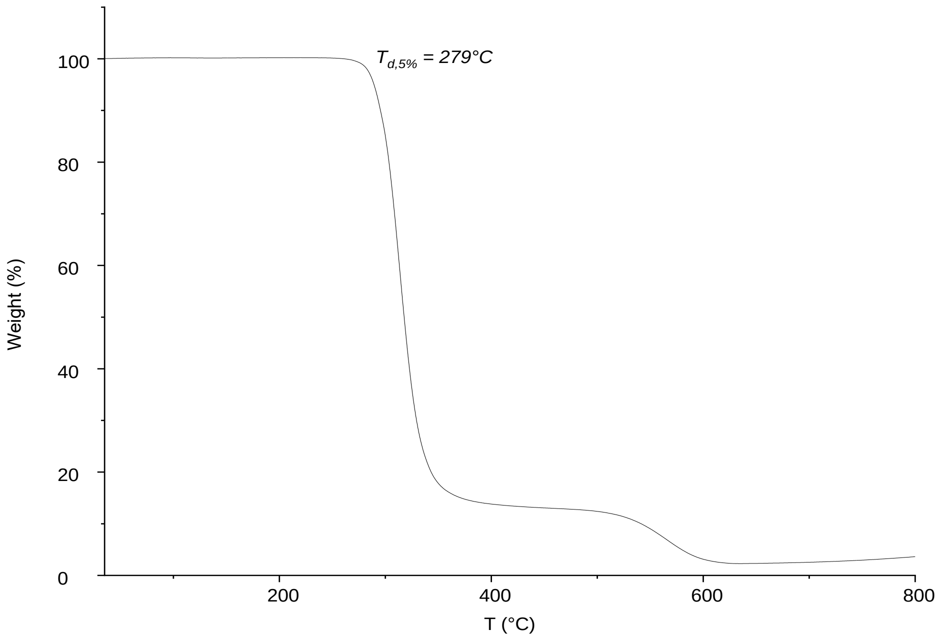

Figure S51: TGA data of the polymer corresponding to ESI table 1 run #6.

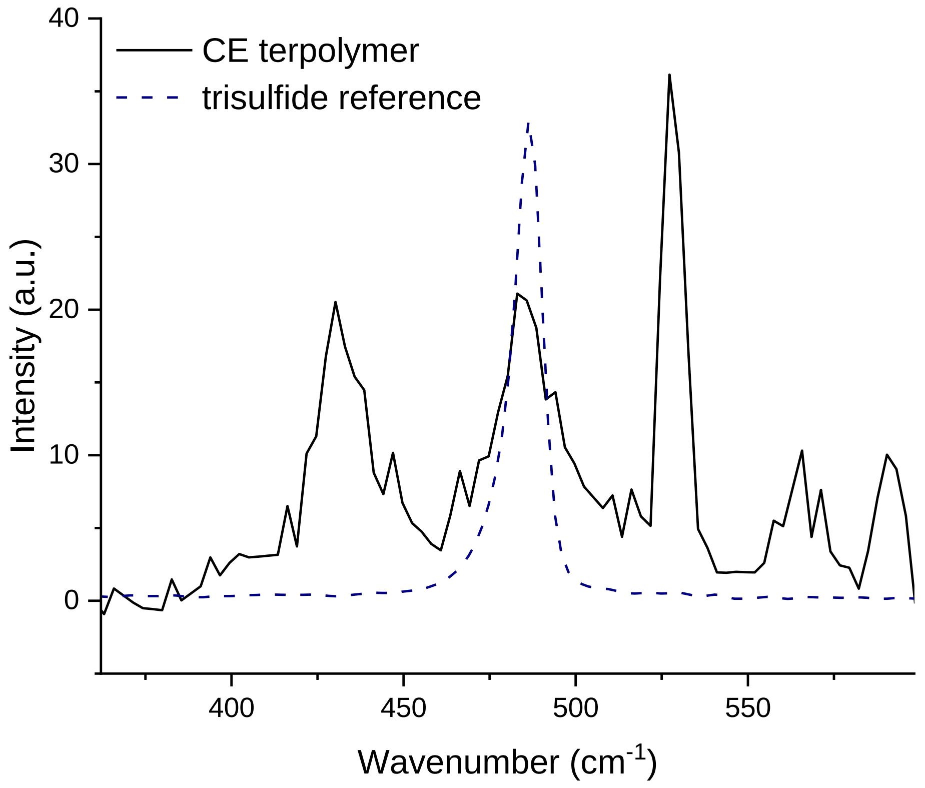

Figure S52: Zoom into the C-S_x-C stretching vibrations of the Raman spectra of the polymer corresponding to ESI table 1 run #6, compared with dimethyl trisulfide reference compound.

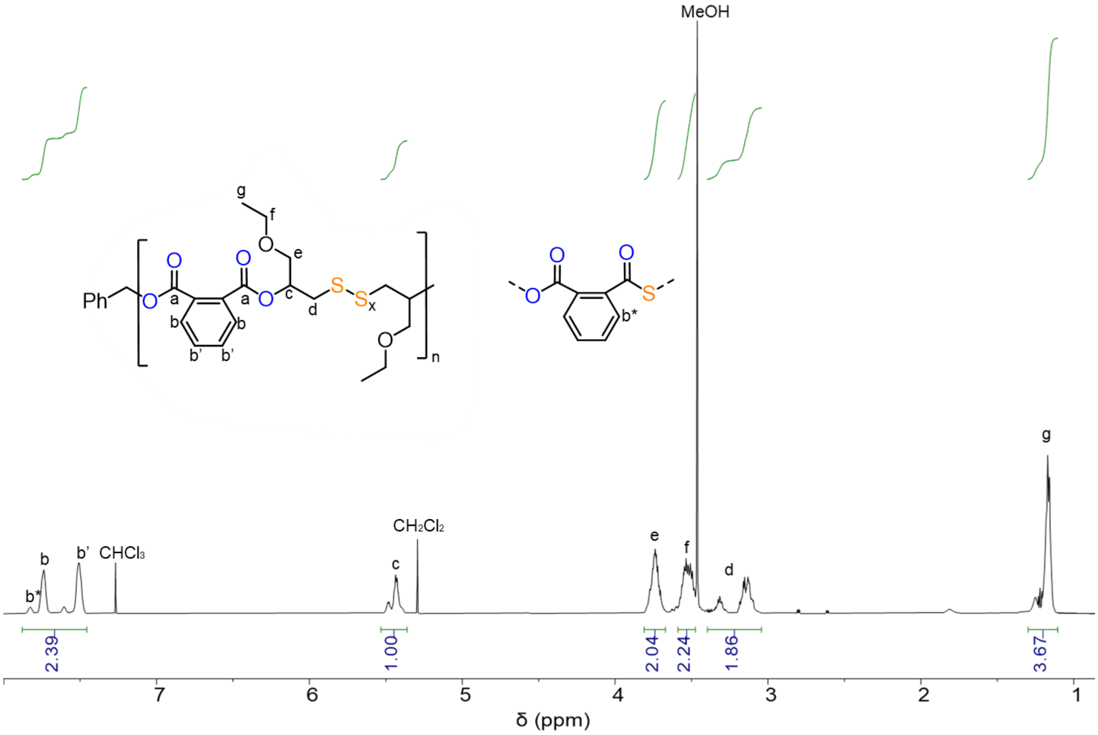

Figure S53: ^{1}H-NMR spectrum (600 MHz, $CDCl_3$, 25°C) of the precipitated polymer corresponding to ESI table 1 run #7.

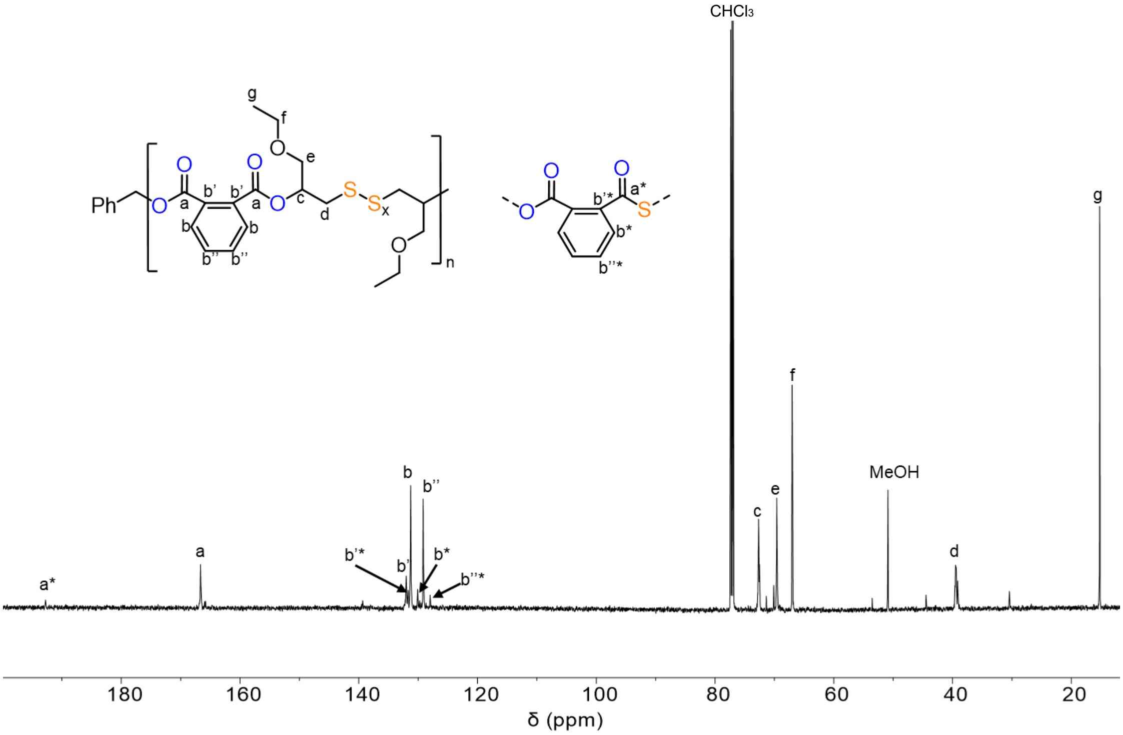

Figure S54: ^{13}C-NMR spectrum (151 MHz, $CDCl_3$, 25°C) of the precipitated polymer corresponding to ESI table 1 run #7.

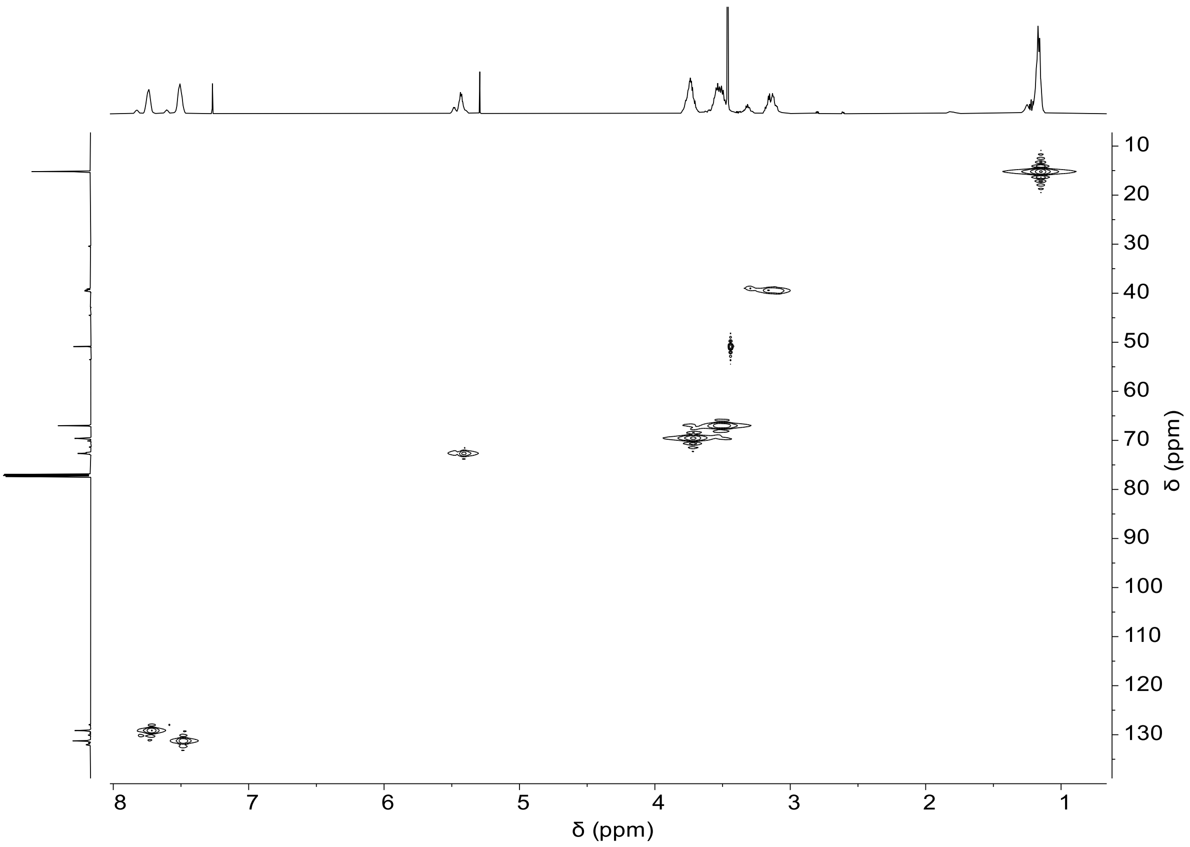

Figure S55: ^{1}H-^{13}C HSQC NMR spectrum ($CDCl_3$, 25°C) of the precipitated polymer corresponding to ESI table 1 run #7.

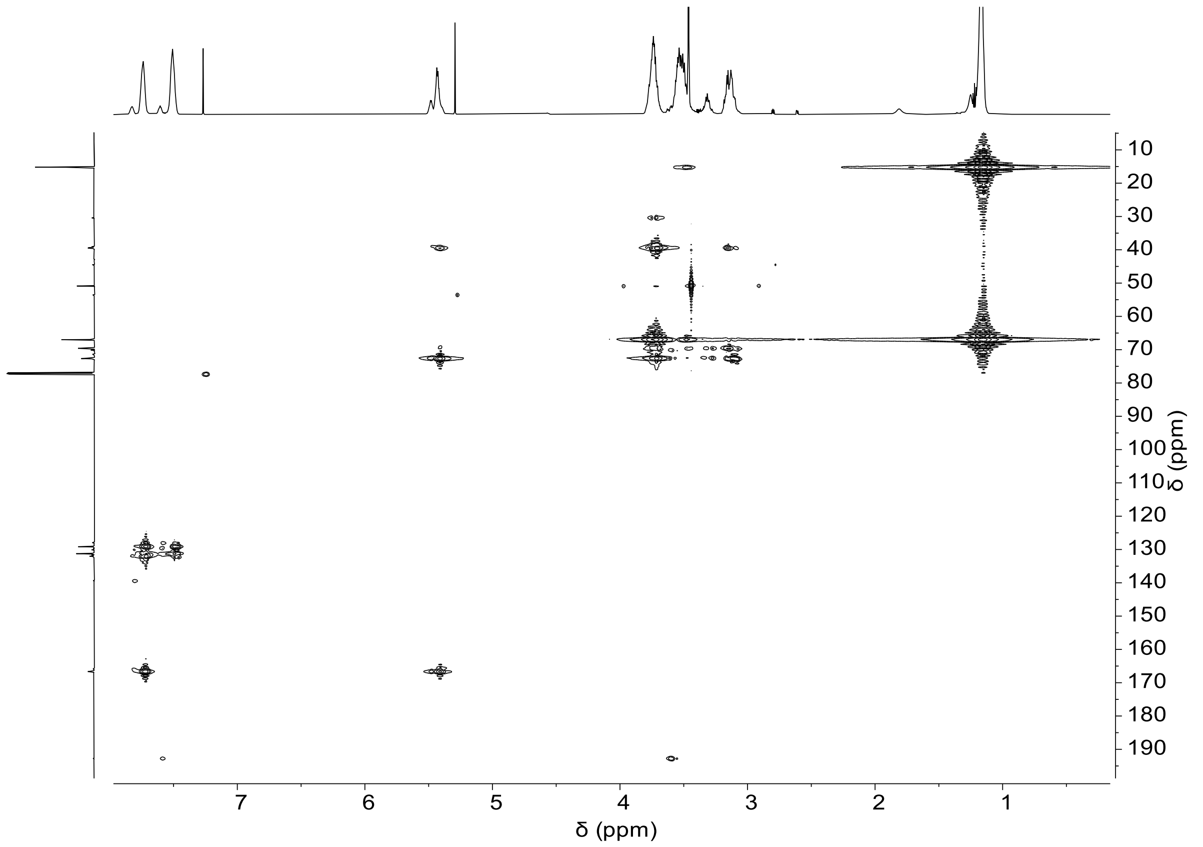

Figure S56: ^{1}H-^{13}C HMBC NMR spectrum ($CDCl_3$, 25°C) of the precipitated polymer corresponding to ESI table 1 run #7.

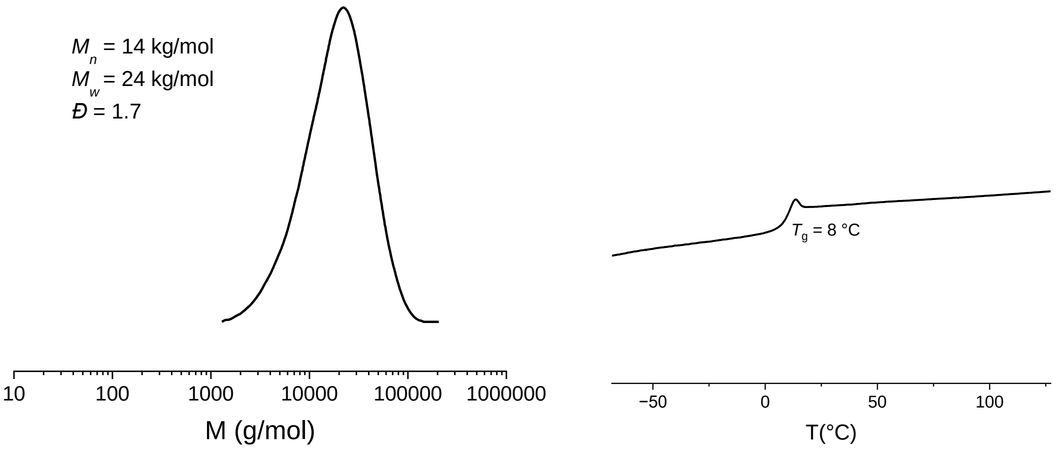

Figure S57: (left) GPC curve and (right) DSC 2nd heating curve of the precipitated polymer corresponding to ESI table 1 run #7.

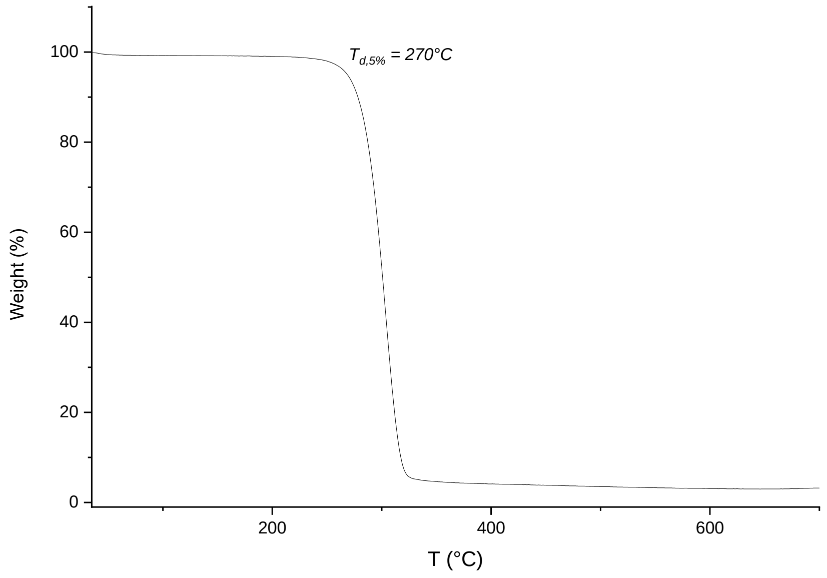

Figure S58: TGA data of the polymer corresponding to ESI table 1 run #7.

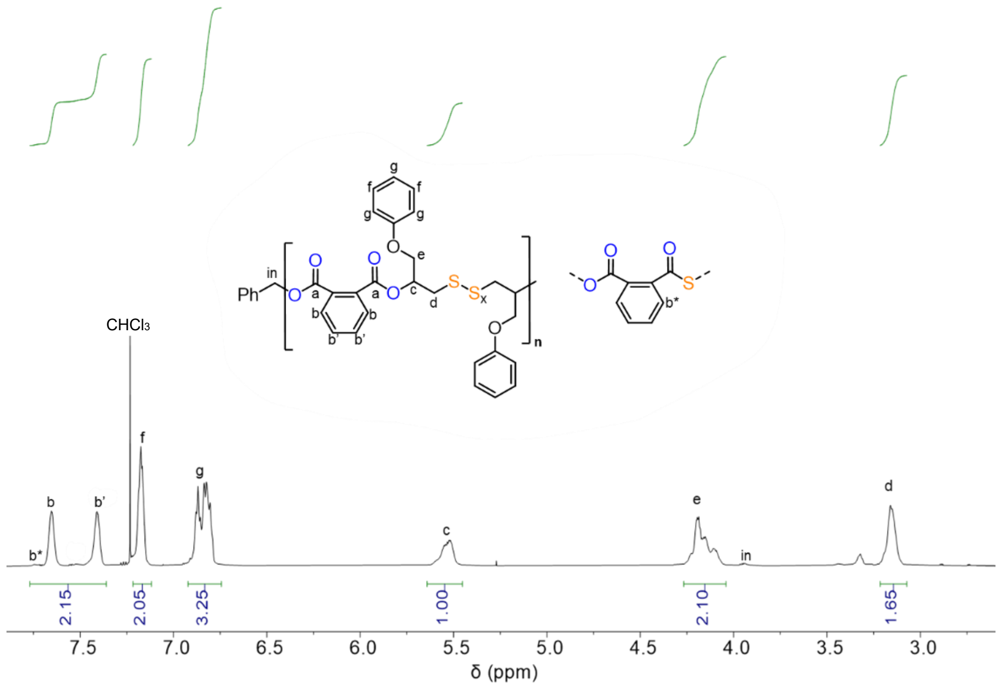

Figure S59: ^{1}H-NMR spectrum (600 MHz, $CDCl_3$, 25°C) of the precipitated polymer corresponding to ESI table 1 run #8.

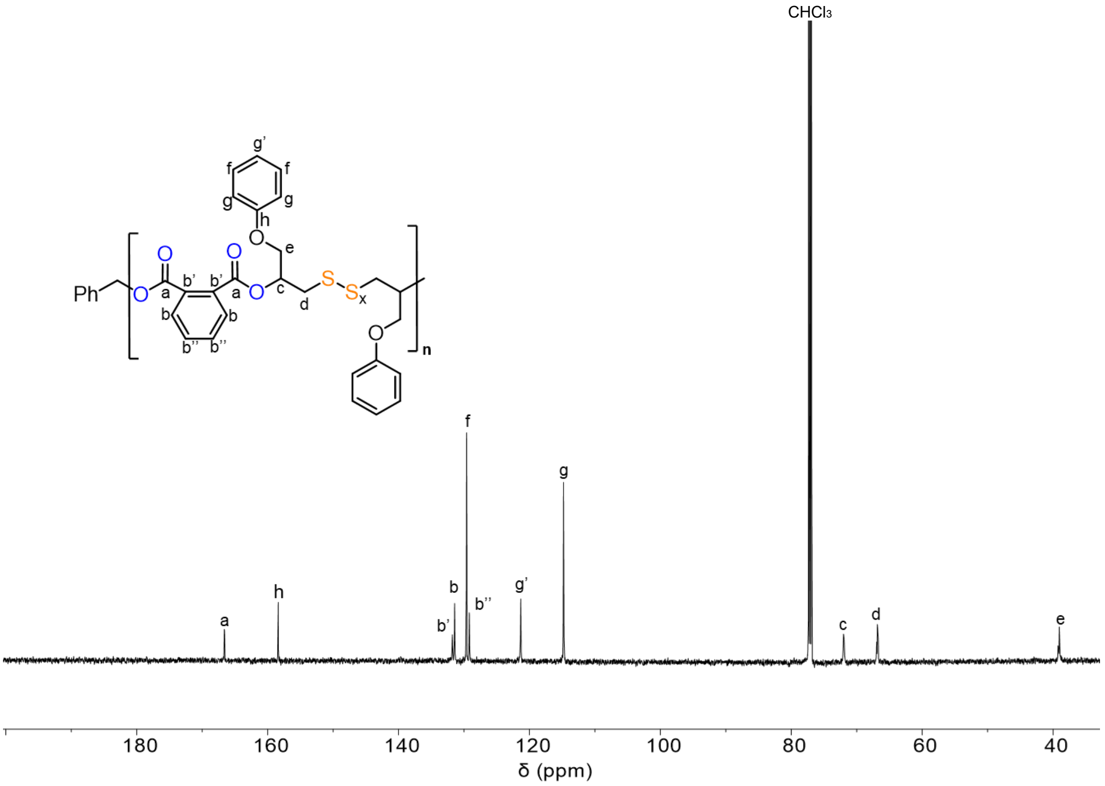

Figure S60: ^{13}C-NMR spectrum (151 MHz, $CDCl_3$, 25°C) of the precipitated polymer corresponding to ESI table 1 run #8.

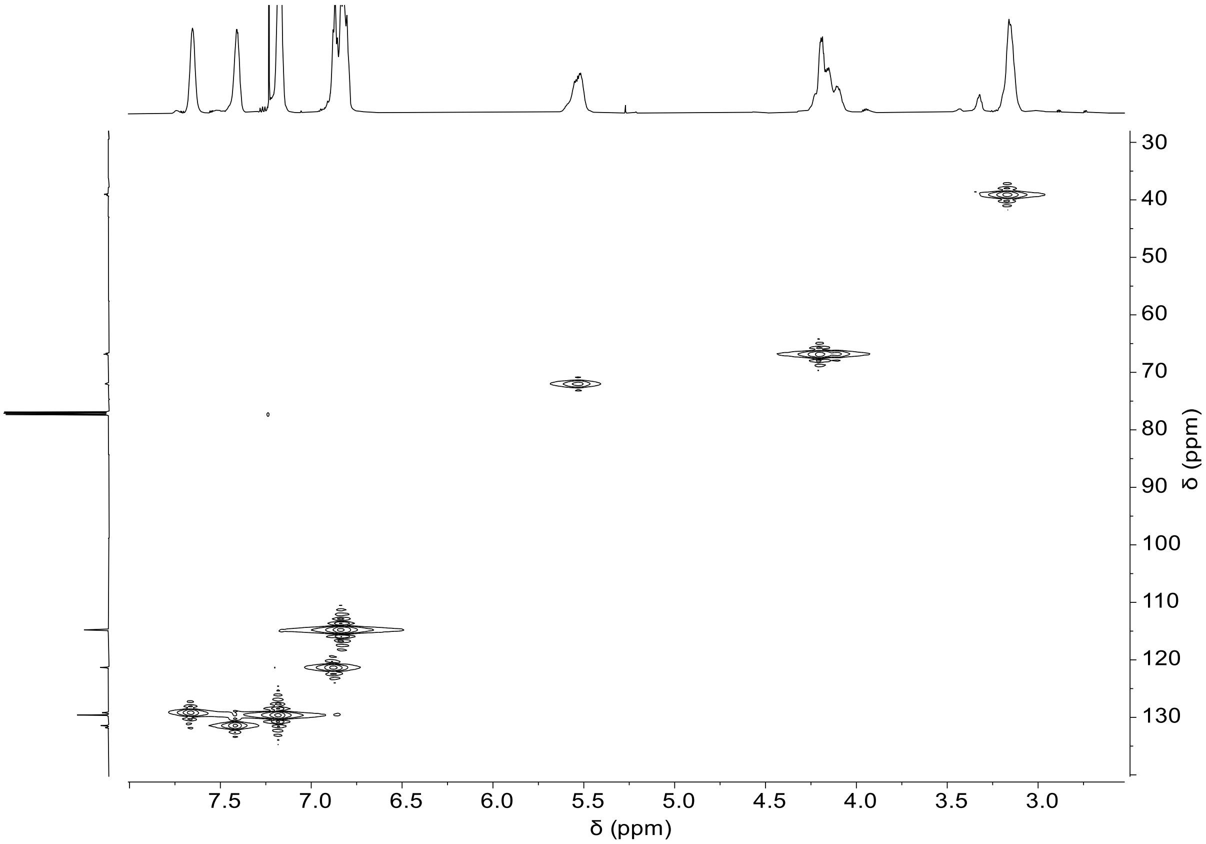

Figure S61: ^{1}H-^{13}C HSQC NMR spectrum ($CDCl_3$, 25°C) of the precipitated polymer corresponding to ESI table 1 run #8.

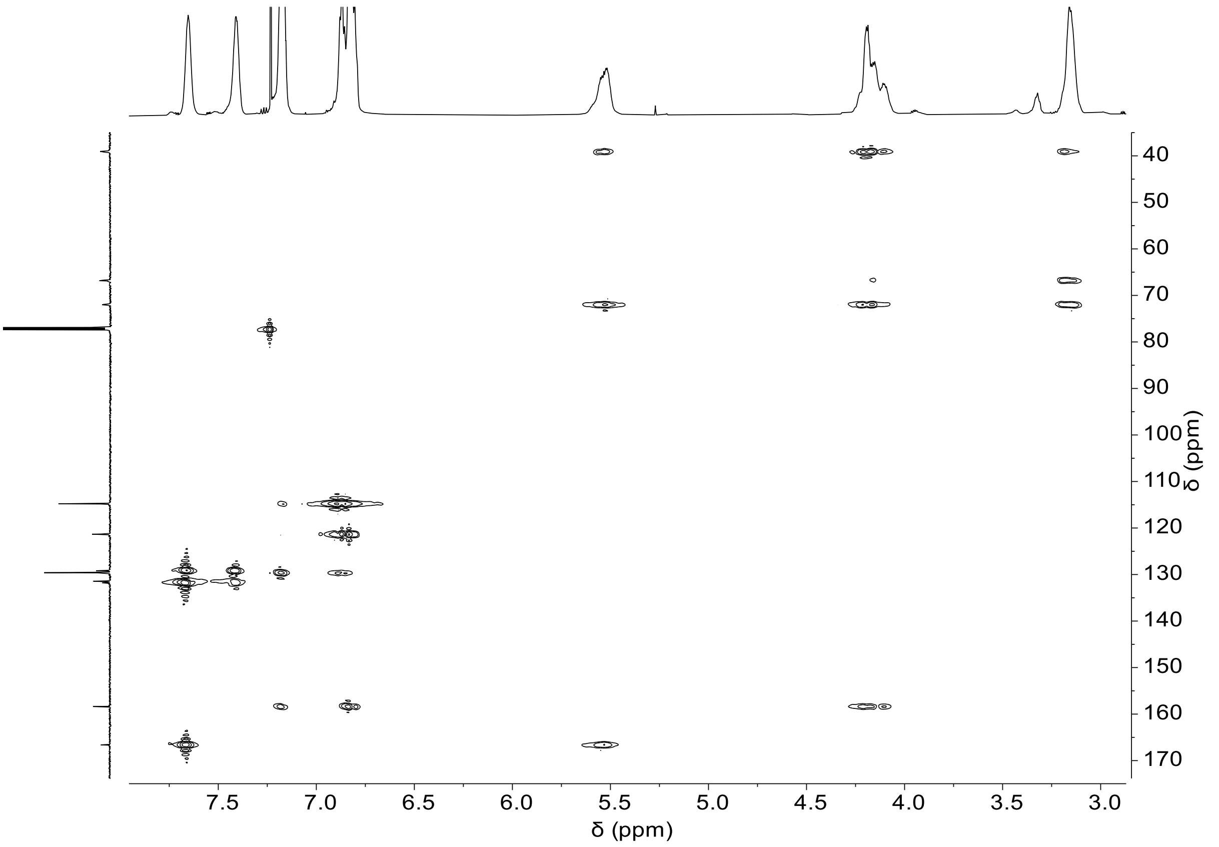

Figure S62: ^{1}H-^{13}C HMBC NMR spectrum ($CDCl_3$, 25°C) of the precipitated polymer corresponding to ESI table 1 run #8.

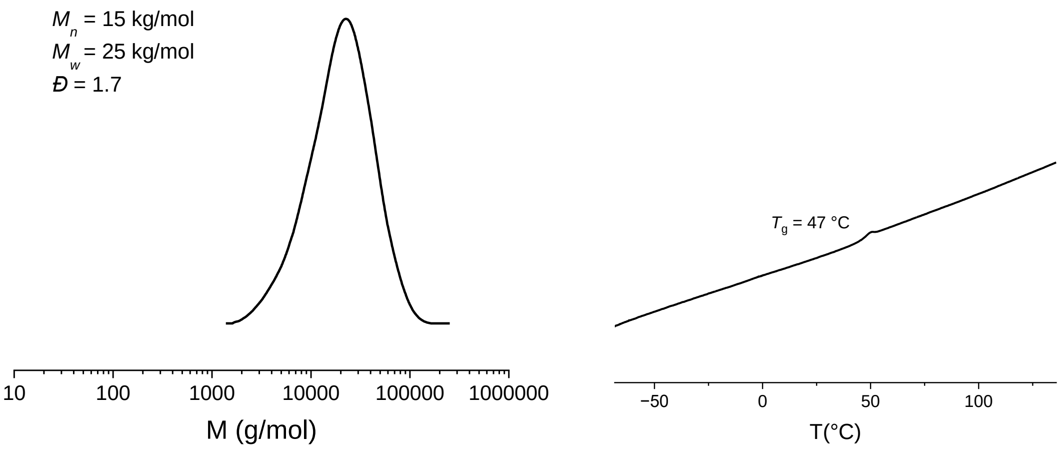

Figure S63: (left) GPC curve and (right) DSC 2nd heating curve of the precipitated polymer corresponding to ESI table 1 run #8.

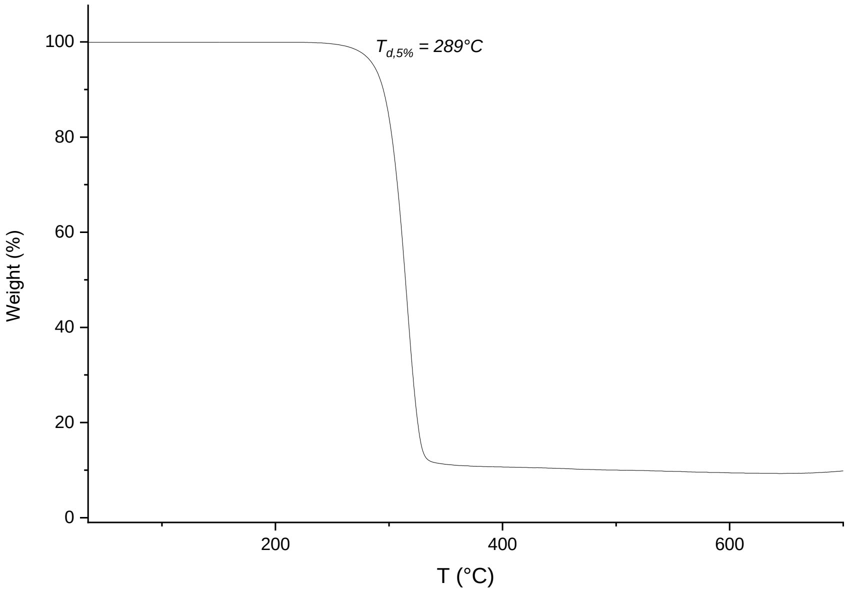

Figure S64: TGA data of the polymer corresponding to ESI table 1 run #8.

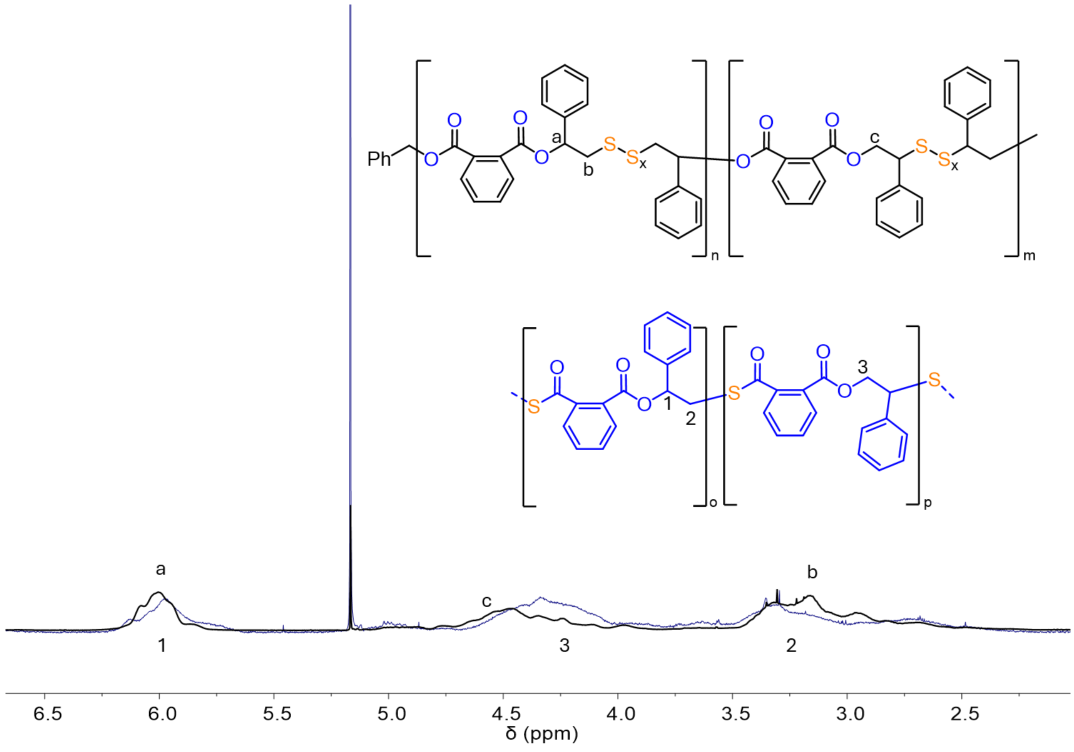

Figure S65: Zoom into the overlayed ^{1}H-NMR spetra of the terpolymer obtained from SO, PTA and S_8 (black) and the copolymer obtained from SO and PTA (blue).

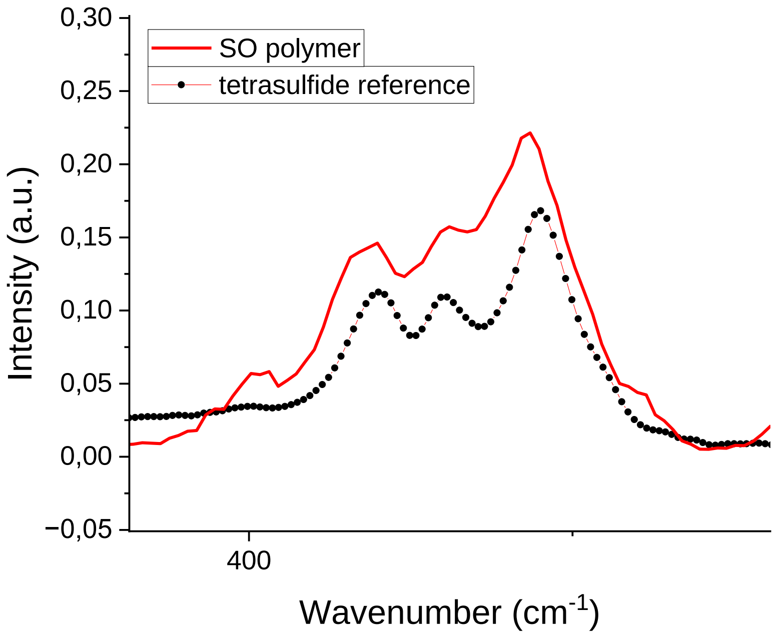

Figure S66: Zoom into the C-S_x-C stretching vibrations of the Raman spectra of the polymer corresponding to ESI table 1 run # 9 compared with R_2-tetrasulfide [R= -$CH_2CH_2CH_2Si(OCH_2CH_3)_3$] reference compound.

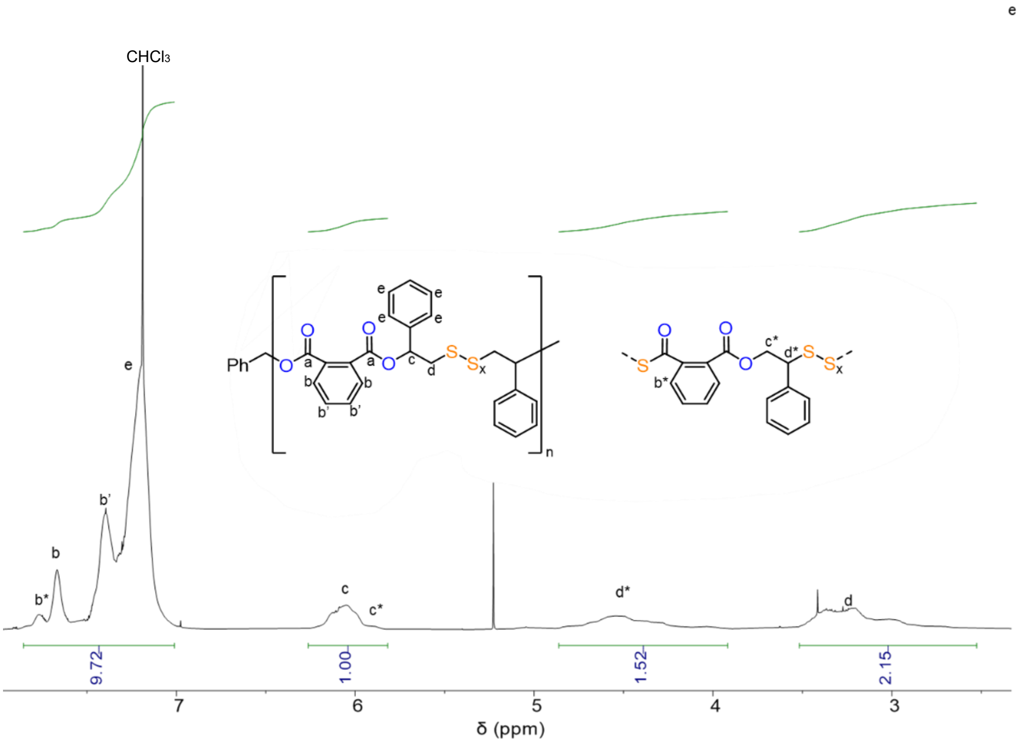

Figure S67: ^{1}H-NMR spectrum (600 MHz, $CDCl_3$, 25°C) of the precipitated polymer corresponding to ESI table 1 run #9.

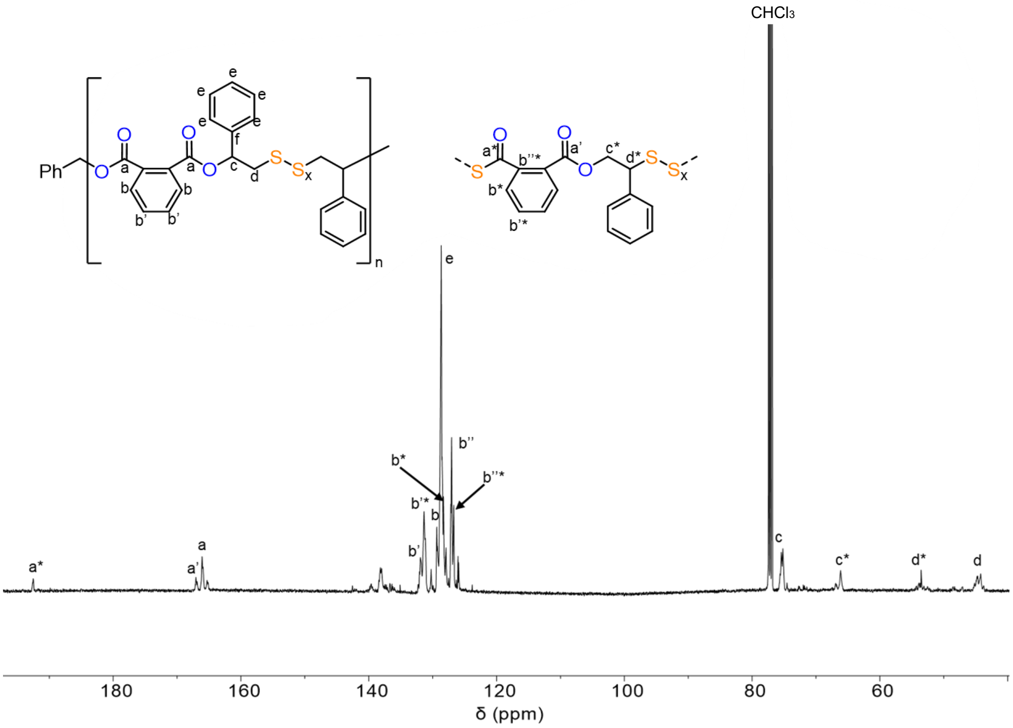

Figure S68: ^{13}C-NMR spectrum (151 MHz, $CDCl_3$, 25°C) of the precipitated polymer corresponding to ESI table 1 run #9.

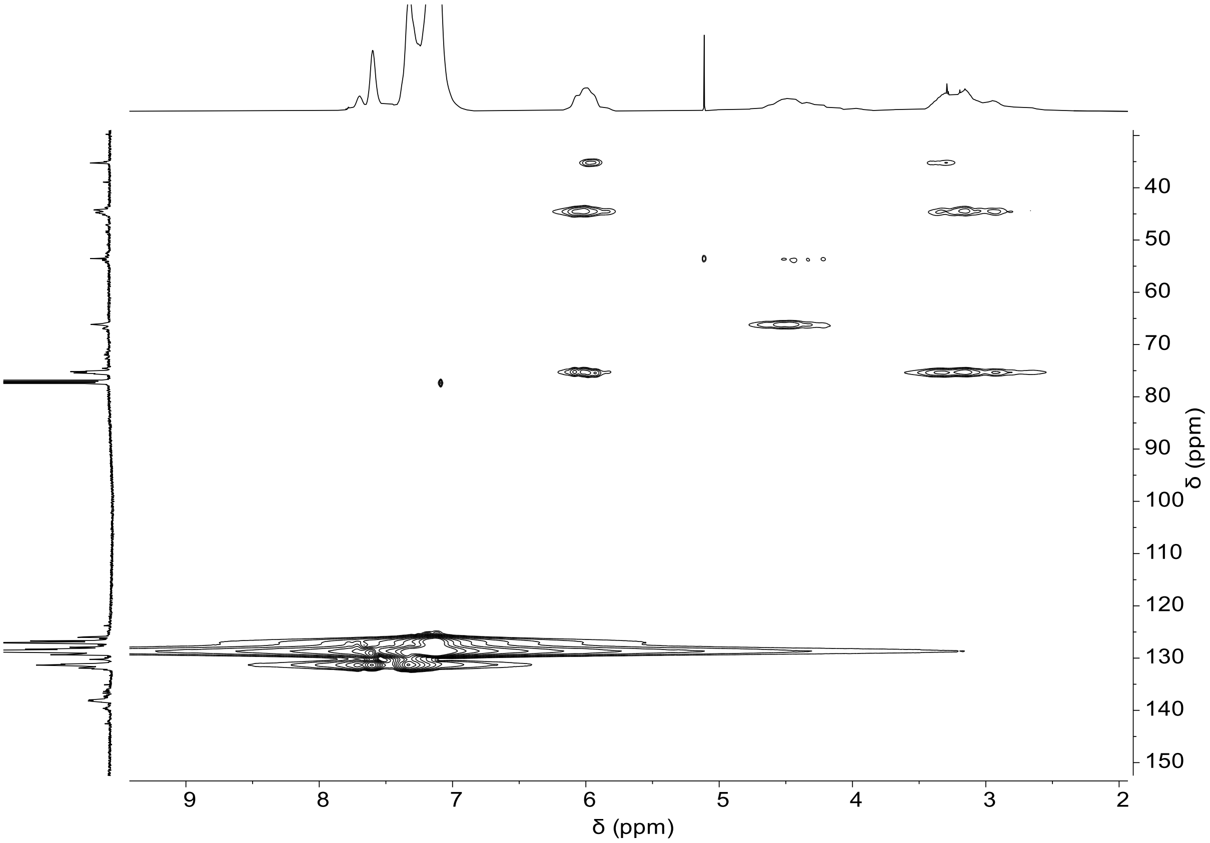

Figure S69: ^{1}H-^{13}C HSQC NMR spectrum ($CDCl_3$, 25°C) of the precipitated polymer corresponding to ESI table 1 run #9.

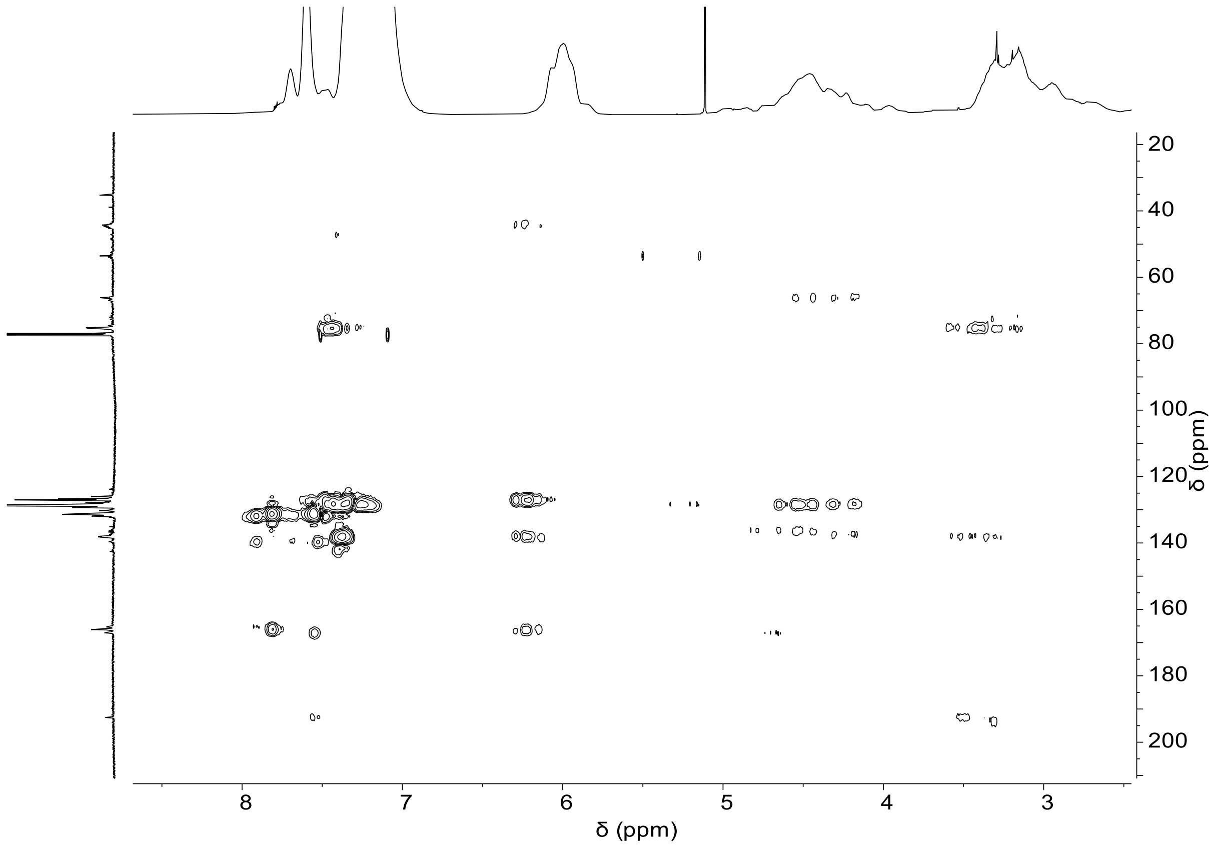

Figure S70: ^{1}H-^{13}C HMBC NMR spectrum ($CDCl_3$, 25°C) of the precipitated polymer corresponding to ESI table 1 run #9.

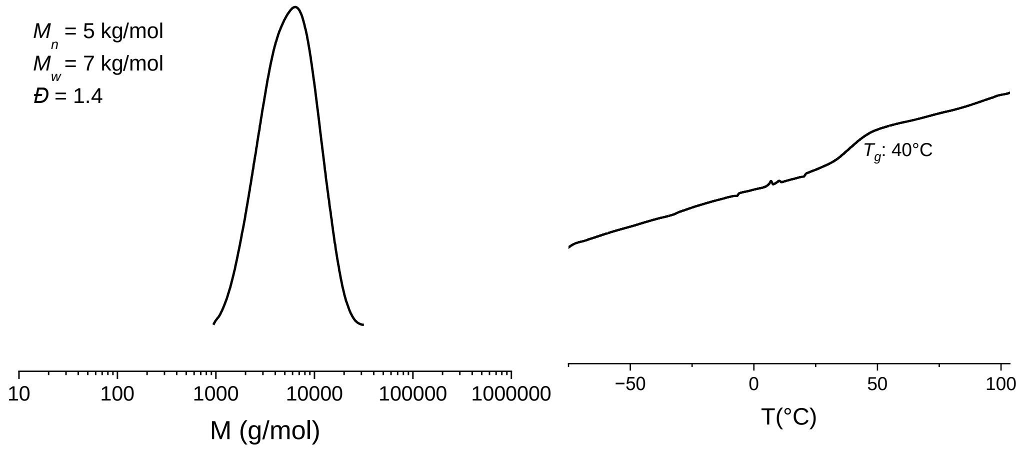

Figure S71: (left) GPC curve and (right) DSC 2nd heating curve of the precipitated polymer corresponding to ESI table 1 run #9.

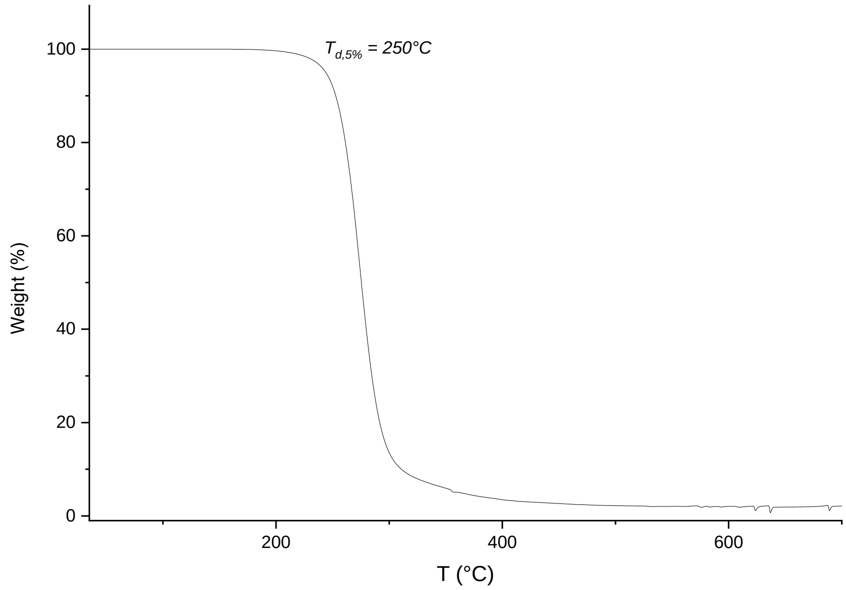

Figure S72: TGA data of the polymer corresponding to ESI table 1 run #9.

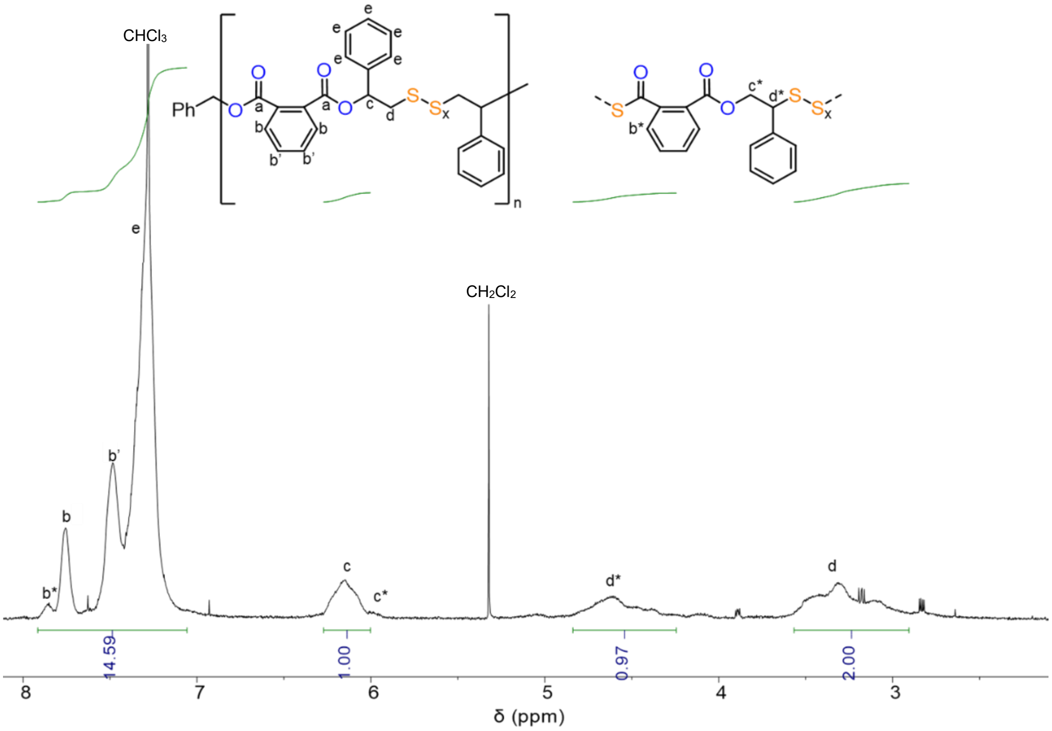

Figure S73: ^{1}H-NMR spectra (600 MHz, $CDCl_3$, 25°C) of the precipitated polymer corresponding to ESI table 1 run #10.

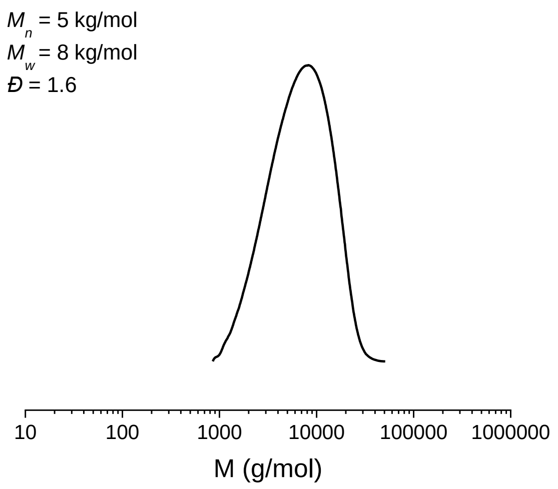

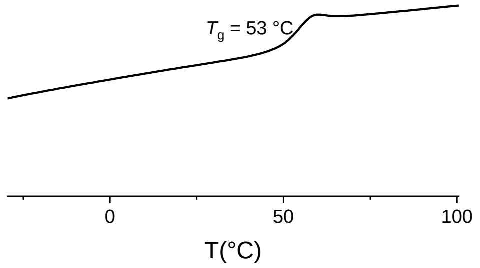

Figure S74: (left) GPC curve and (right) DSC 2nd heating curve of the precipitated polymer corresponding to ESI table 1 run #10.

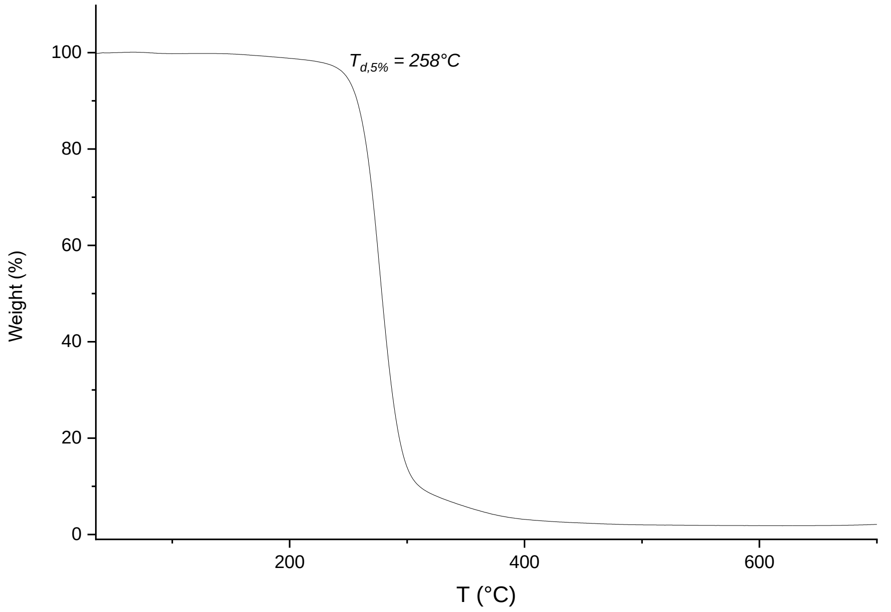

Figure S75: TGA data of the polymer corresponding to ESI table 1 run #10.

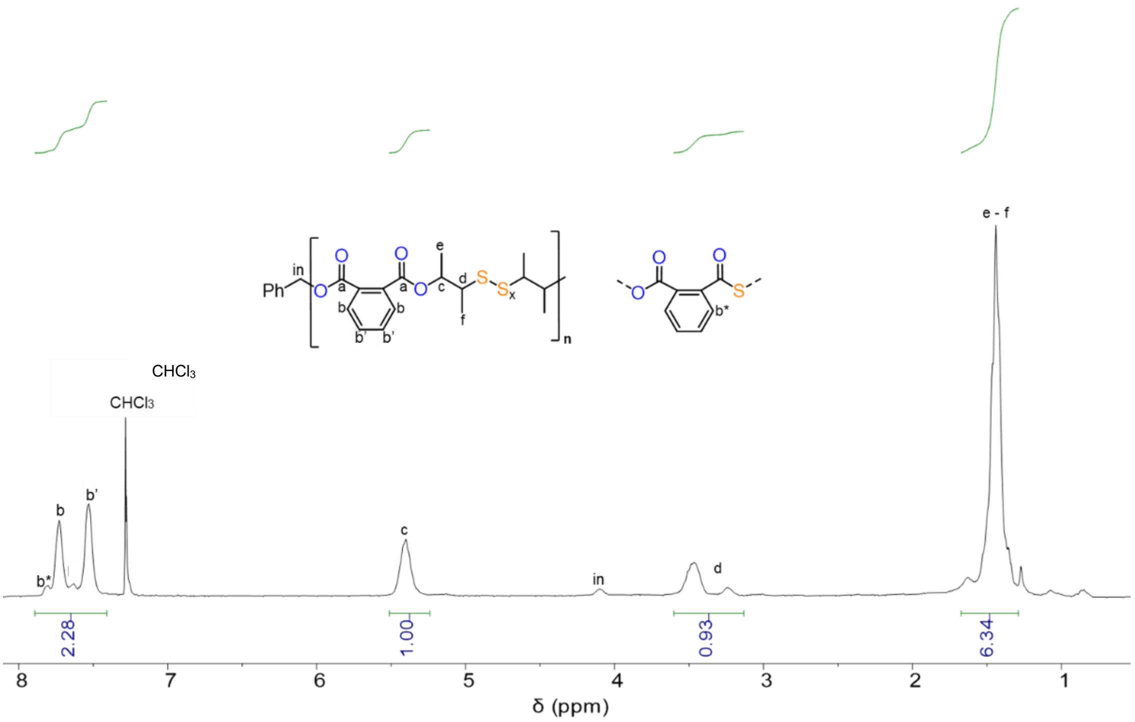

Figure S76: ^{1}H-NMR spectra (500 MHz, $CDCl_3$, 25°C) of the precipitated polymer corresponding to ESI table 1 run #11.

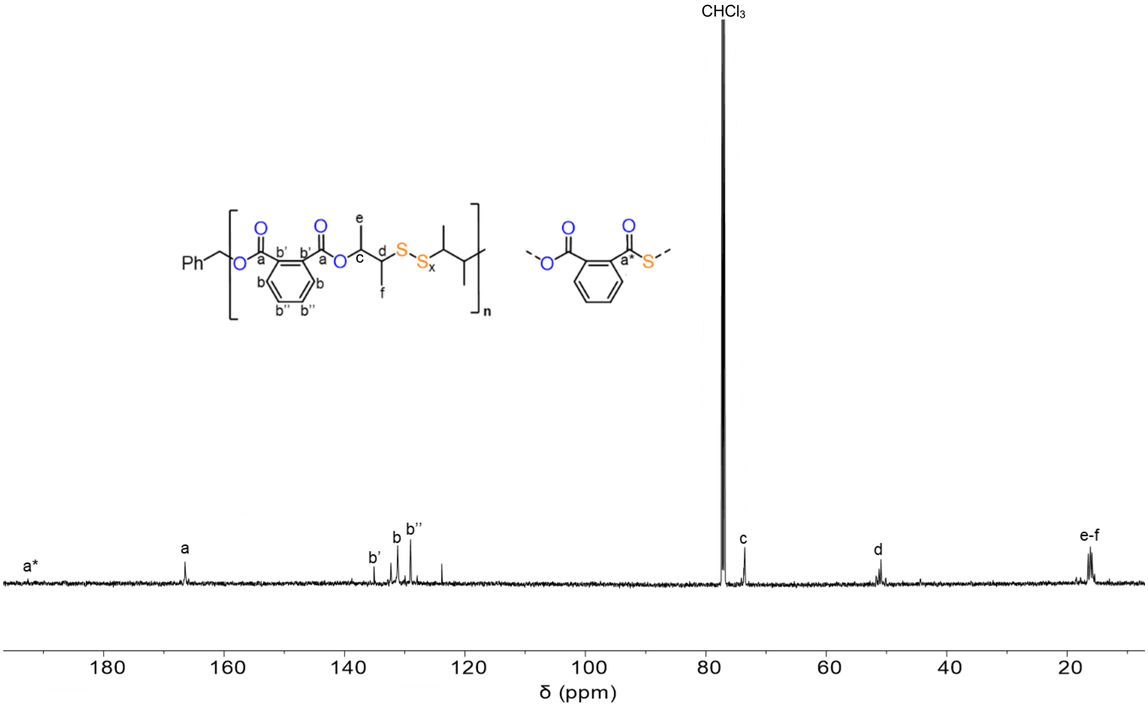

Figure S77: ^{13}C-NMR spectrum (151 MHz, $CDCl_3$, 25°C) of the precipitated polymer corresponding to ESI table 1 run #11.

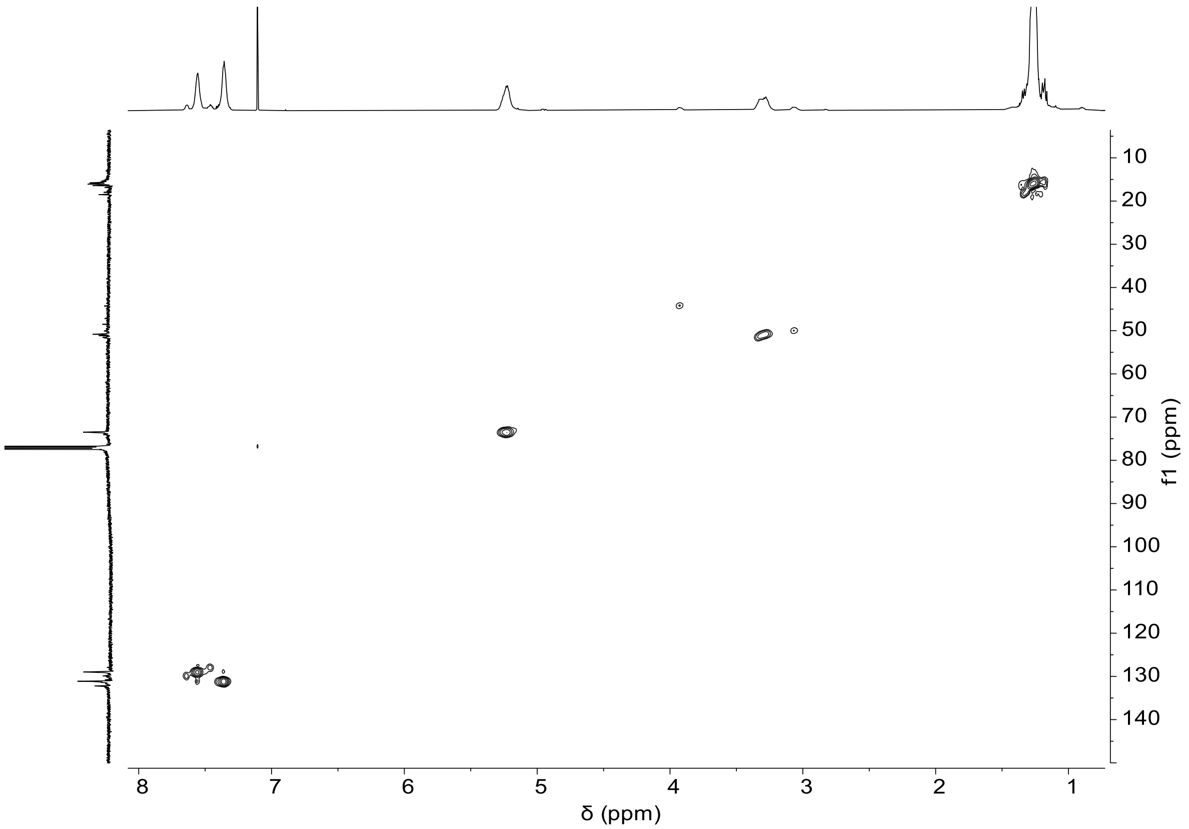

Figure S78: ^{1}H-^{13}C HSQC NMR spectrum ($CDCl_3$, 25°C) of the precipitated polymer corresponding to ESI table 1 run #11.

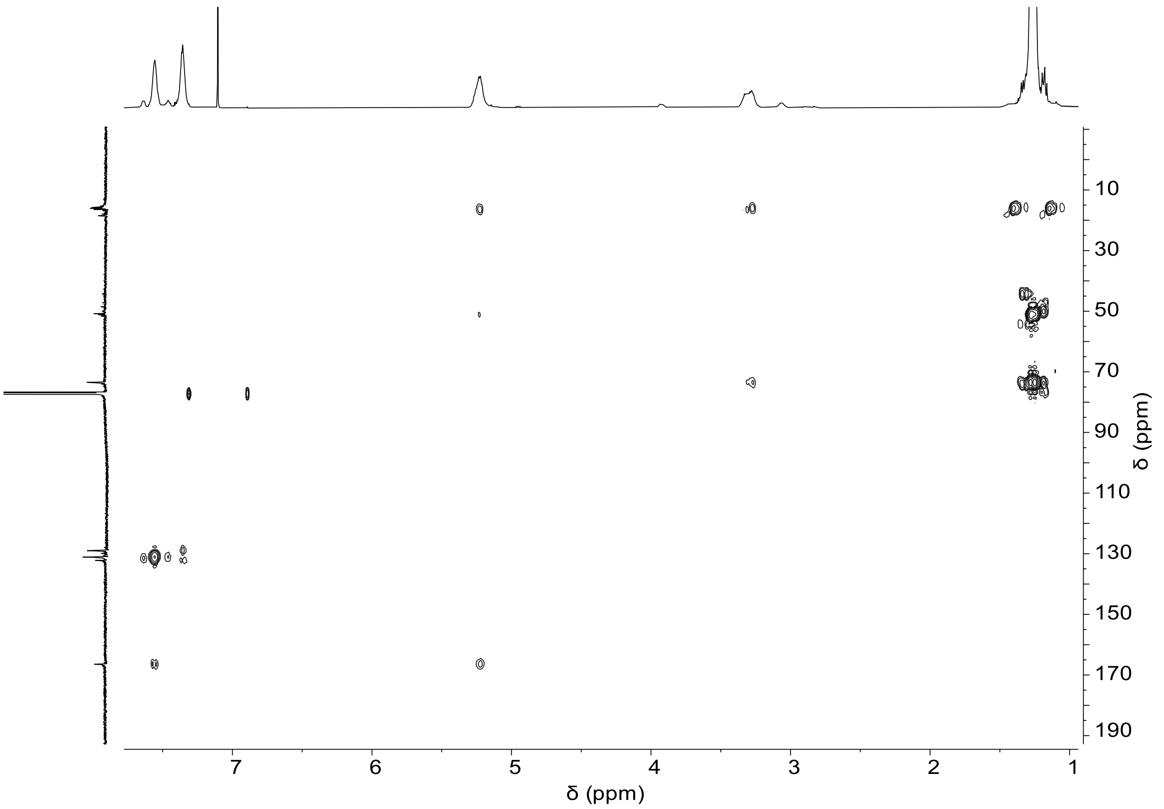

Figure S79: ^{1}H-^{13}C HMBC NMR spectrum ($CDCl_3$, 25°C) of the precipitated polymer corresponding to ESI table 1 run #11.

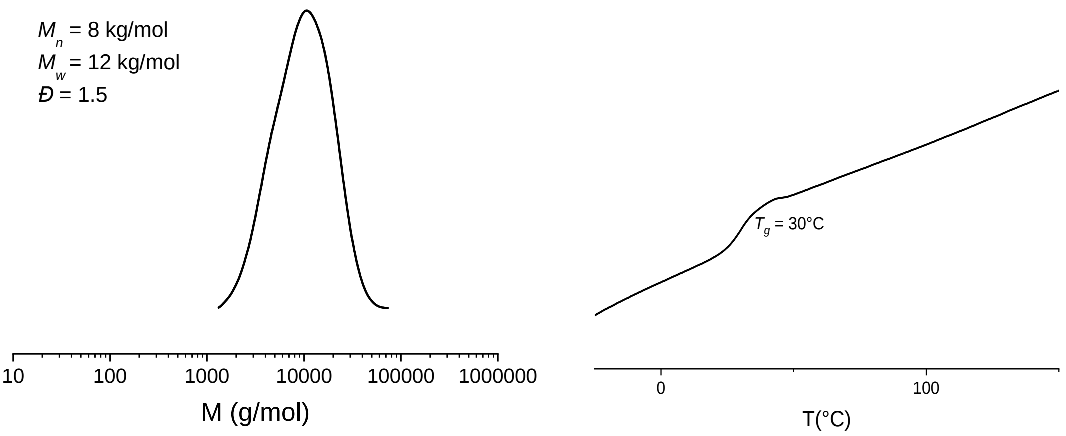

Figure S80: (left) GPC curve and (right) DSC 2nd heating curve of the precipitated polymer corresponding to ESI table 1 run #11.

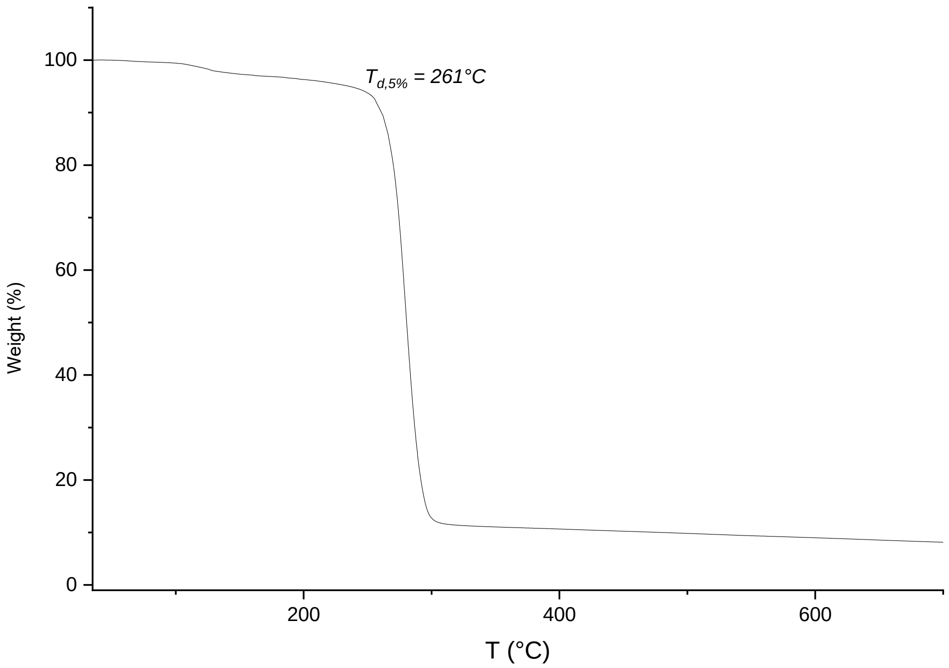

Figure S81: TGA data of the polymer corresponding to ESI table 1 run #11.

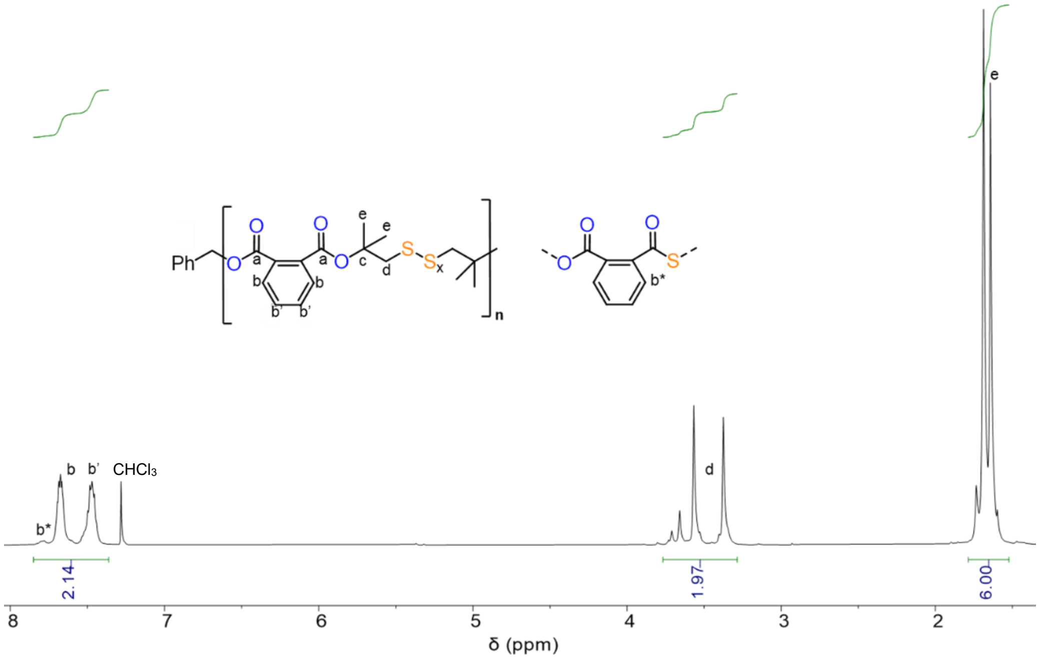

Figure S82: ^{1}H-NMR spectrum (500 MHz, $CDCl_3$, 25°C) of the precipitated polymer corresponding to ESI table 1 run #12.

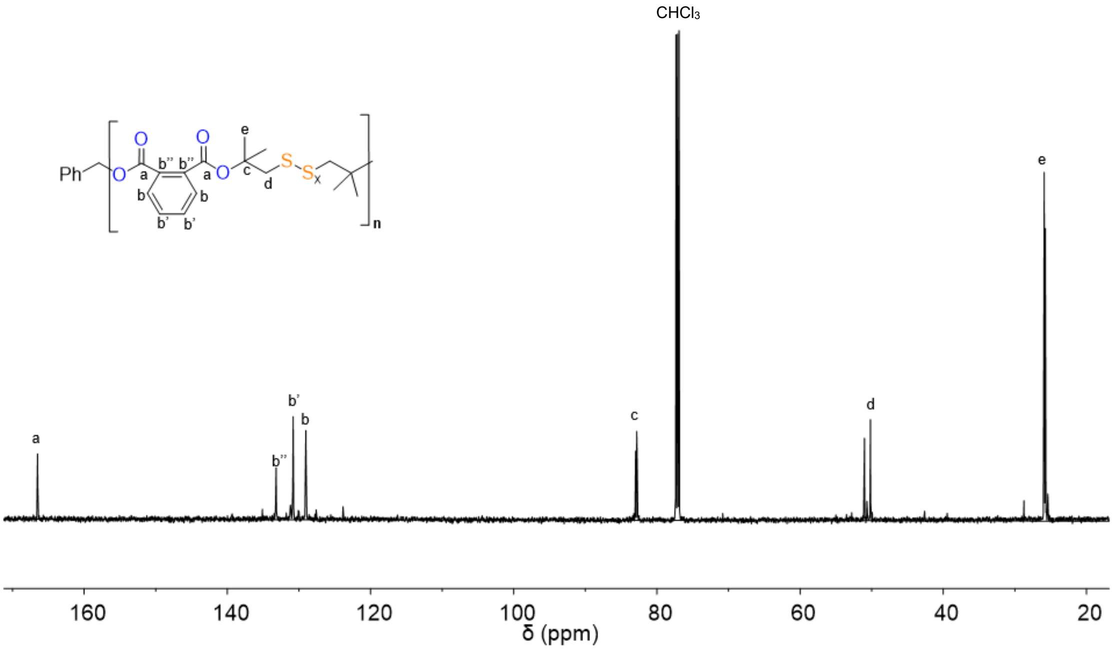

Figure S83: ^{13}C-NMR spectrum (151 MHz, $CDCl_3$, 25°C) of the precipitated polymer corresponding to ESI table 1 run #12.

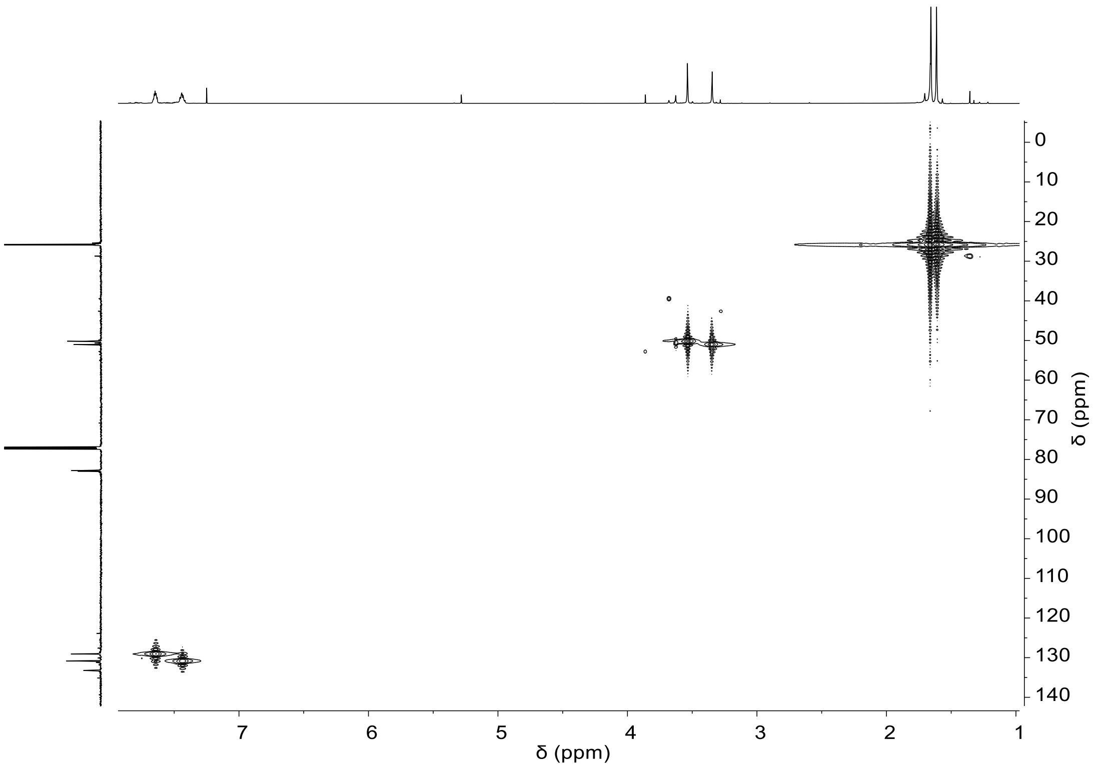

Figure S84: ^{1}H-^{13}C HSQC NMR spectrum ($CDCl_3$, 25°C) of the precipitated polymer corresponding to ESI table 1 run #12.

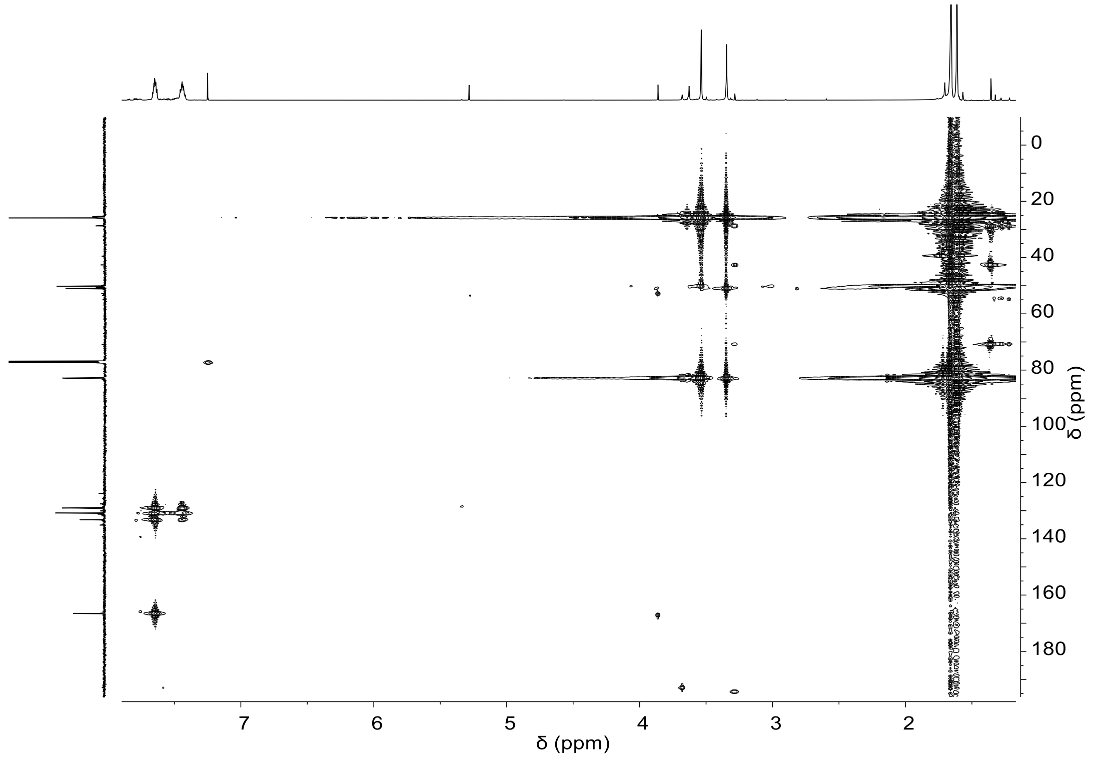

Figure S85: ^{1}H-^{13}C HMBC NMR spectrum ($CDCl_3$, 25°C) of the precipitated polymer corresponding to ESI table 1 run #12.

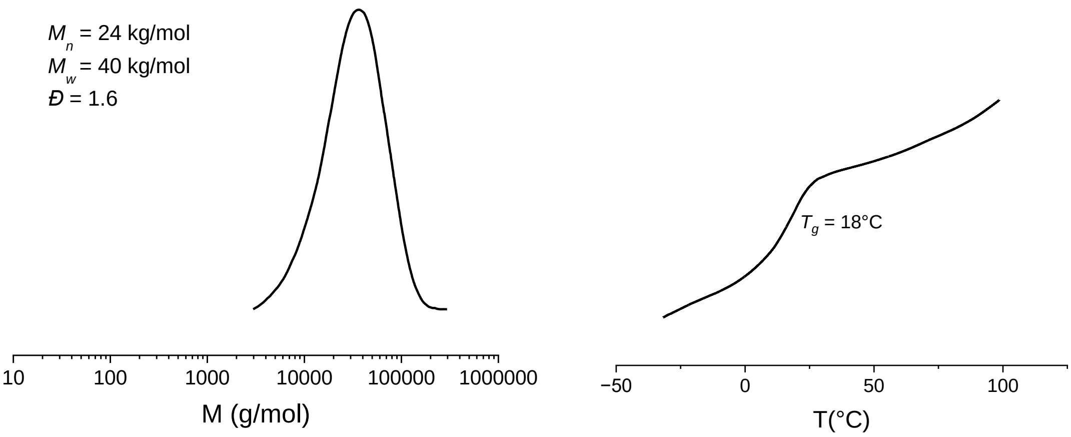

Figure S86: (left) GPC curve and (right) DSC 2nd heating curve of the precipitated polymer corresponding to ESI table 1 run #12.

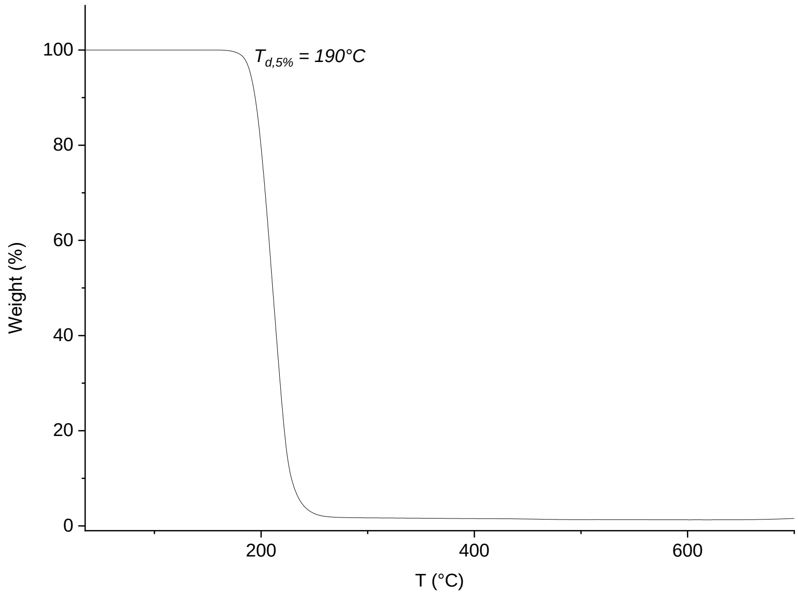

Figure S87: TGA data of the polymer corresponding to ESI table 1 run #12.

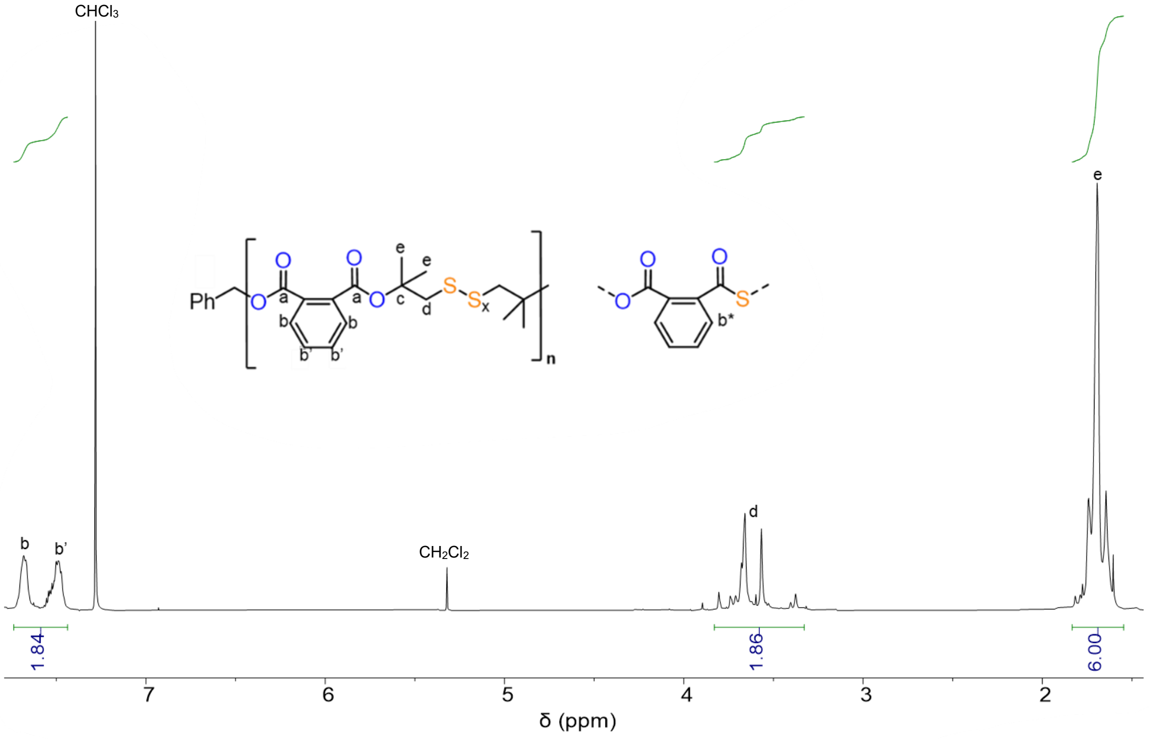

Figure S88: ^{1}H-NMR spectrum (500 MHz, $CDCl_3$, 25°C) of the precipitated polymer corresponding to ESI table 1 run #13.

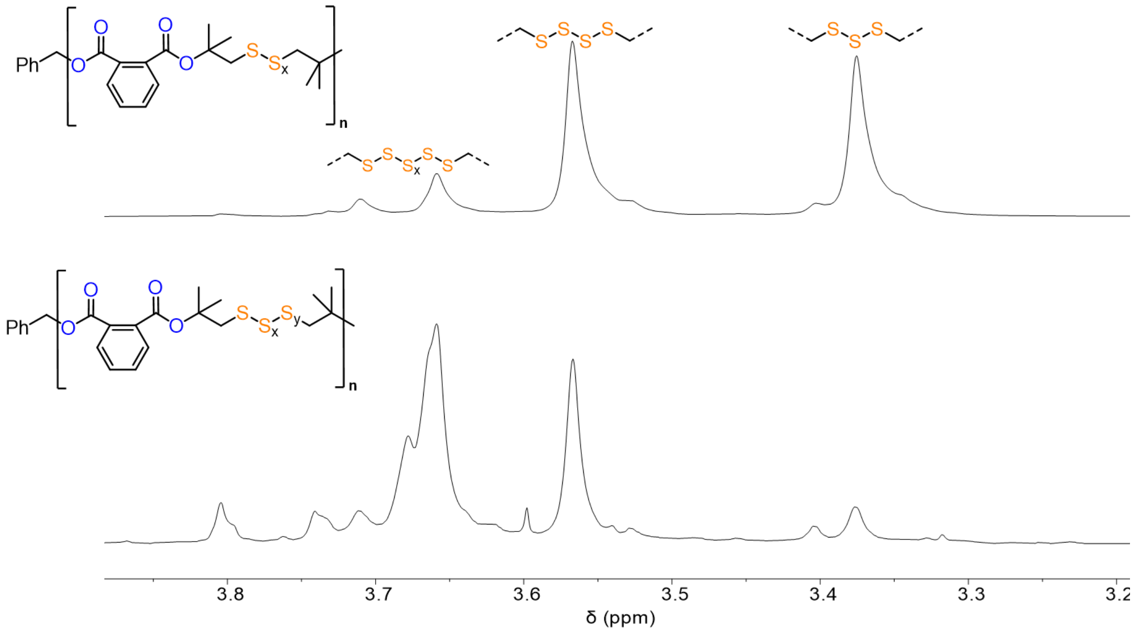

Figure S89: ^{1}H-NMR spectra (500 MHz, $CDCl_3$, 25°C) of the precipitated polymers corresponding to ESI table 1 run #12 (up) and #13 (down), zoomed in into the proton in alpha position to the polysulfide region.

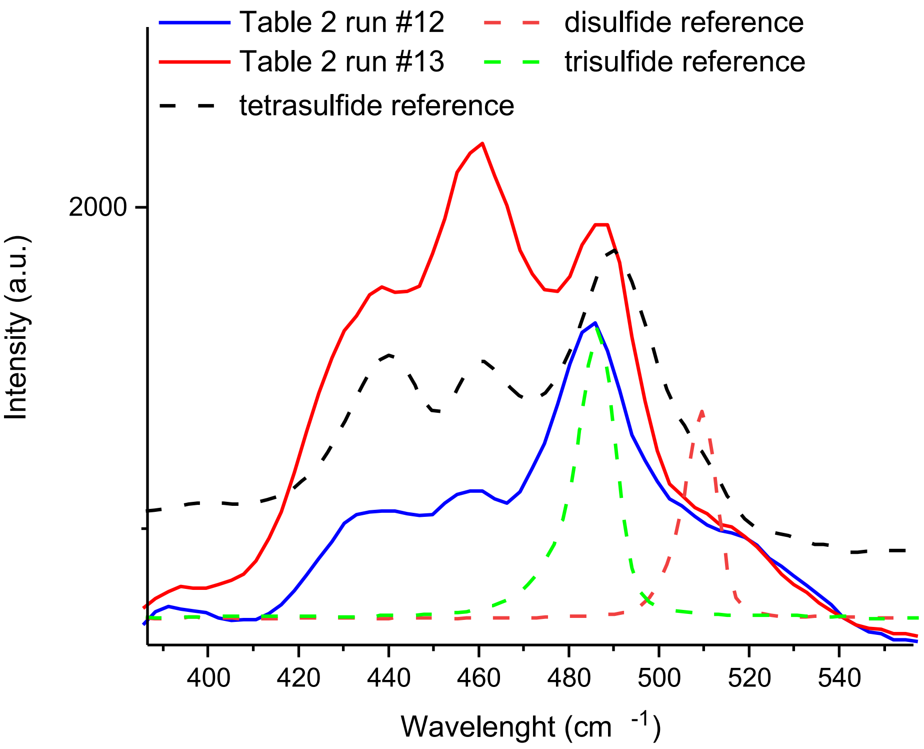

Figure S90: Zoom into the C-S_x-C stretching vibrations of the Raman spectra of the polymer corresponding to ESI table 1 run # 12 and #13, compared with dimethyl disulfide, dimethyl trisulfide, and R_2-tetrasulfide [R= -$CH_2CH_2CH_2Si(OCH_2CH_3)_3$] reference compounds.

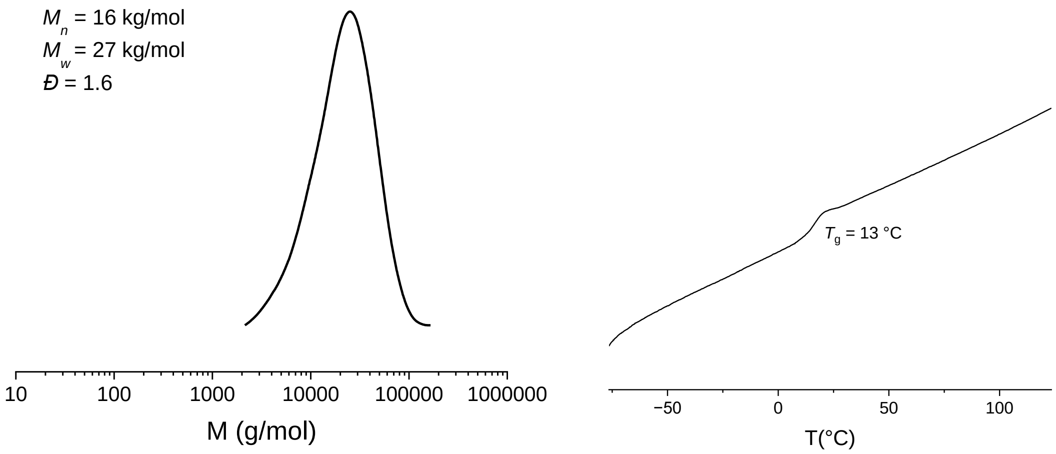

Figure S91: (left) GPC curve and (right) DSC 2nd heating curve of the precipitated polymer corresponding to ESI table 1 run #13.

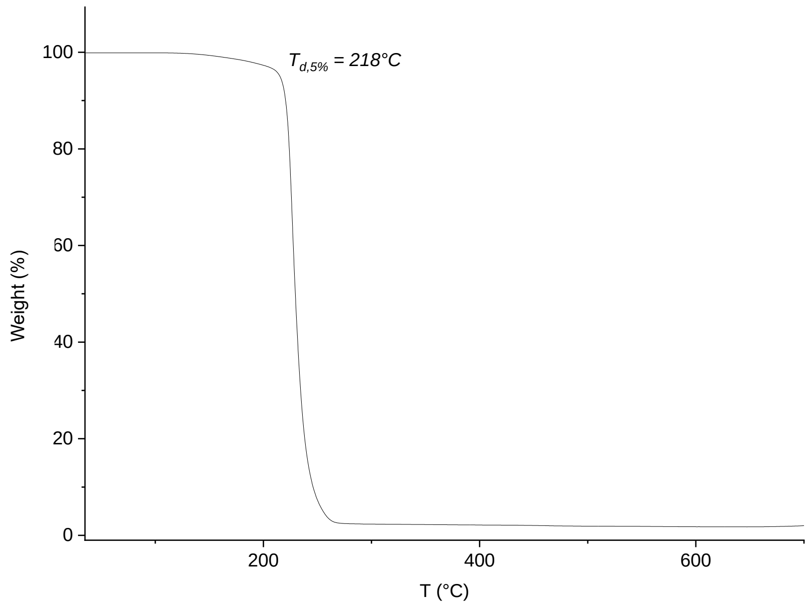

Figure S92: TGA data of the polymer corresponding to ESI table 1 run #13.

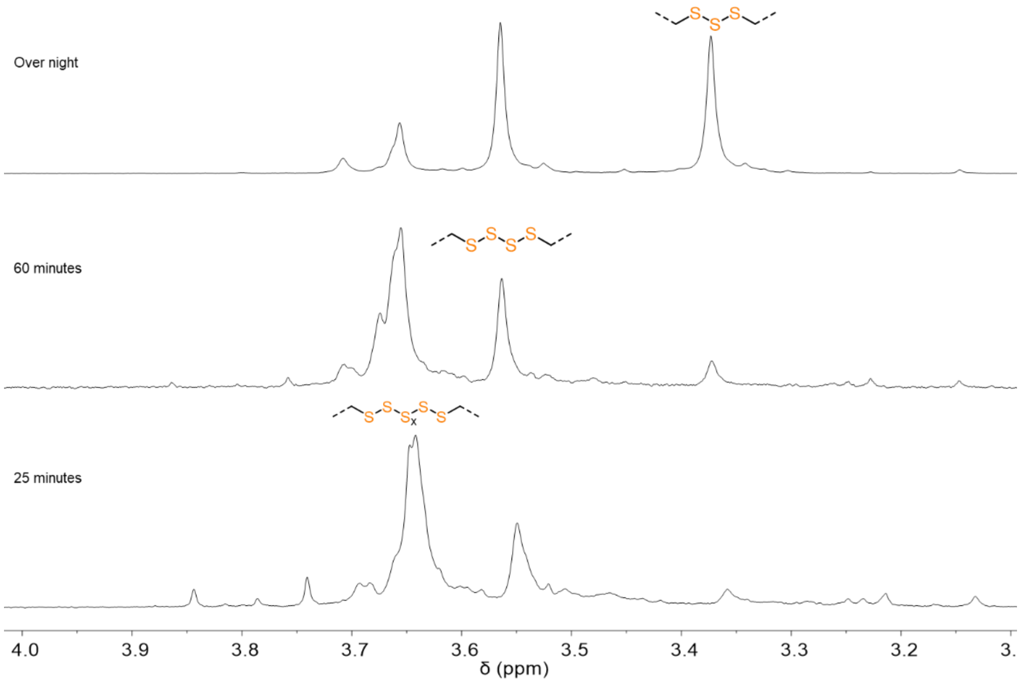

Figure S93: Zoom into overlayed ^{1}H-NMR spectra of IBO terpolymerisation over time (1 eq. catalyst: 500 eq. IBO: 200 eq. PTA: 200 eg. S at 100°C) revealing that over time longer polysulfide links decrease in quantity in favour of shorter trisulfide links and therefore polysulfide links remain reactive during ROTERP.

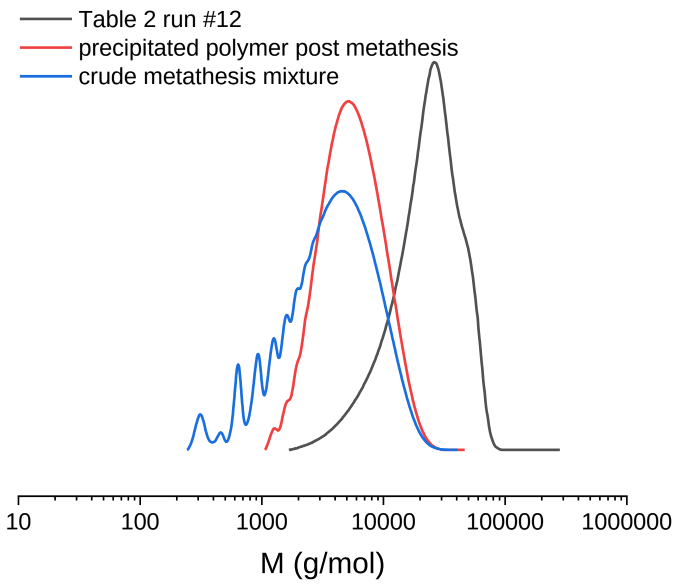

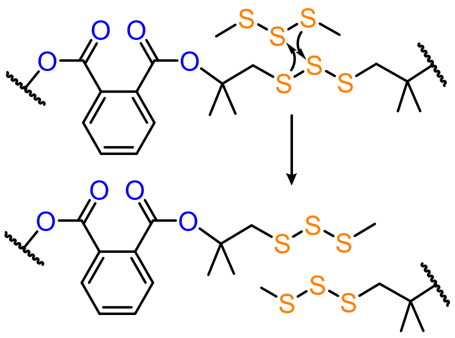

Figure S94: Comparison of the GPC signals obtained from the polymer corresponding to Table 2 run #12 and the same polymer after being exposed to dimethyl trisulfide at 100°C in a PO solution. The metathesis reaction was analysed with GPC before and after being purified by precipitation, the crude mixture shows the presence of smaller oligomers generated from the metathesis reaction. Scheme depicts an example chain cleavage pathway via spontaneous metathesis of polymer with Me_2S_3.

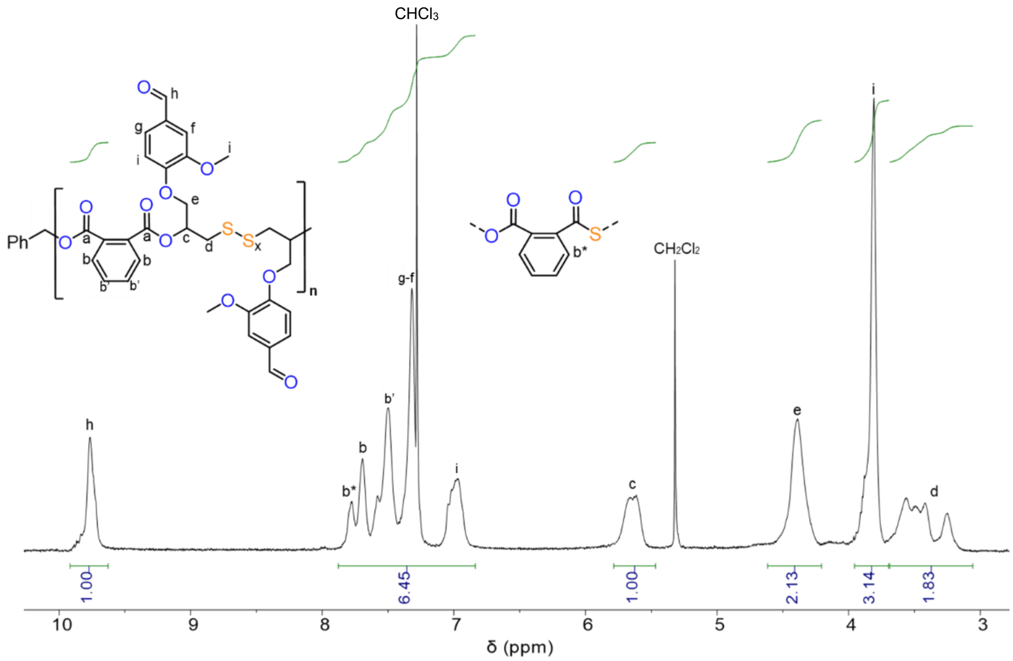

Figure S95: ^{1}H-NMR spectrum (500 MHz, $CDCl_3$, 25°C) of the precipitated polymer corresponding to ESI table 1 run #14.

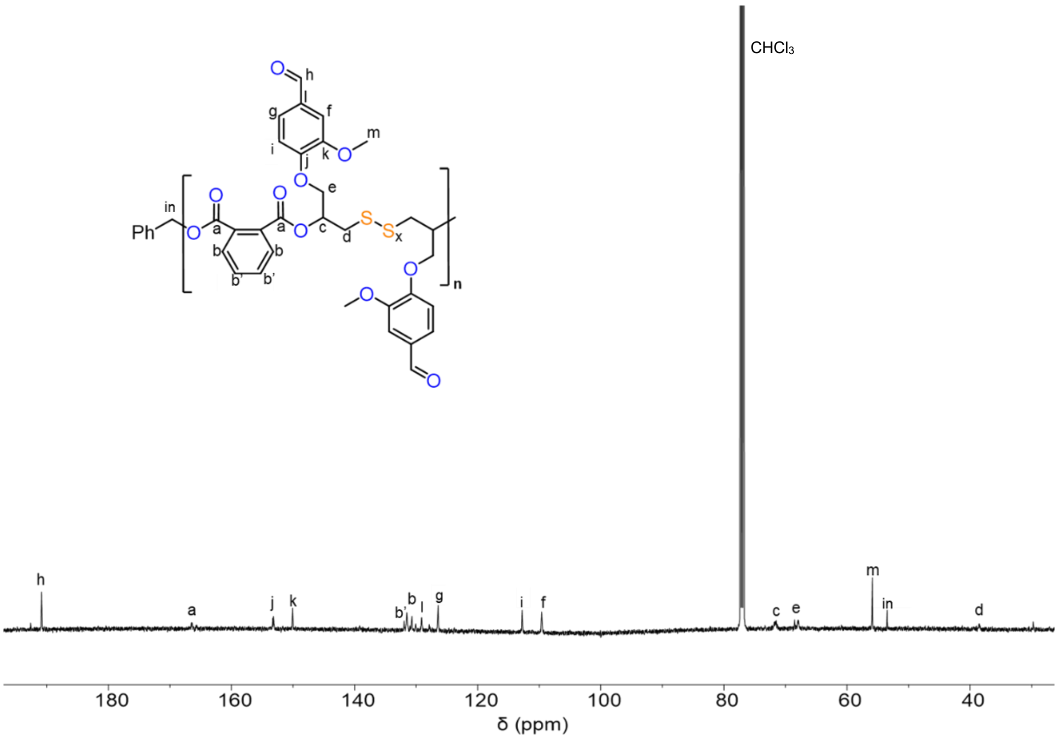

Figure S96: ^{13}C-NMR spectrum (151 MHz, $CDCl_3$, 25°C) of the precipitated polymer corresponding to ESI table 1 run #14.

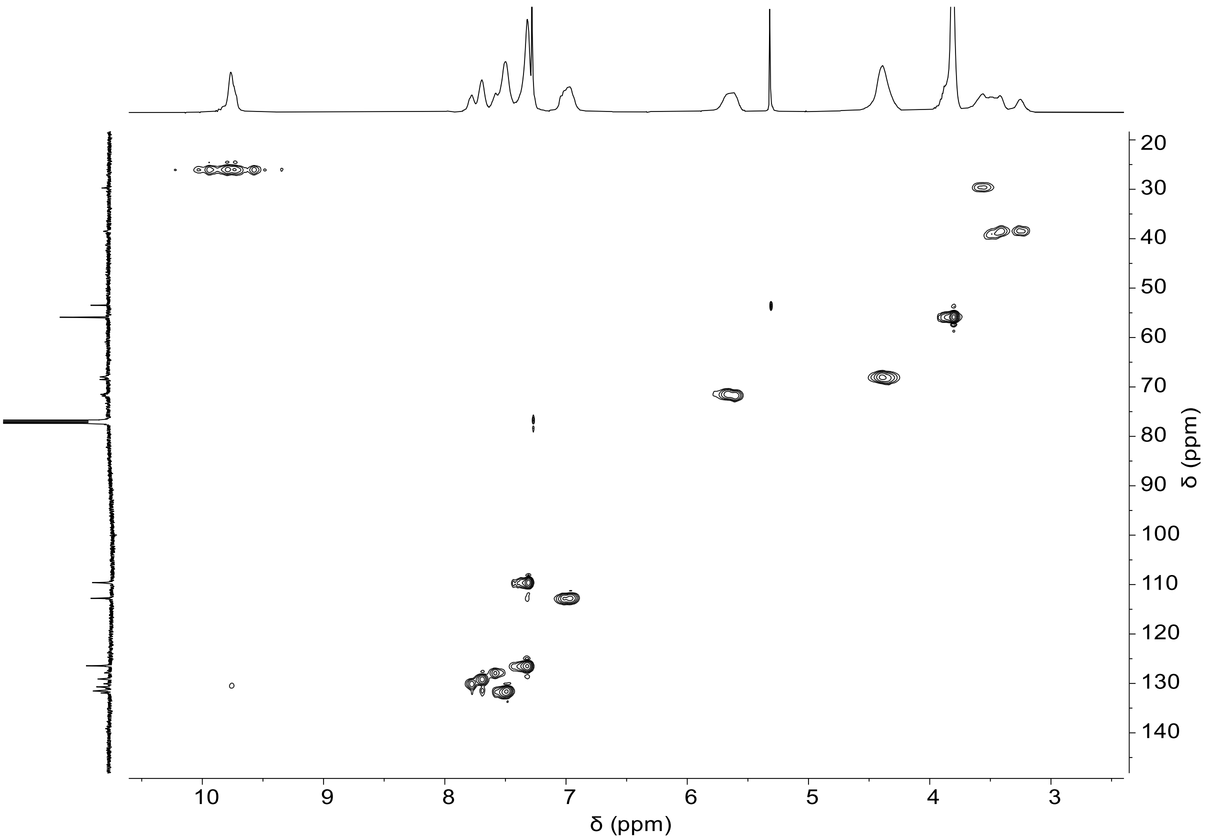

Figure S97: ^{1}H-^{13}C HSQC NMR spectrum ($CDCl_3$, 25°C) of the precipitated polymer corresponding to ESI table 1 run #14.

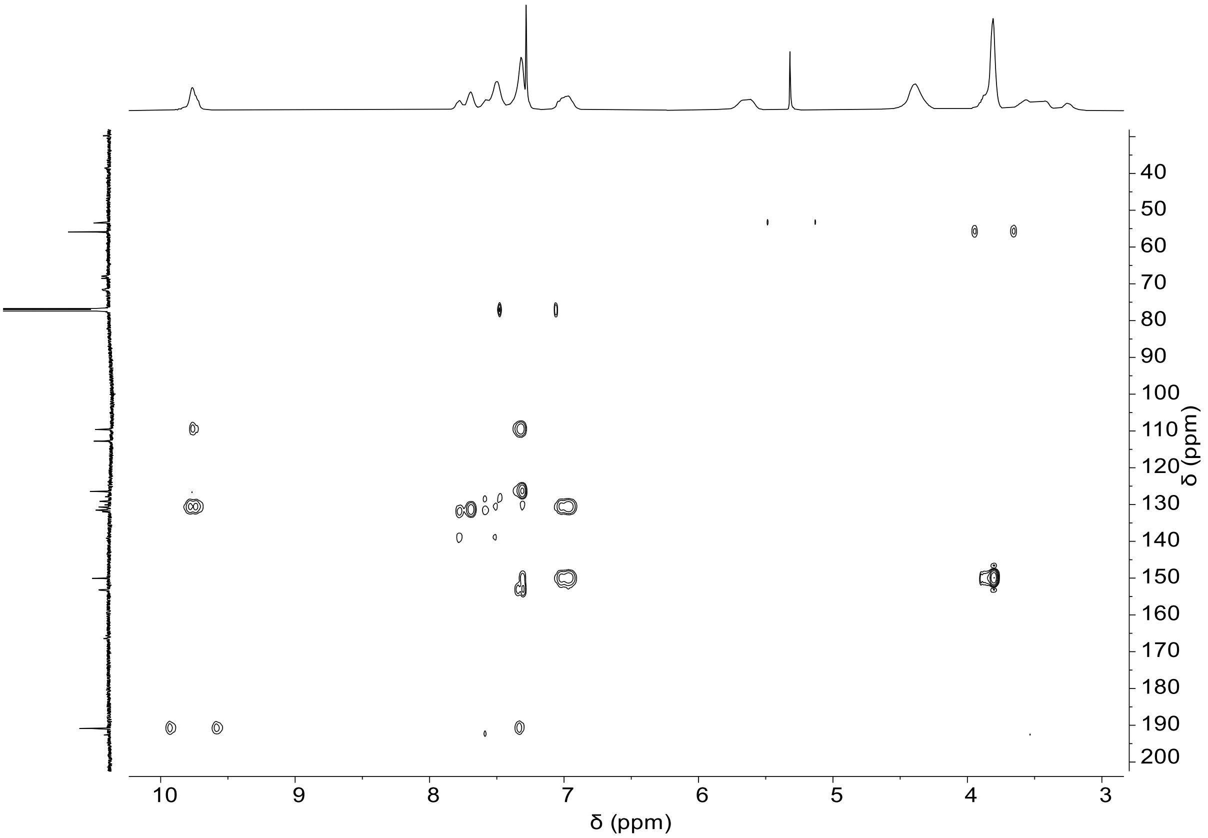

Figure S98: ^{1}H-^{13}C HMBC NMR spectrum ($CDCl_3$, 25°C) of the precipitated polymer corresponding to ESI table 1 run #14.

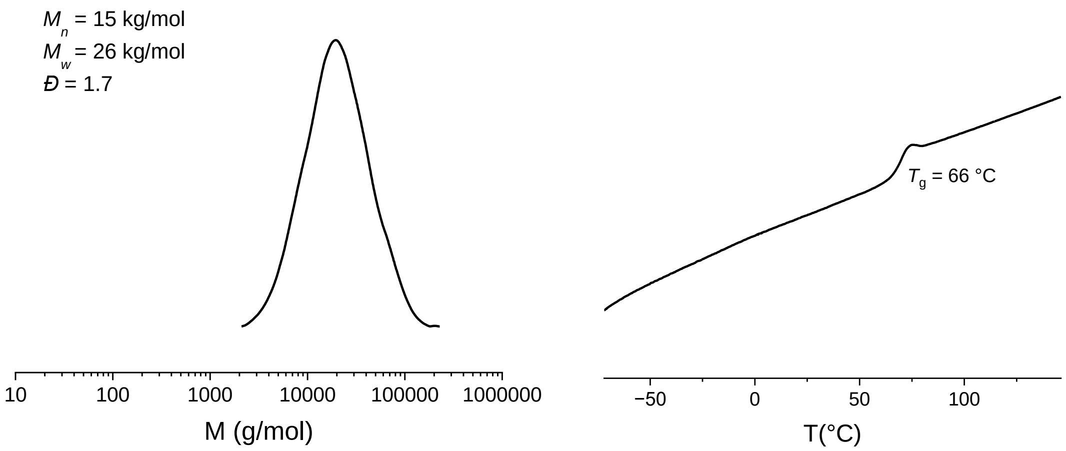

Figure S99: (left) GPC curve and (right) DSC 2nd heating curve of the precipitated polymer corresponding to ESI table 1 run #14.

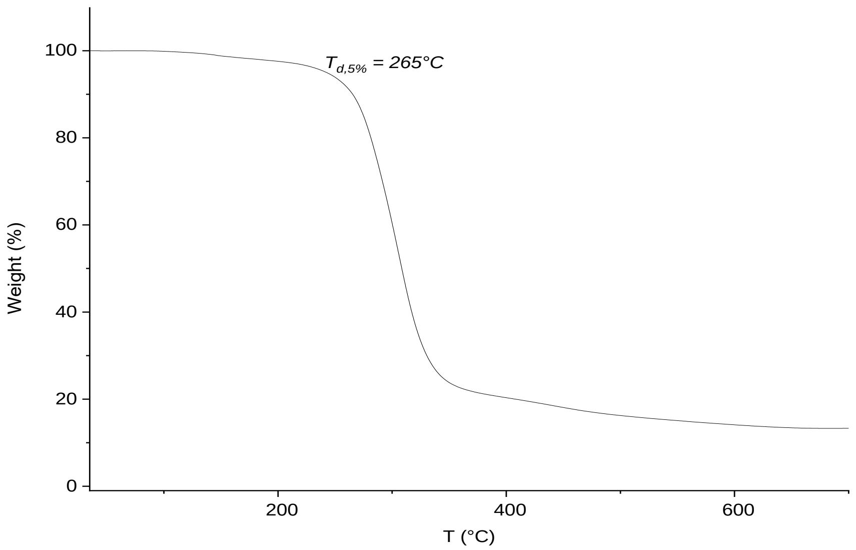

Figure S100: TGA data of the polymer corresponding to ESI table 1 run #14.

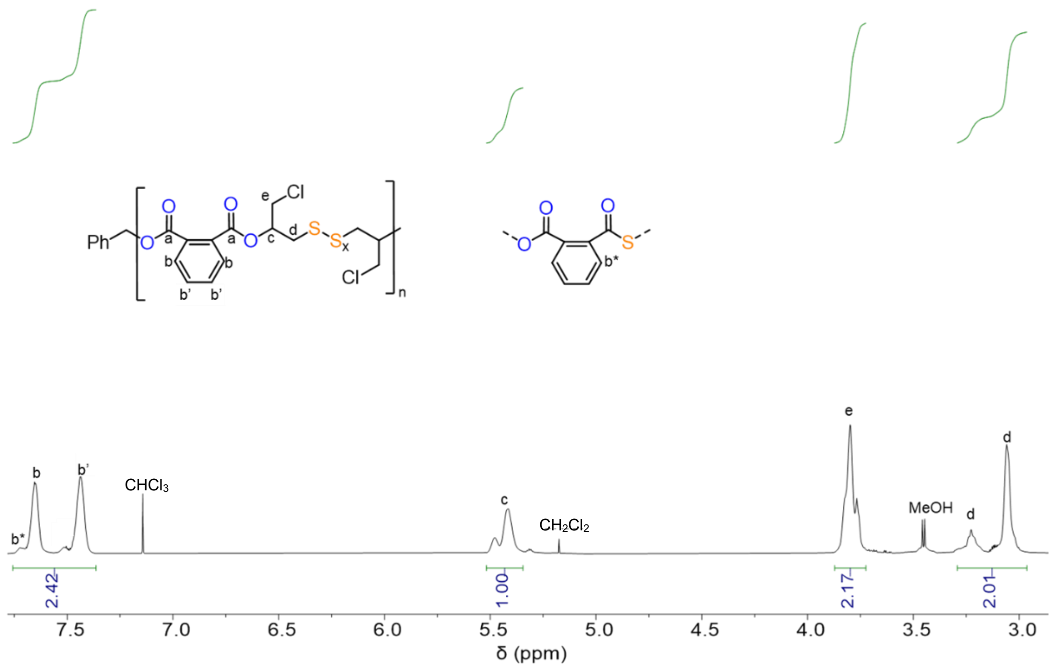

Figure S101: ^{1}H-NMR spectrum (500 MHz, $CDCl_3$, 25°C) of the precipitated polymer corresponding to ESI table 1 run #15.

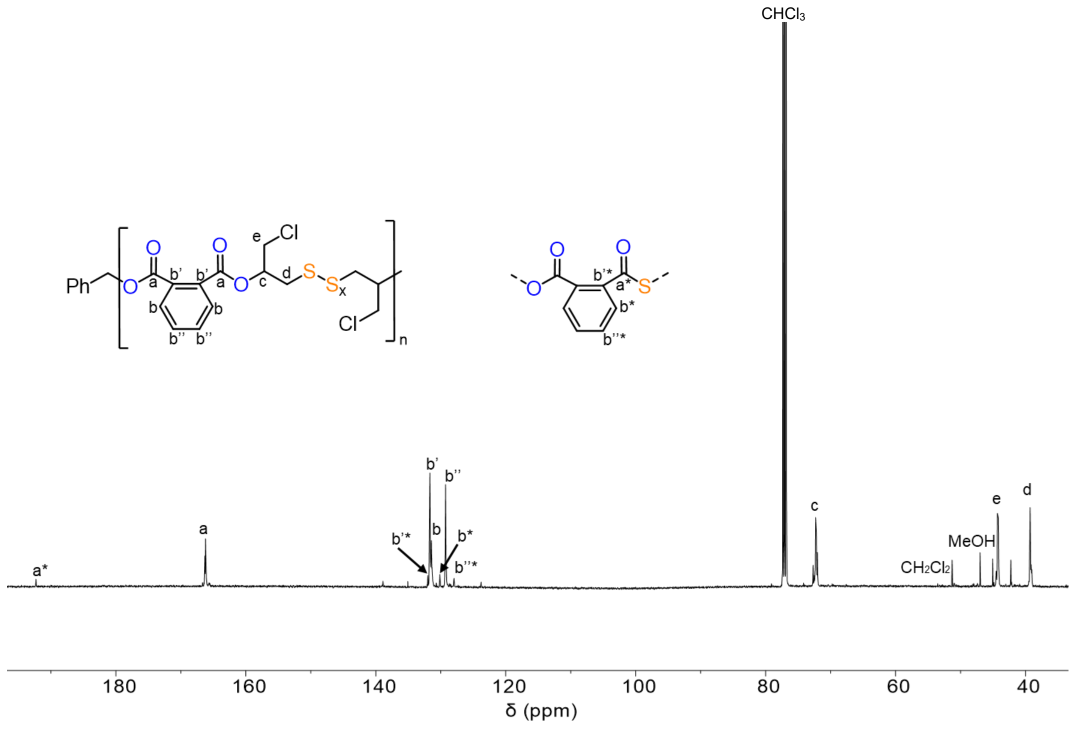

Figure S102: ^{13}C-NMR spectrum (151 MHz, $CDCl_3$, 25°C) of the precipitated polymer corresponding to ESI table 1 run #15.

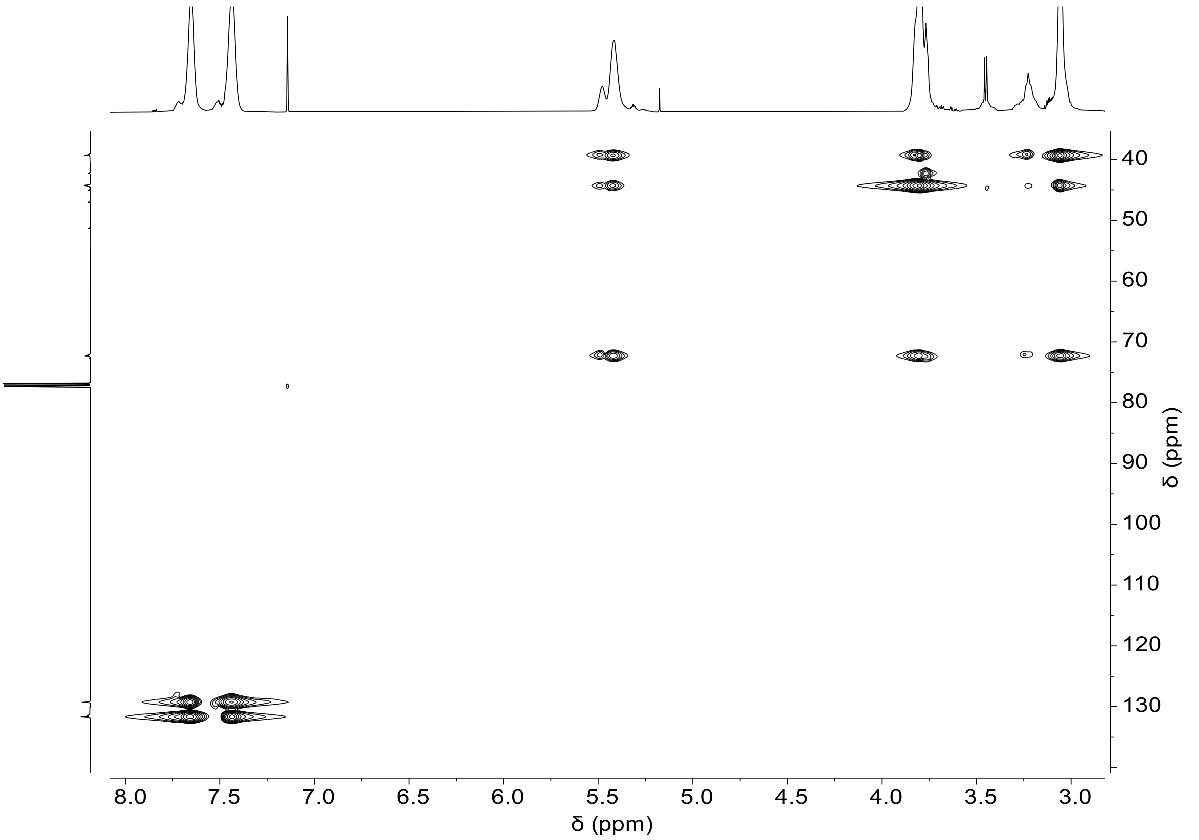

Figure S103: ^1H-^{13}C HSQC NMR spectrum ($CDCl_3$, 25°C) of the precipitated polymer corresponding to ESI table 1 run #15.

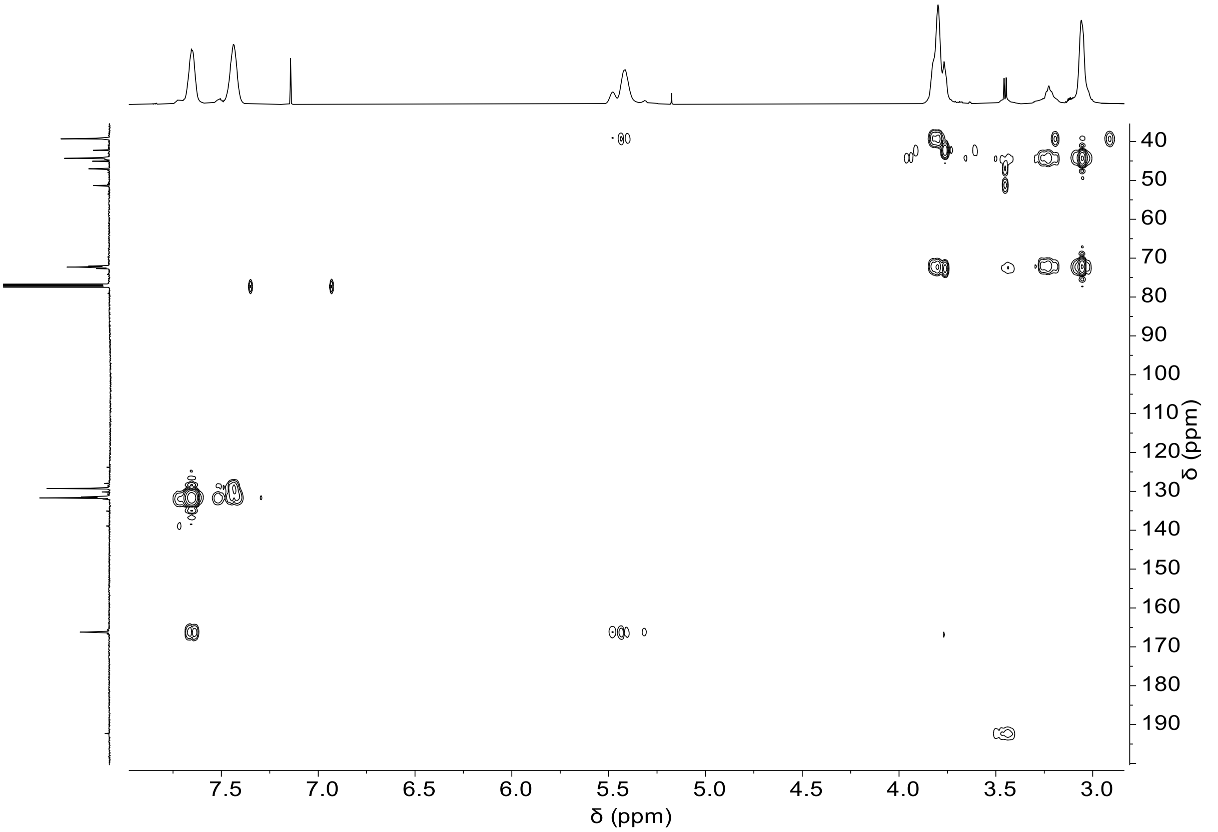

Figure S104: ^1H-^{13}C HMBC NMR spectrum ($CDCl_3$, 25°C) of the precipitated polymer corresponding to ESI table 1 run #15.

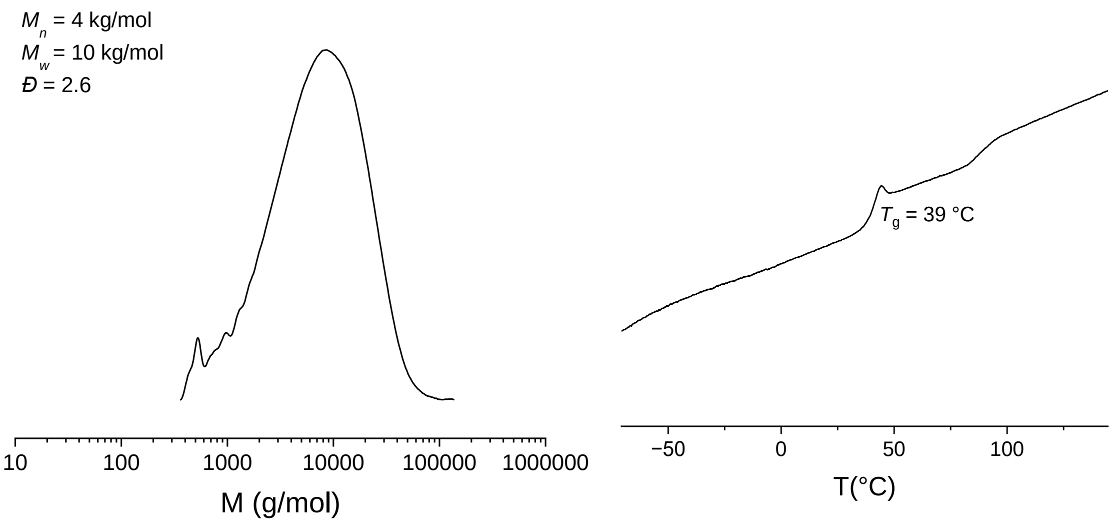

Figure S105: (left) GPC curve and (right) DSC 2nd heating curve of the precipitated polymer corresponding to ESI table 1 run #15.

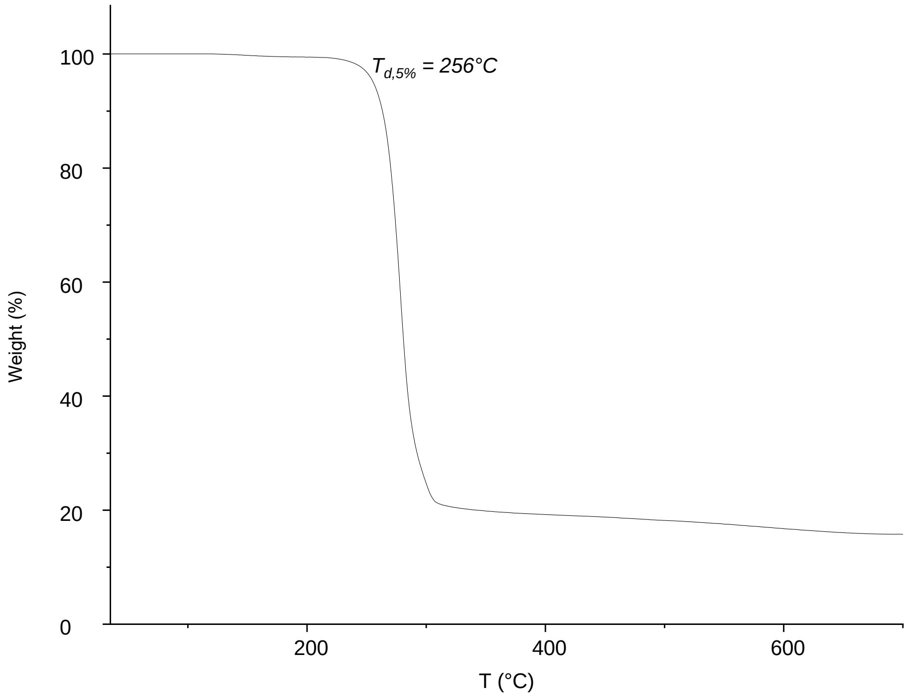

Figure S106: TGA data of the polymer corresponding to ESI table 1 run #15.

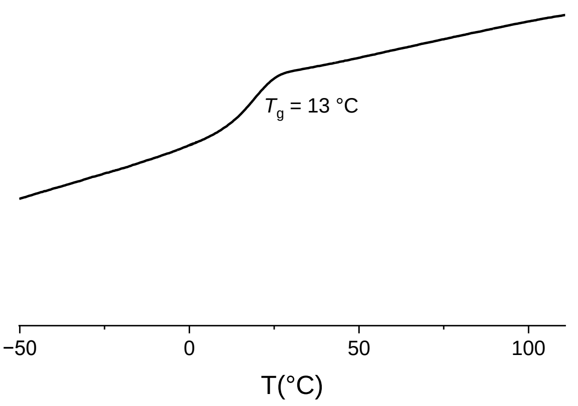

Figure S107: DSC 2nd heating curve of the precipitated polymer corresponding to ESI table 1 run #16.

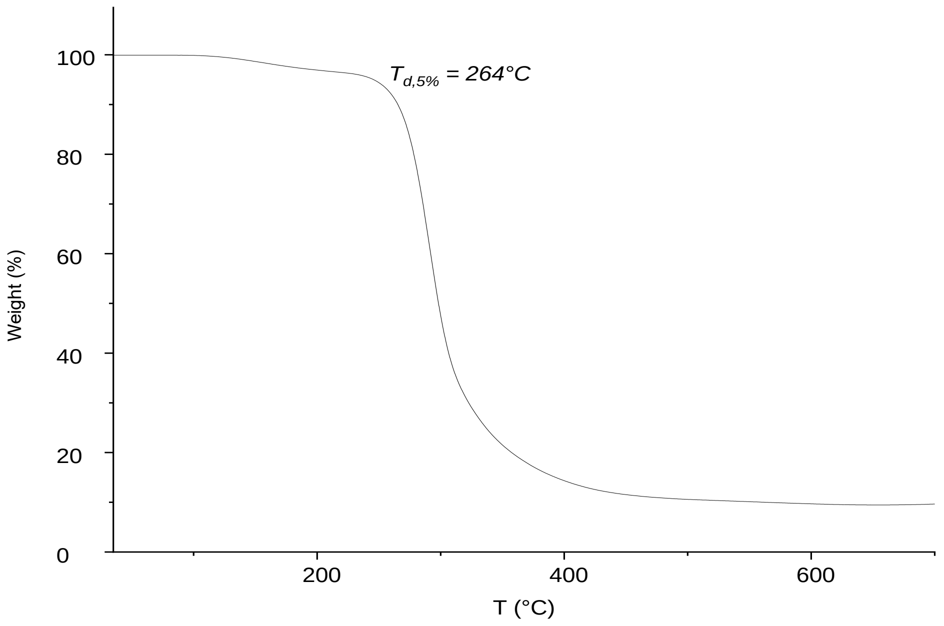

Figure S108: TGA data of the polymer corresponding to ESI table 1 run #16.

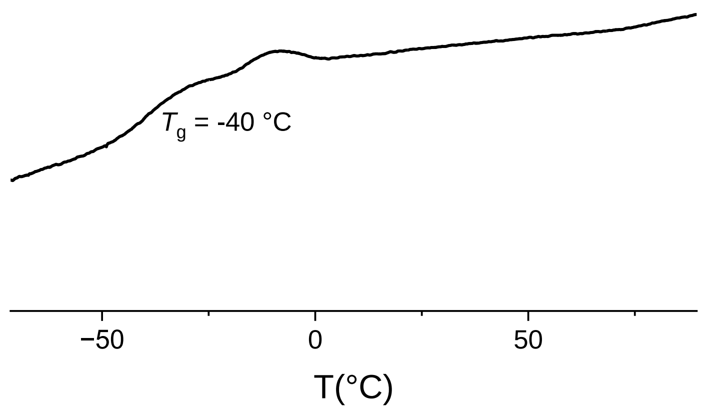

Figure S109: DSC 2nd heating curve of the precipitated polymer corresponding to ESI table 1 run #17.

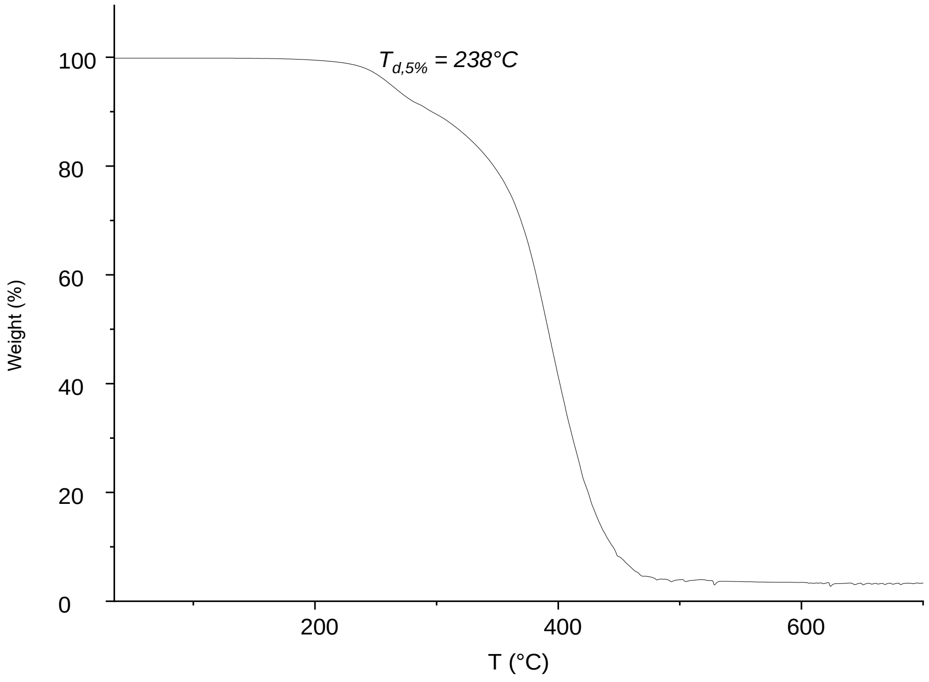

Figure S110: TGA data of the polymer corresponding to ESI table 1 run #17.

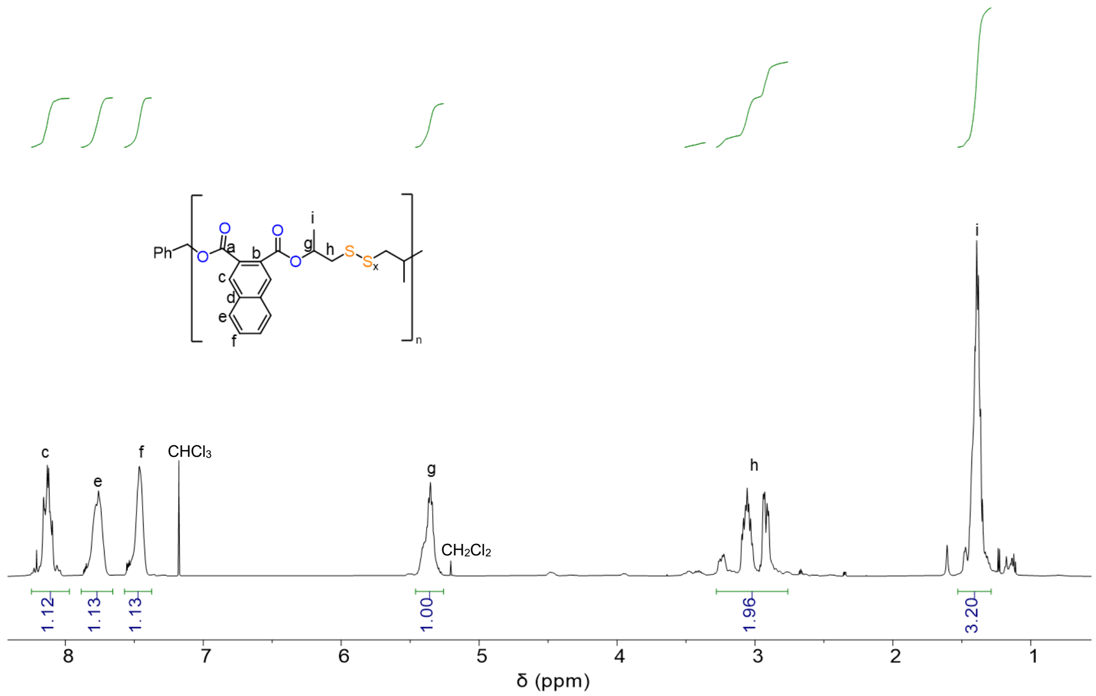

Figure S111: ^{1}H-NMR spectrum (500 MHz, $CDCl_3$, 25°C) of the precipitated polymer corresponding to ESI table 1 run #18.

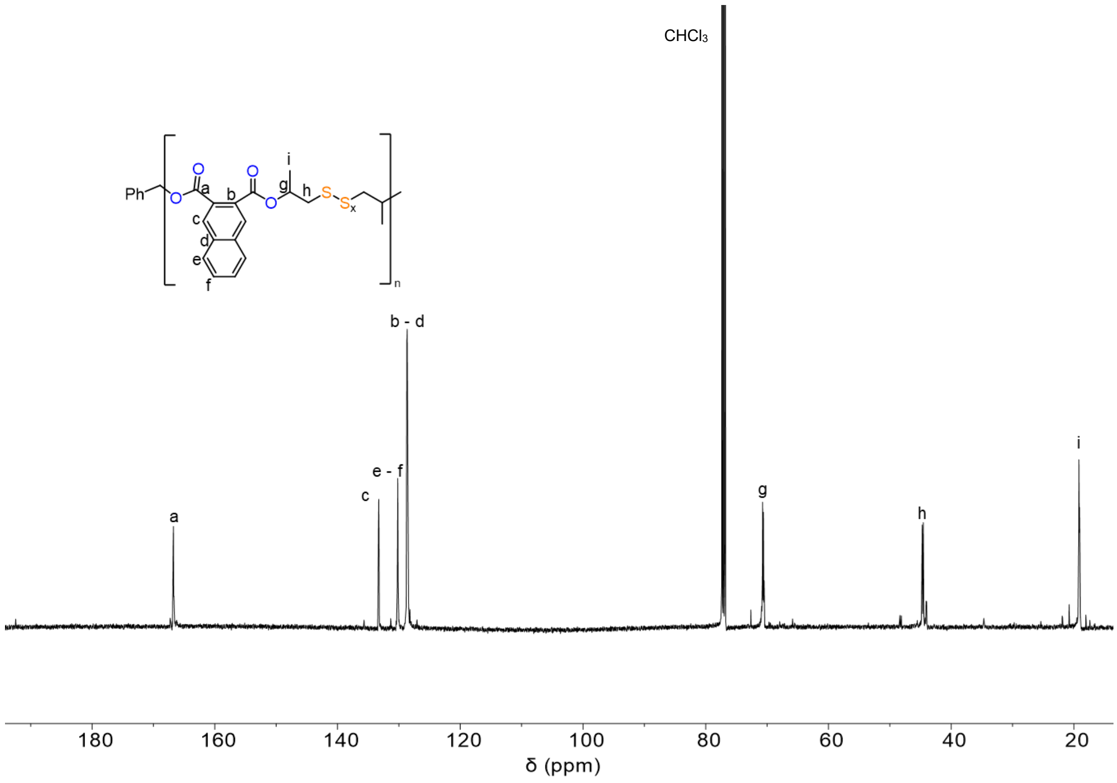

Figure S112: ^{13}C-NMR spectrum (151 MHz, $CDCl_3$, 25°C) of the precipitated polymer corresponding to ESI table 1 run #18.

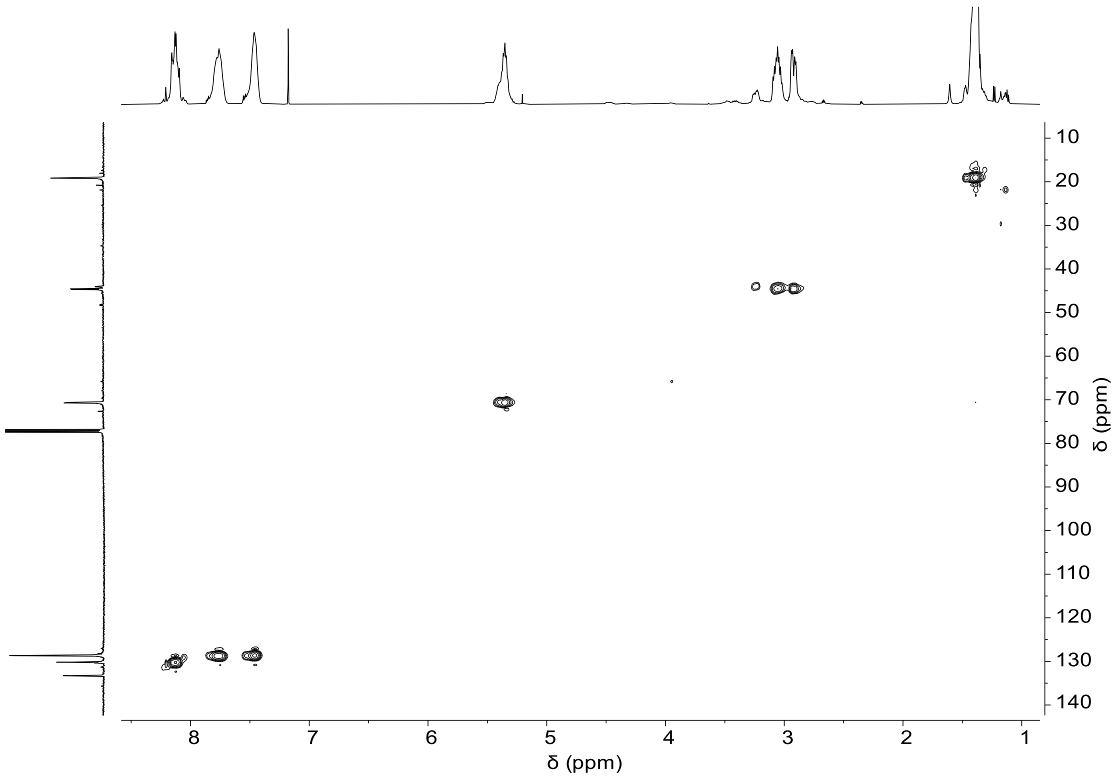

Figure S113: ^{1}H-^{13}C HSQC NMR spectrum ($CDCl_3$, 25°C) of the precipitated polymer corresponding to ESI table 1 run #18.

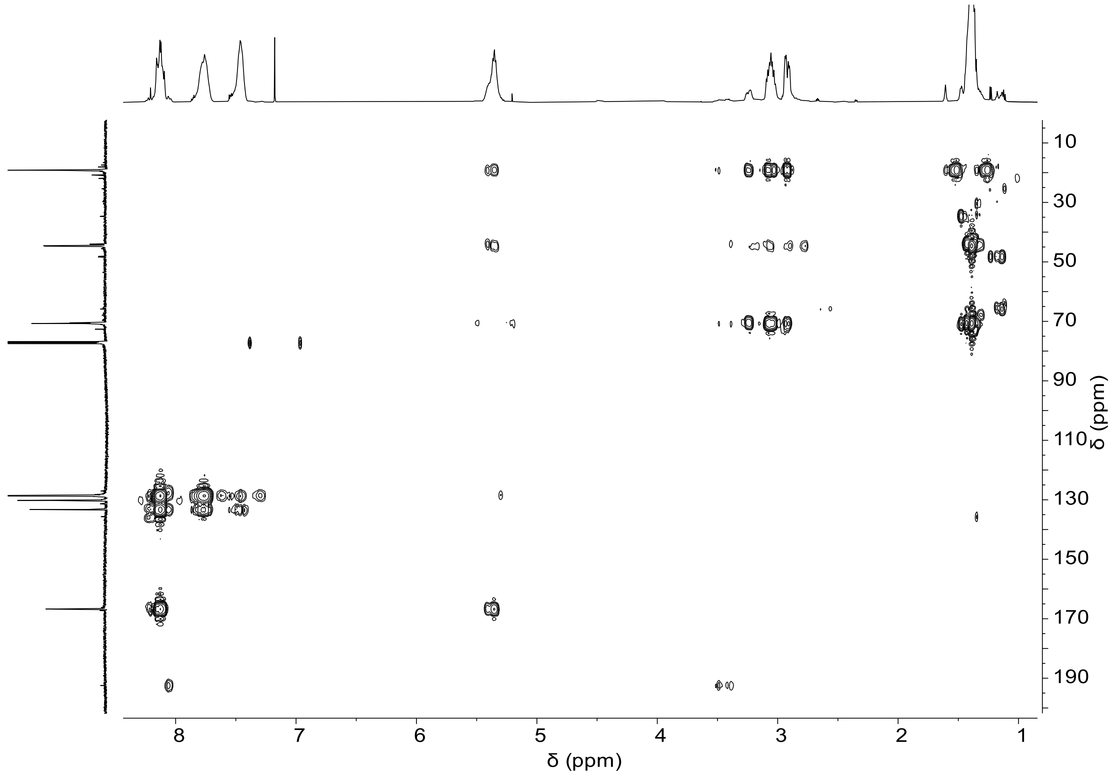

Figure S114: ^{1}H-^{13}C HMBC NMR spectrum ($CDCl_3$, 25°C) of the precipitated polymer corresponding to ESI table 1 run #18.

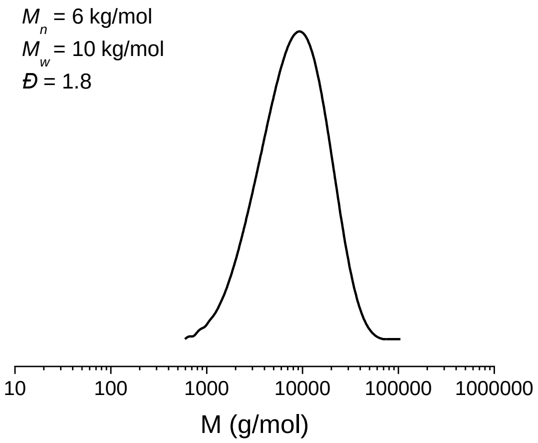

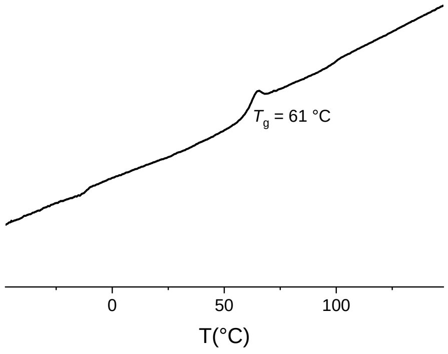

Figure S115: (left) GPC curve and (right) DSC 2nd heating curve of the precipitated polymer corresponding to ESI table 1 run #18.

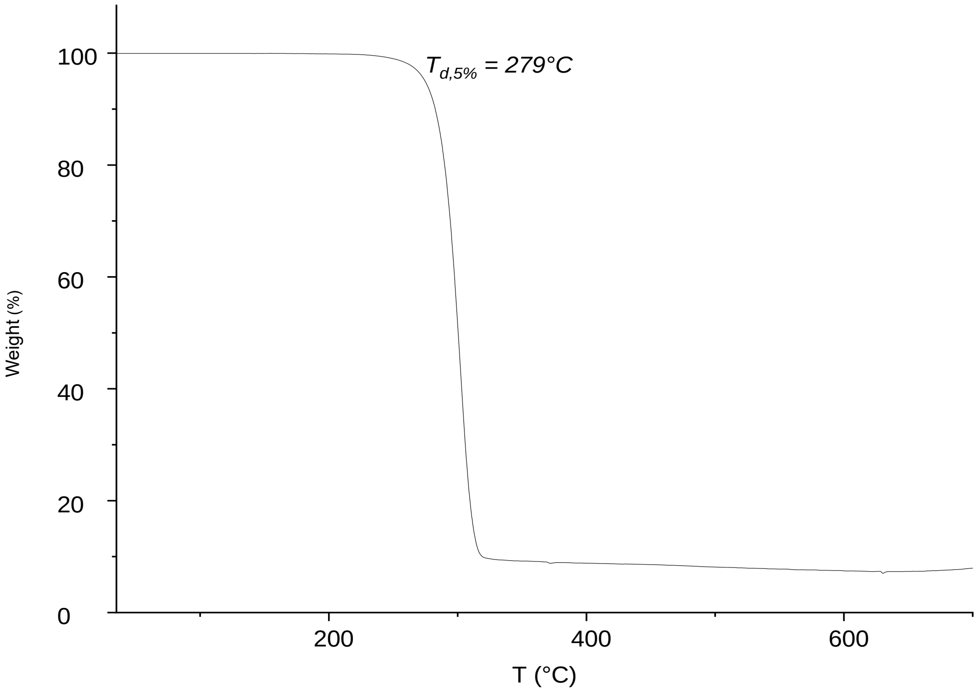

Figure S116: TGA data of the polymer corresponding to ESI table 1 run #18.

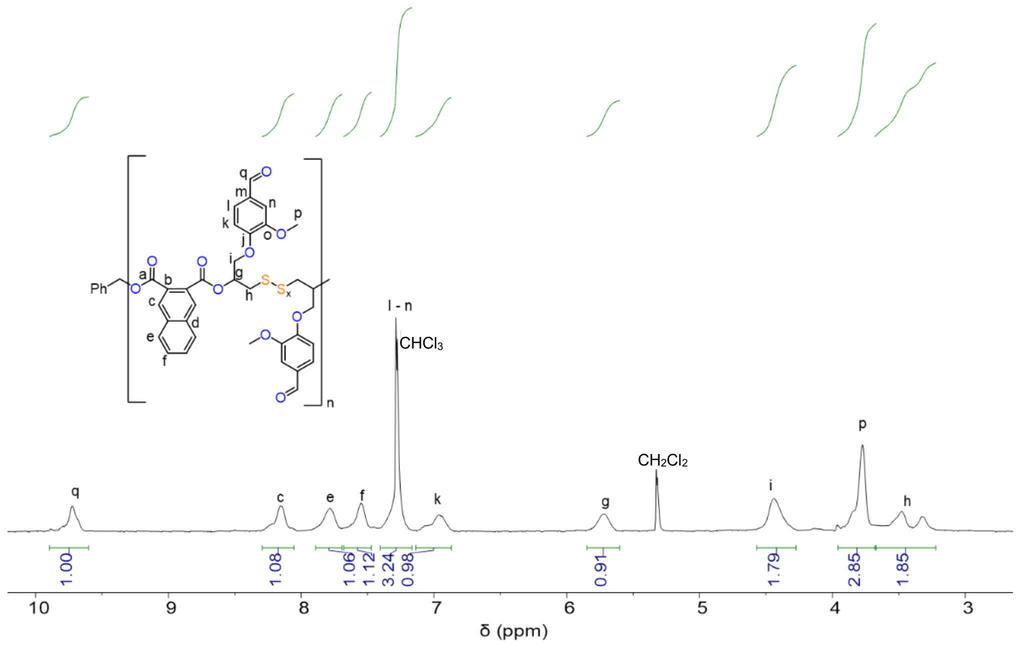

Figure S117: ^{1}H-NMR spectrum (500 MHz, $CDCl_3$, 25°C) of the precipitated polymer corresponding to ESI table 1 run #19.

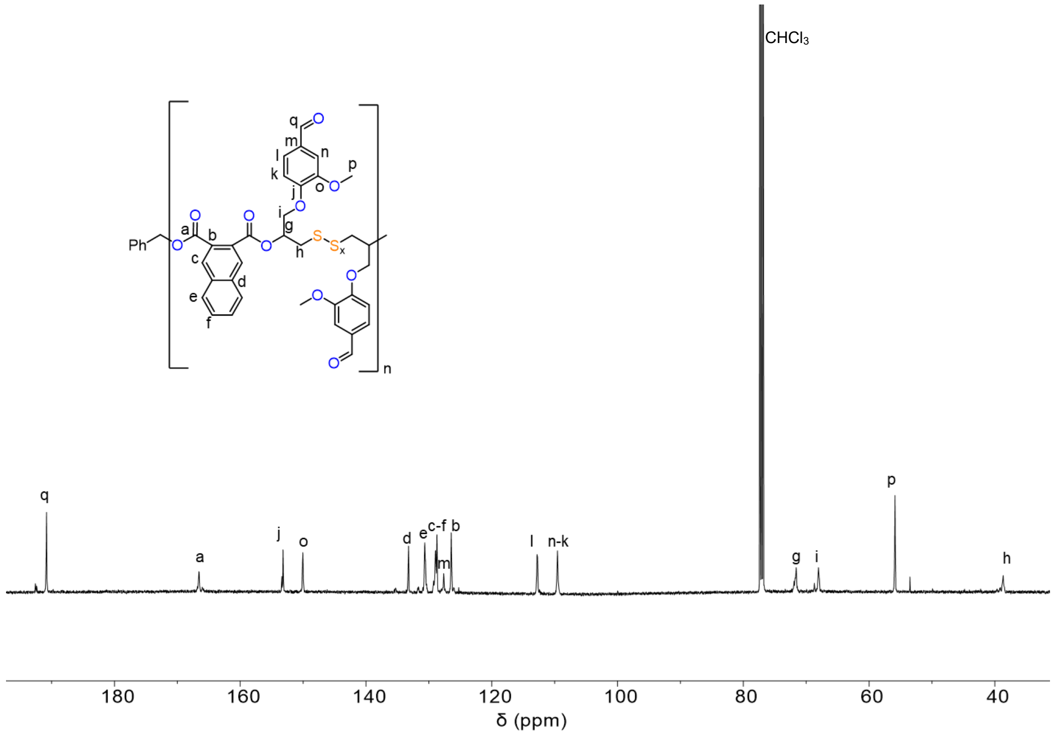

Figure S118: ^{13}C-NMR spectrum (151 MHz, $CDCl_3$, 25°C) of the precipitated polymer corresponding to ESI table 1 run #19.

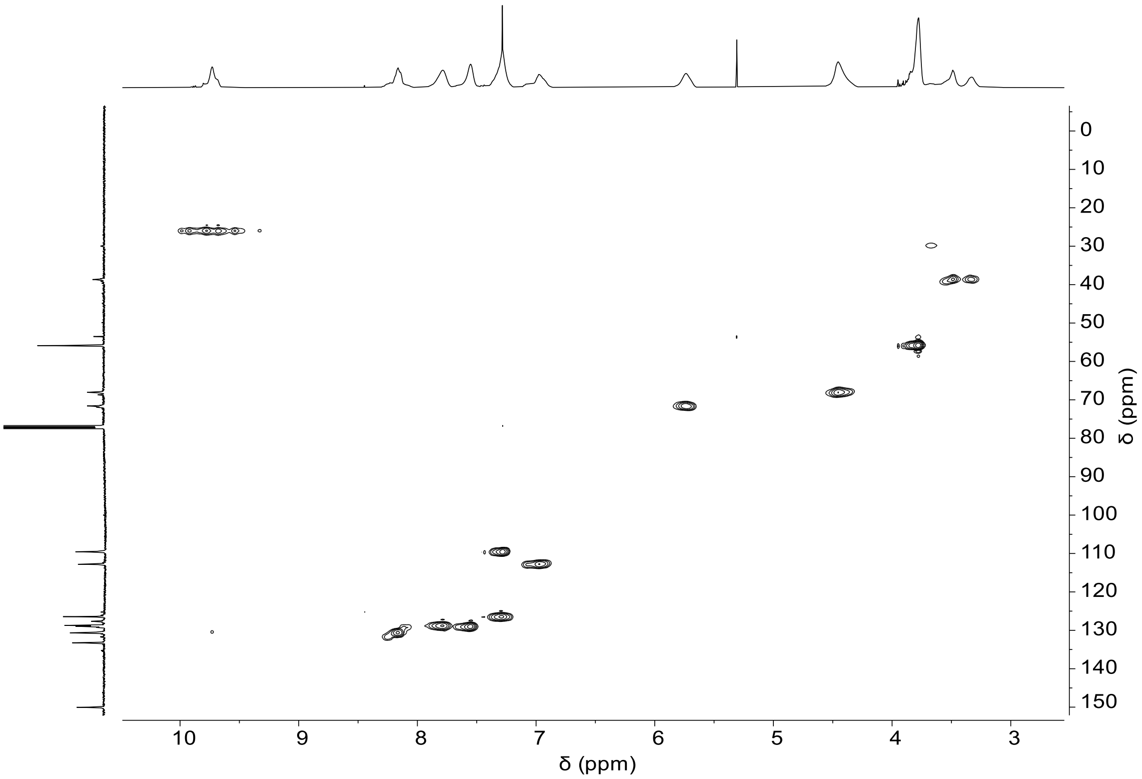

Figure S119: ^{1}H-^{13}C HSQC NMR spectrum ($CDCl_3$, 25°C) of the precipitated polymer corresponding to ESI table 1 run #19.

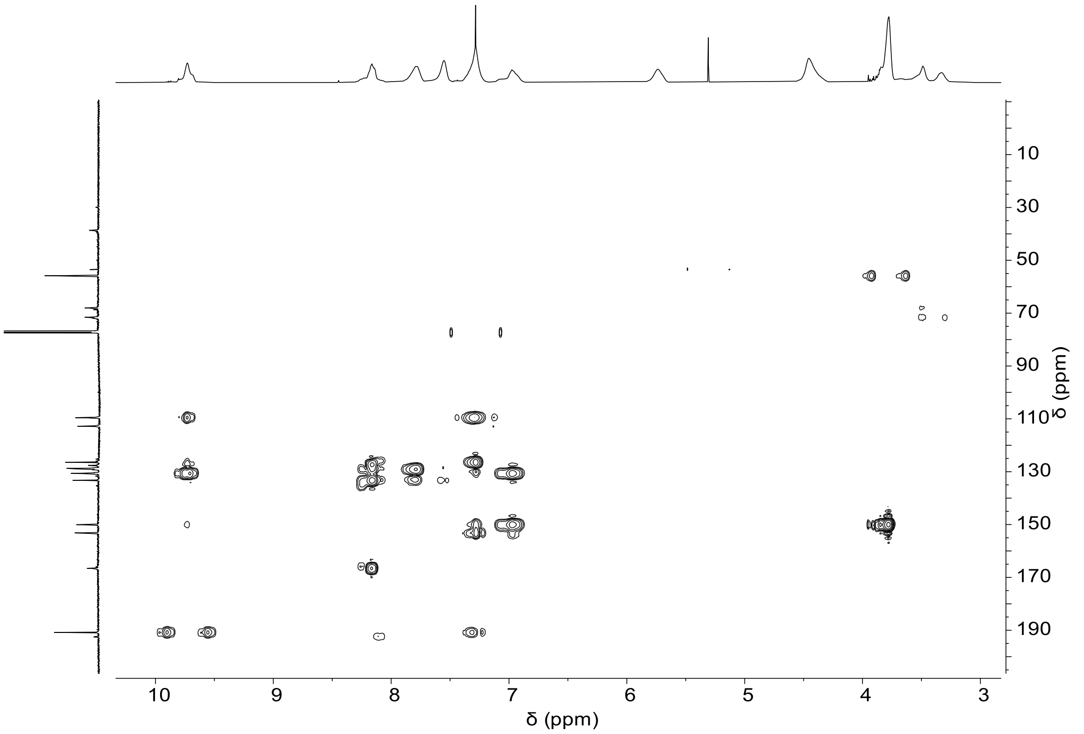

Figure S120: ^{1}H-^{13}C HMBC NMR spectrum ($CDCl_3$, 25°C) of the precipitated polymer corresponding to ESI table 1 run #19.

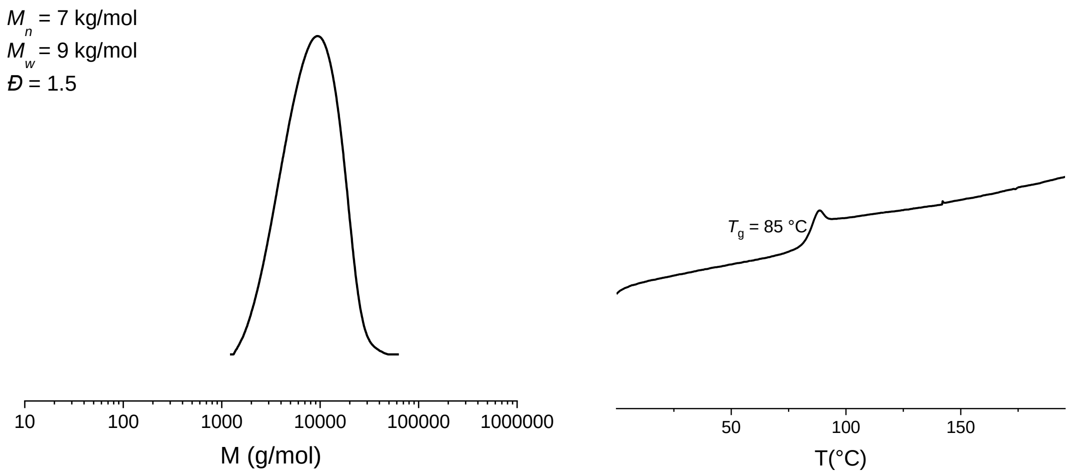

Figure S121: (left) GPC curve and (right) DSC 2nd heating curve of the precipitated polymer corresponding to ESI table 1 run #19.

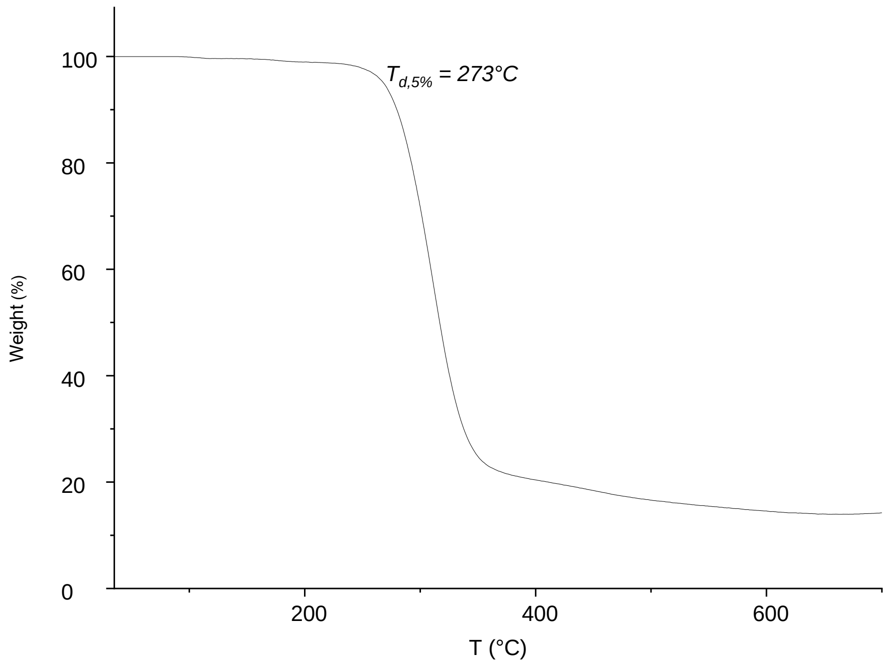

Figure S122: TGA data of the polymer corresponding to ESI table 1 run #19.

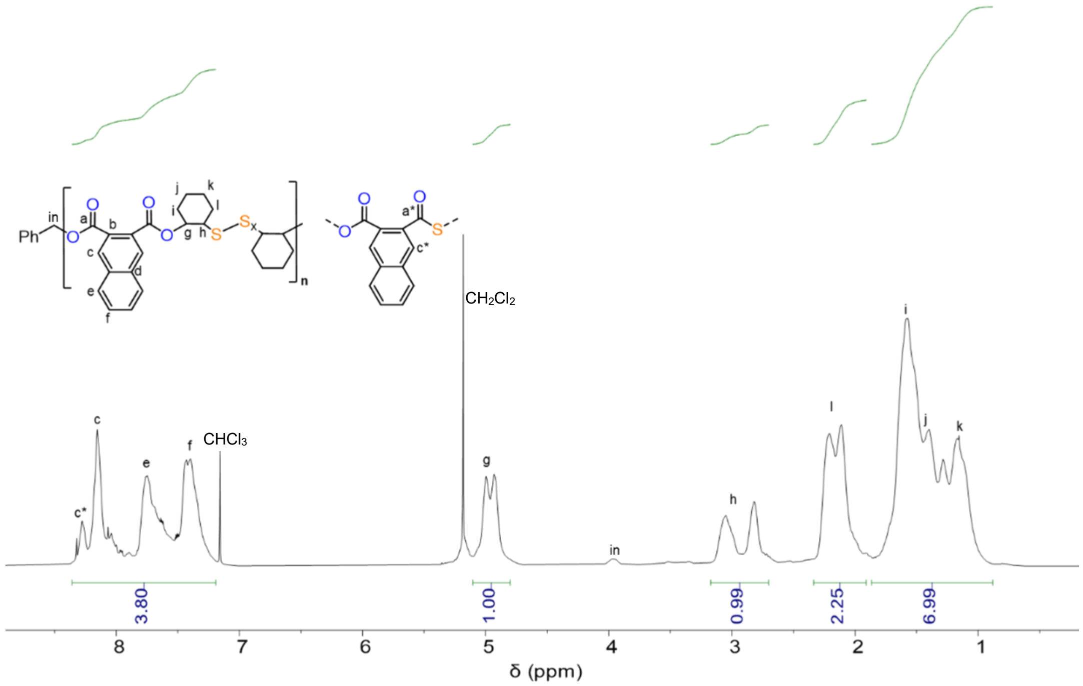

Figure S123: ^{1}H-NMR spectrum (500 MHz, $CDCl_3$, 25°C) of the precipitated polymer corresponding to ESI table 1 run #20.

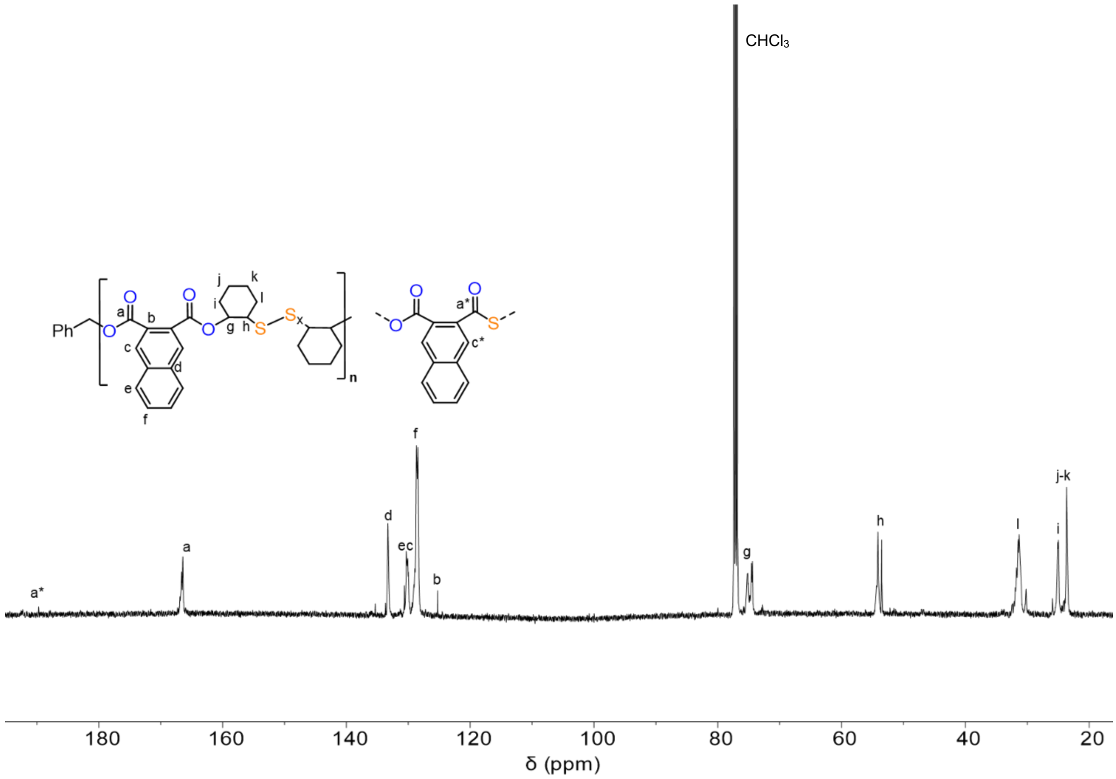

Figure S124: ^{13}C-NMR spectrum (151 MHz, $CDCl_3$, 25°C) of the precipitated polymer corresponding to ESI table 1 run #20.

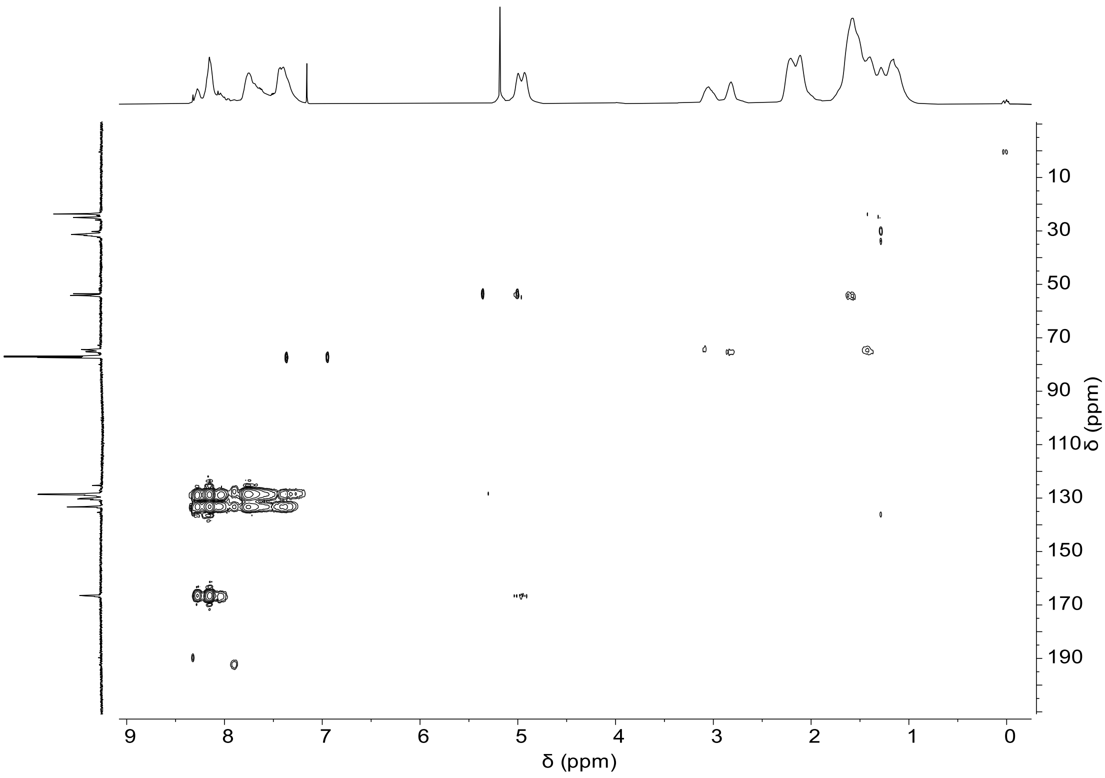

Figure S125: ^1H-^{13}C HSQC NMR spectrum ($CDCl_3$, 25°C) of the precipitated polymer corresponding to ESI table 1 run #20.

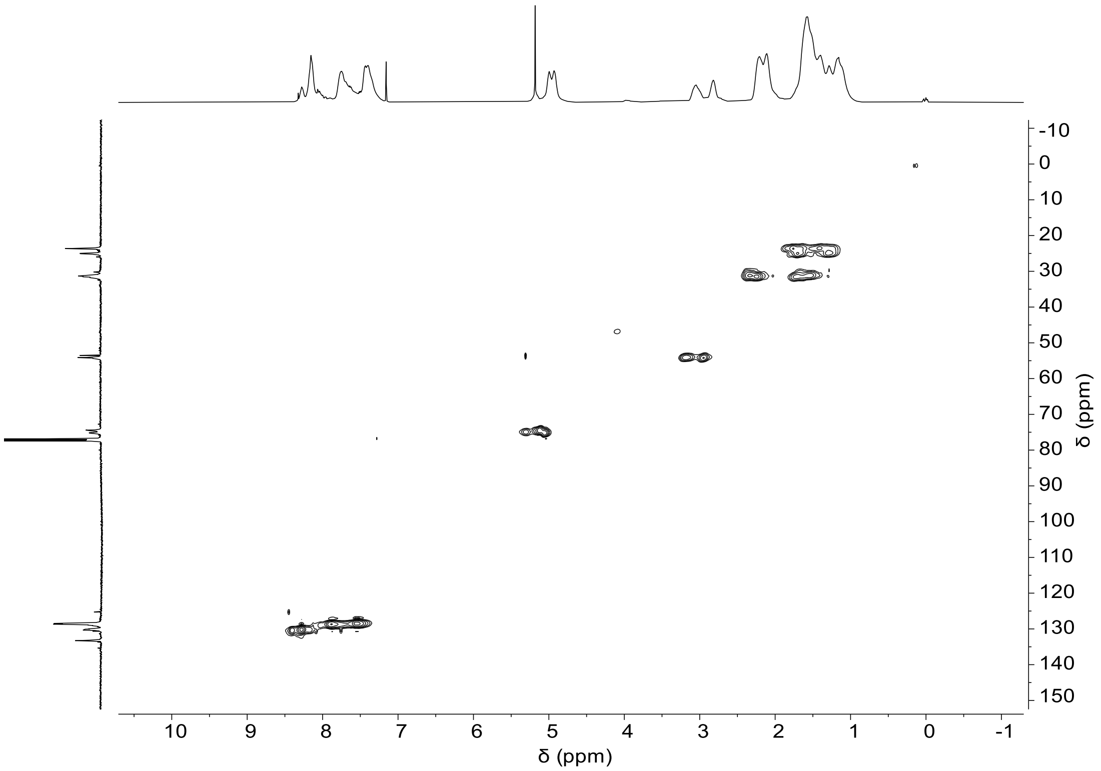

Figure S126: ^1H-^{13}C HMBC NMR spectrum ($CDCl_3$, 25°C) of the precipitated polymer corresponding to ESI table 1 run #20.

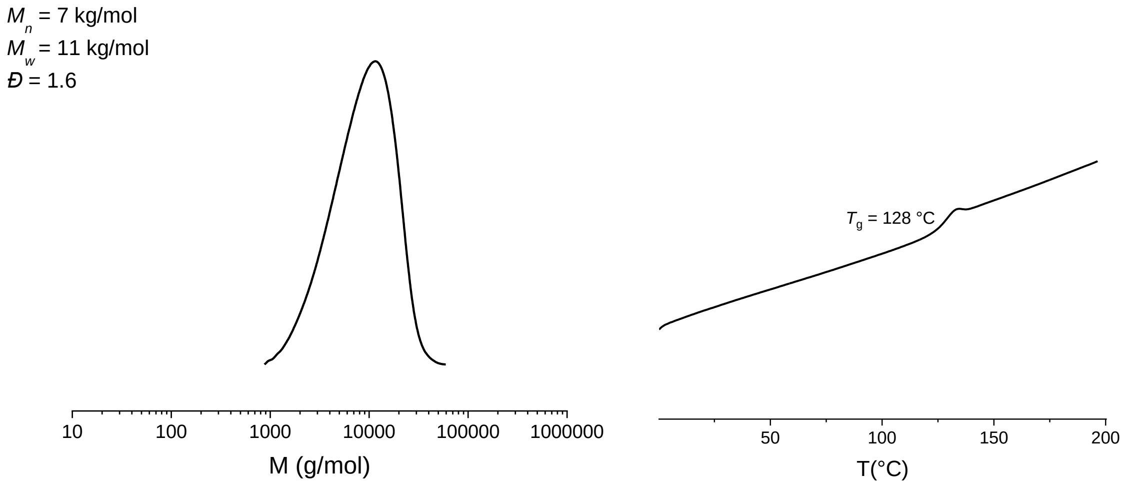

Figure S127: (left) GPC curve and (right) DSC 2nd heating curve of the precipitated polymer corresponding to ESI table 1 run #20.

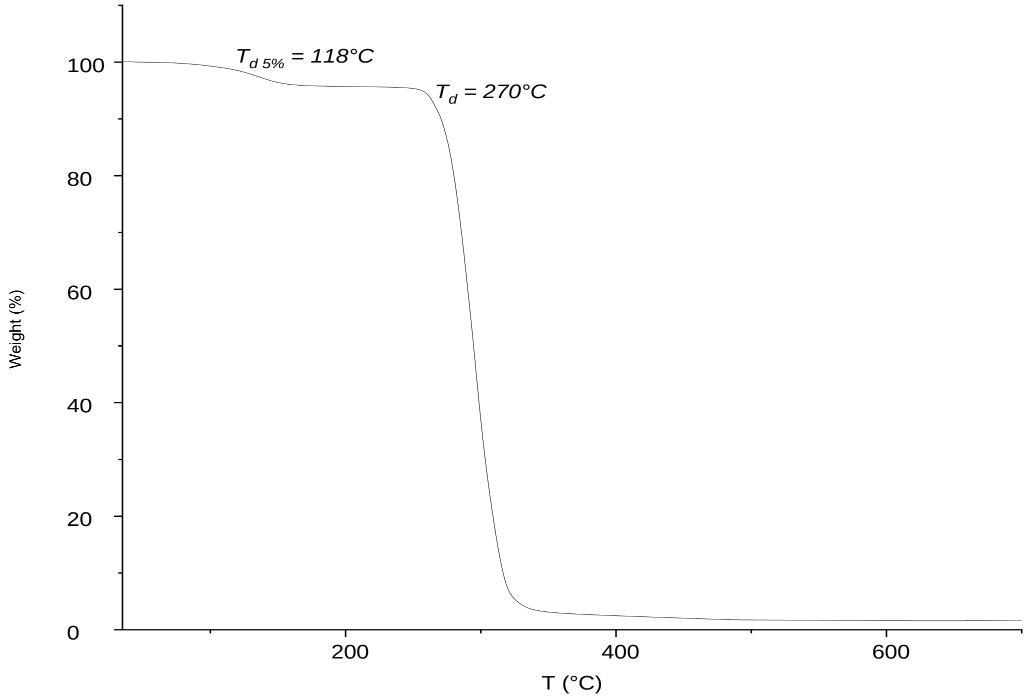

Figure S 128: TGA data of the polymer corresponding to ESI table 1 run #20.

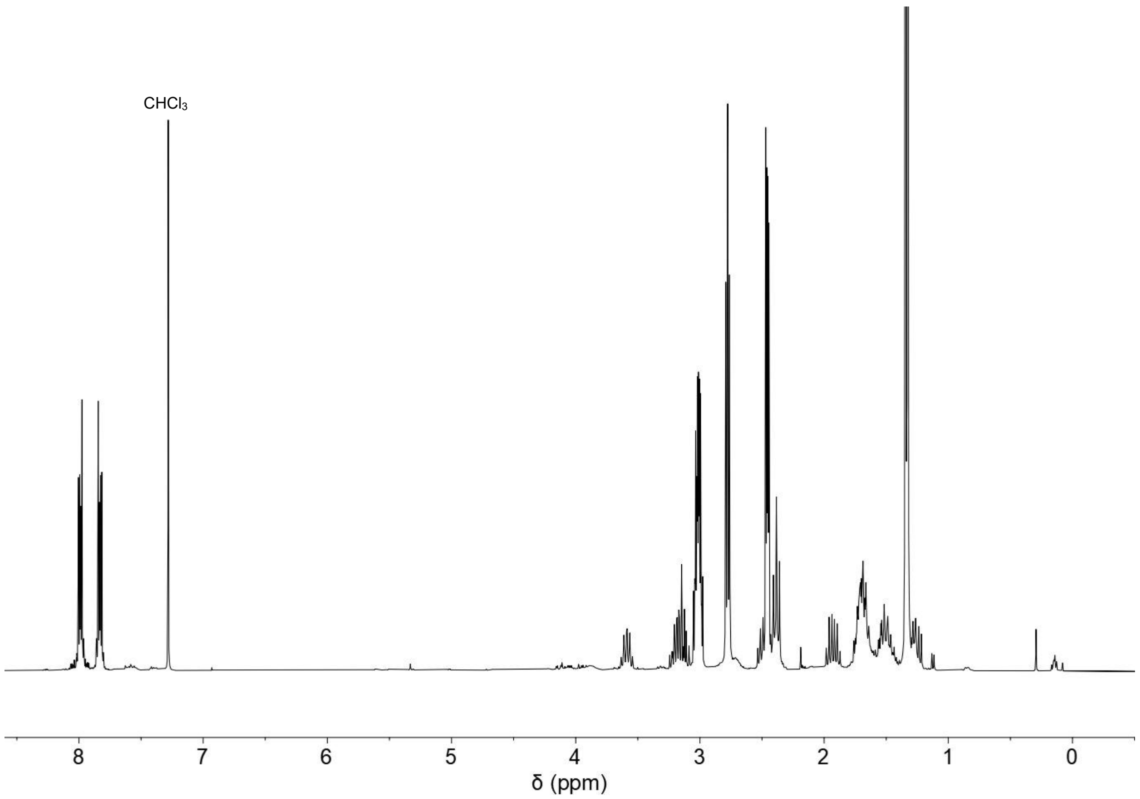

Figure S129: ^{1}H-NMR spectrum (500 MHz, $CDCl_3$, 25°C) of the crude mixture of S:lipoic acid:PTA:PO tetrapolymerization (1 eq. LiOBn: 200 eq. S : 200 eq. lipoic acid : 200 eq. PTA: 500 eq. epoxide).

Section S5: Post functionalization

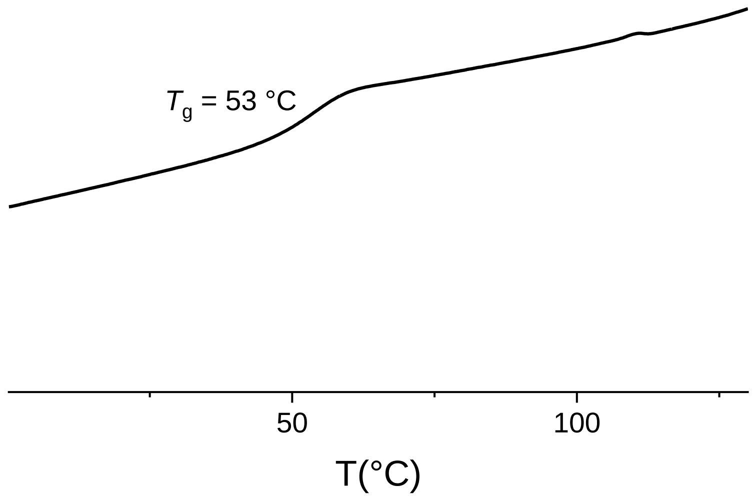

Figure S130: DSC 2nd heating curve of the imine cross-linked polymer.

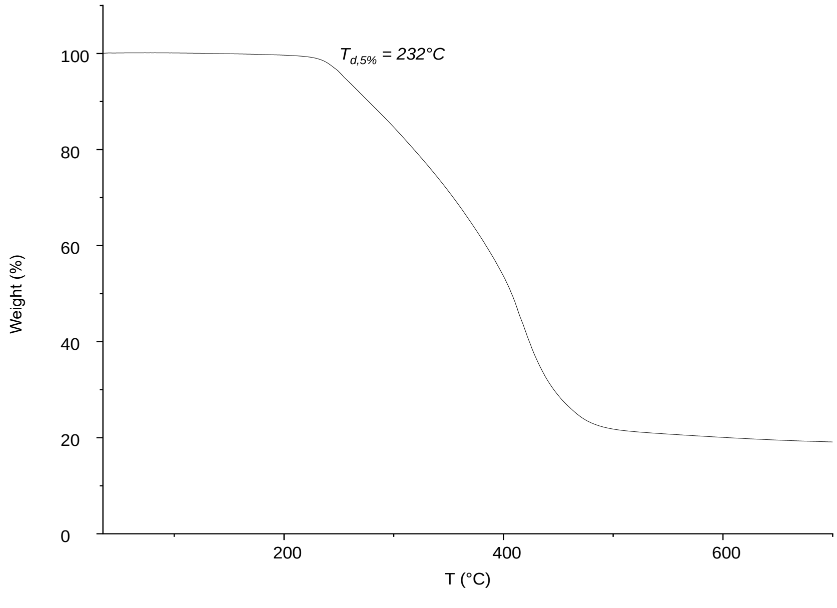

Figure S131: TGA data of the imine cross-linked polymer.

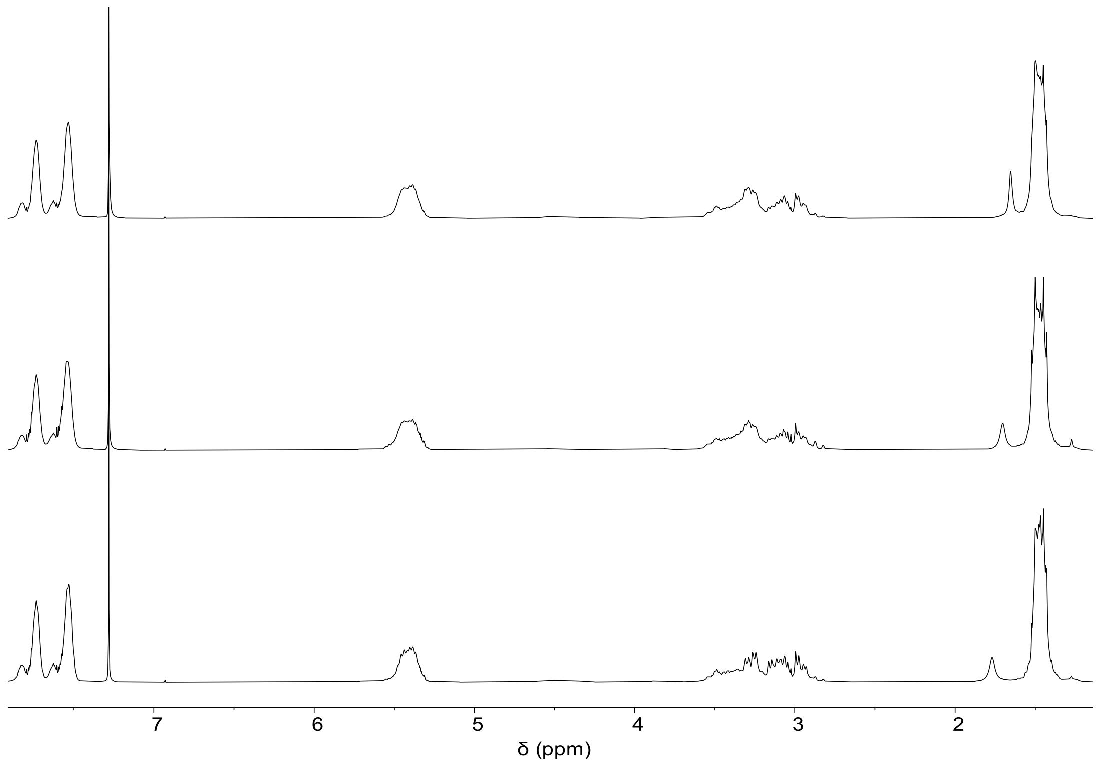

Figure S132: ^{1}H-NMR spectra (500 MHz, $CDCl_3$, 25°C) of the precipitated polymers corresponding to table 1 run #9 and the same polymer after insertion of S_8. Bottom to top: table 1 run #9 (0% S_8 inserted), 5% S_8 w/w, 10% w/w S_8.

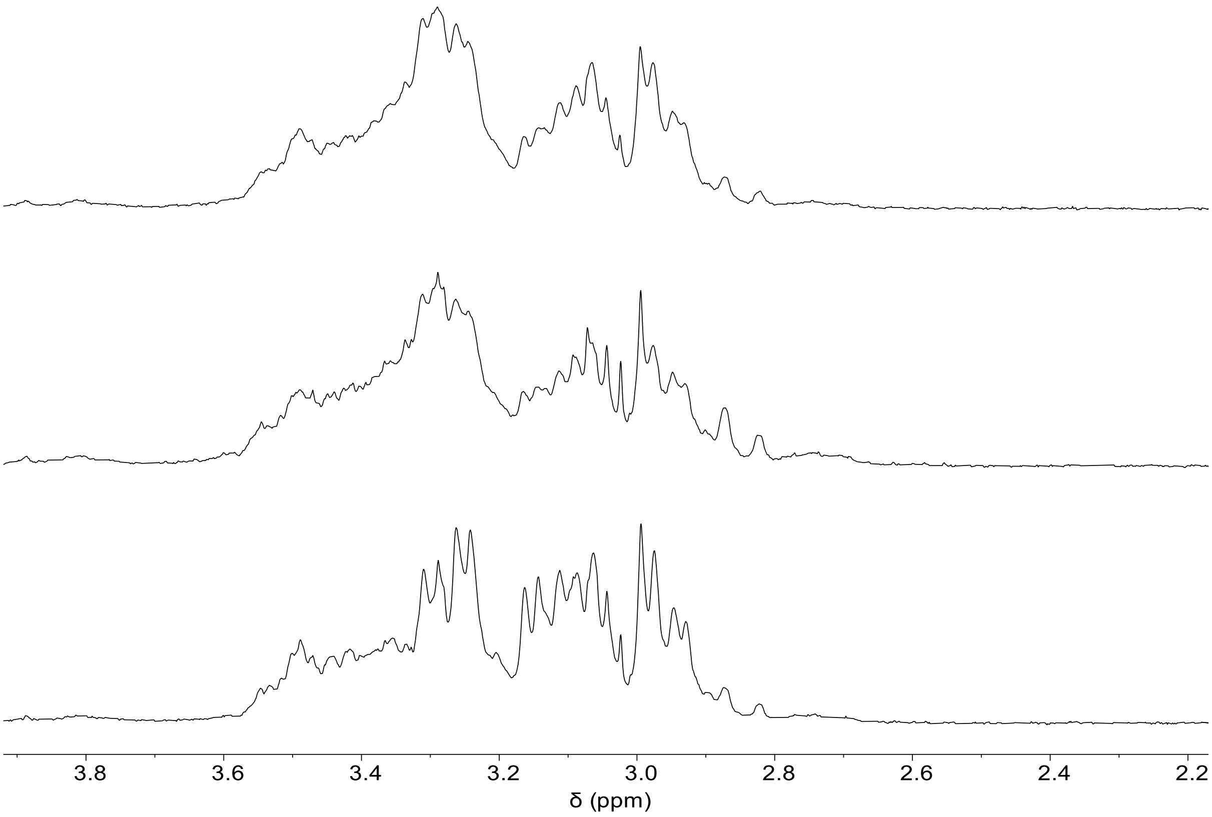

Figure S133: ^{1}H-NMR spectra (500 MHz, $CDCl_3$, 25°C) of the precipitated polymers corresponding to table 1 run #9 and the same polymer after insertion of S_8. Zoomed in into the polysulfide region.

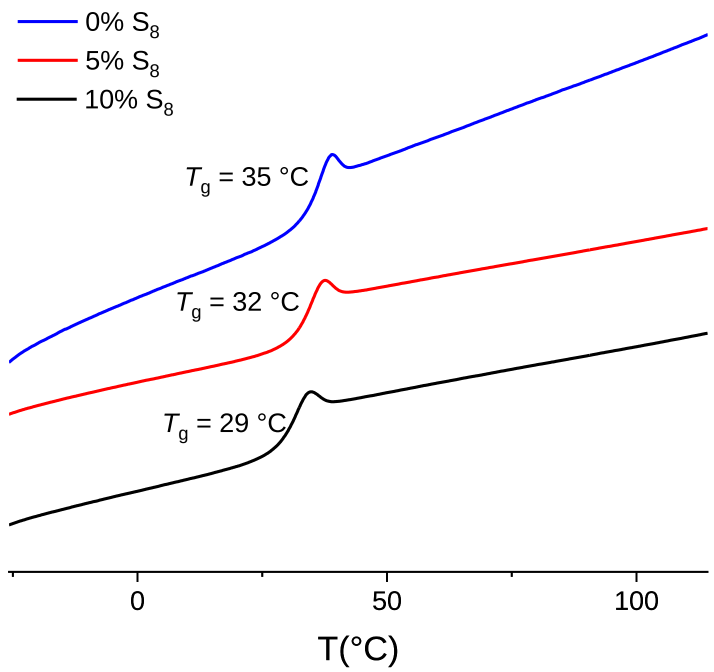

Figure S134: DSC 2nd heating curve of the precipitated polymer corresponding to table 1 run #9 and the same polymer after insertion of S_8.

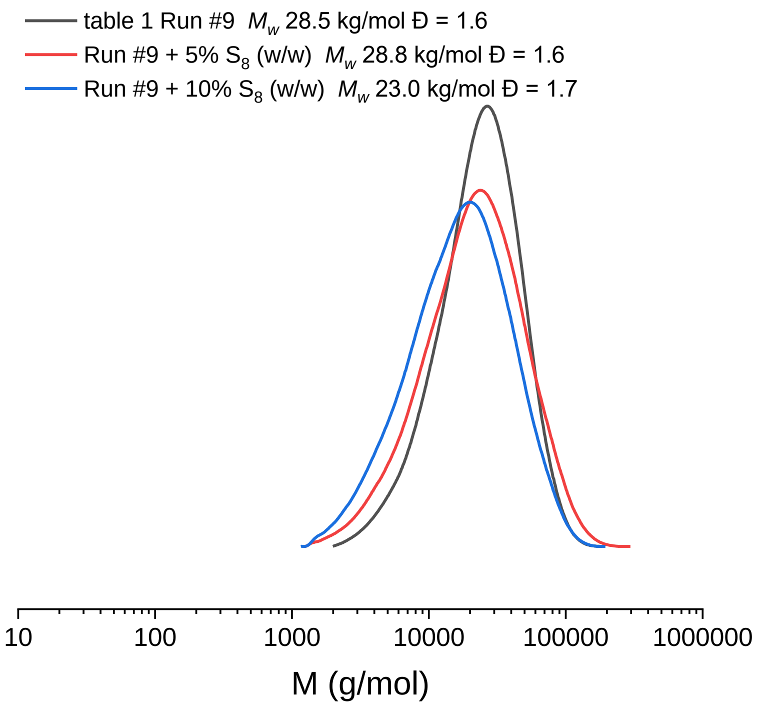

Figure S135: GPC curve of the precipitated polymer corresponding to table 1 run #9 and comparison with 2 batches of the same polymer, one after addition of 5% S_8 (w/w) and the other after addition of 10% S_8 (w/w).

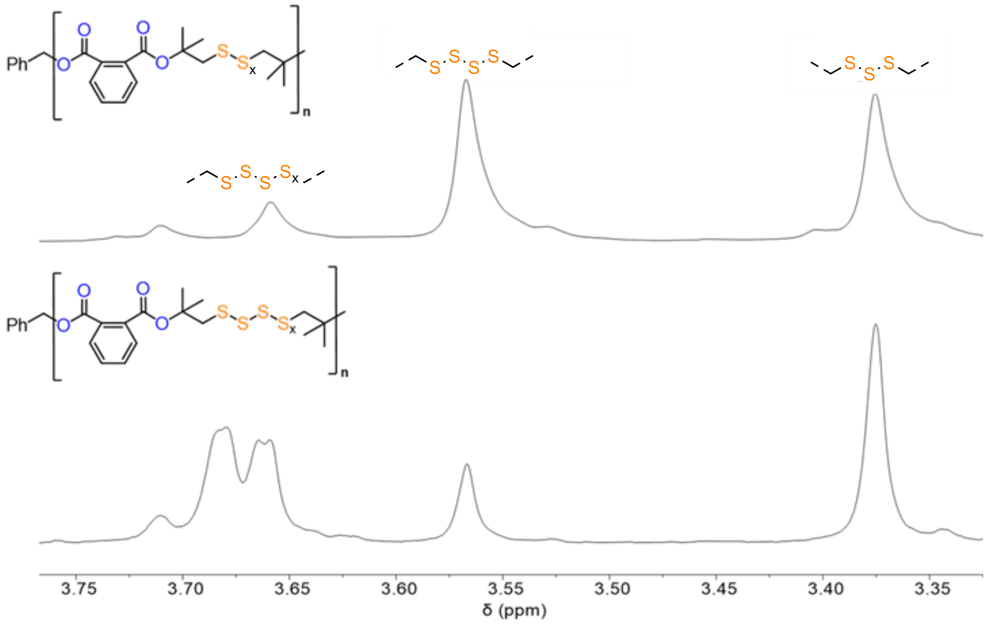

Figure S136: ^{1}H-NMR spectra (500 MHz, $CDCl_3$, 25°C) of the precipitated polymer corresponding to ESI table 1 run #15 (top) and the same polymer after insertion of S_8 (10% w/w) (bottom), zoomed in into the polysulfide region.

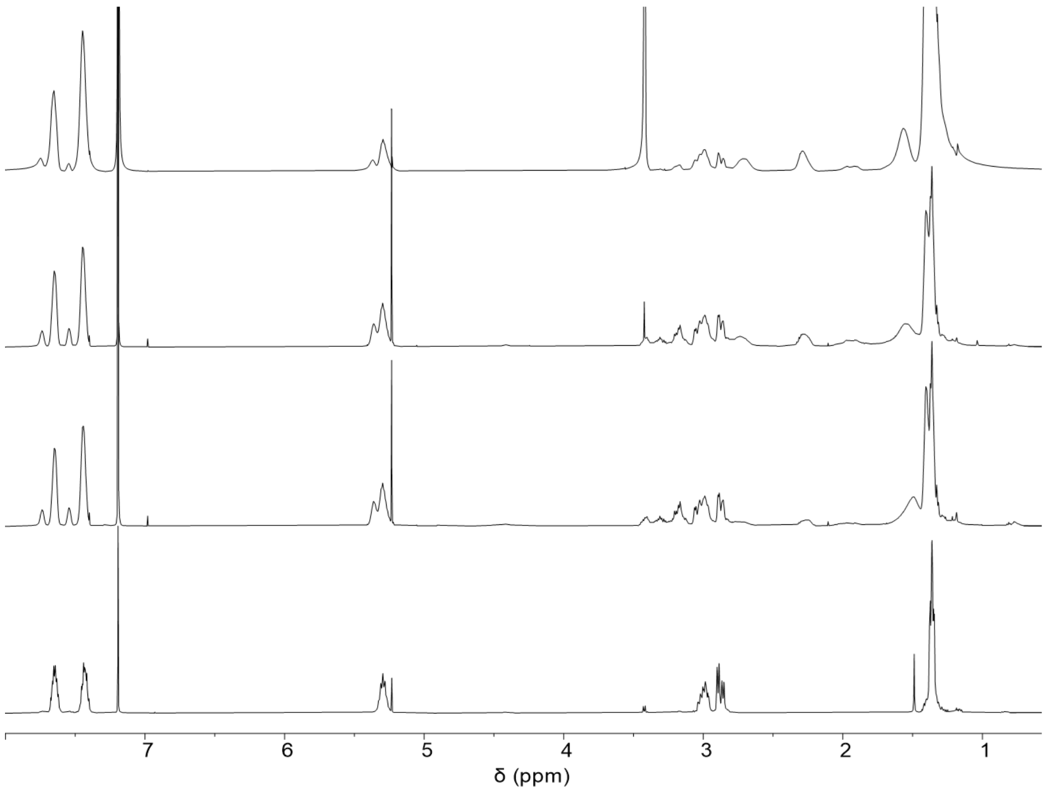

Figure S137: ^{1}H-NMR spectra (500 MHz, $CDCl_3$, 25°C) of the precipitated polymers from the lipoic acid insertion experiment. Respectively from bottom to top: linear polymer from table 1 run #9, 2:1 (weight ratio in starting mixture) pol:LA, 1:1 pol:LA, 1:2 pol:LA.

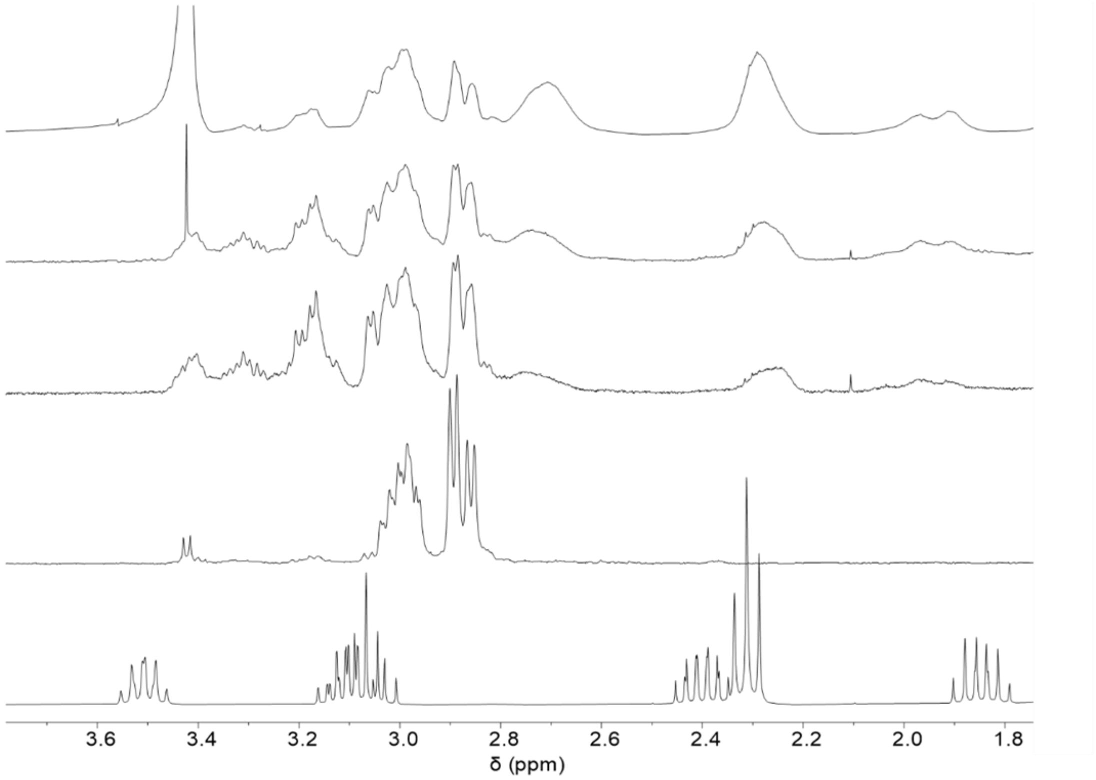

Figure S138: ^{1}H-NMR spectra (500 MHz, $CDCl_3$, 25°C) of the precipitated polymers from the lipoic acid insertion experiment. Zoomed in into the polysulfide region. Respectively from bottom to top: α-lipoic acid, linear polymer from table 1 run #9, 2:1 (weight ratio in starting mixture) pol:LA, 1:1 pol:LA, 1:2 pol:LA.

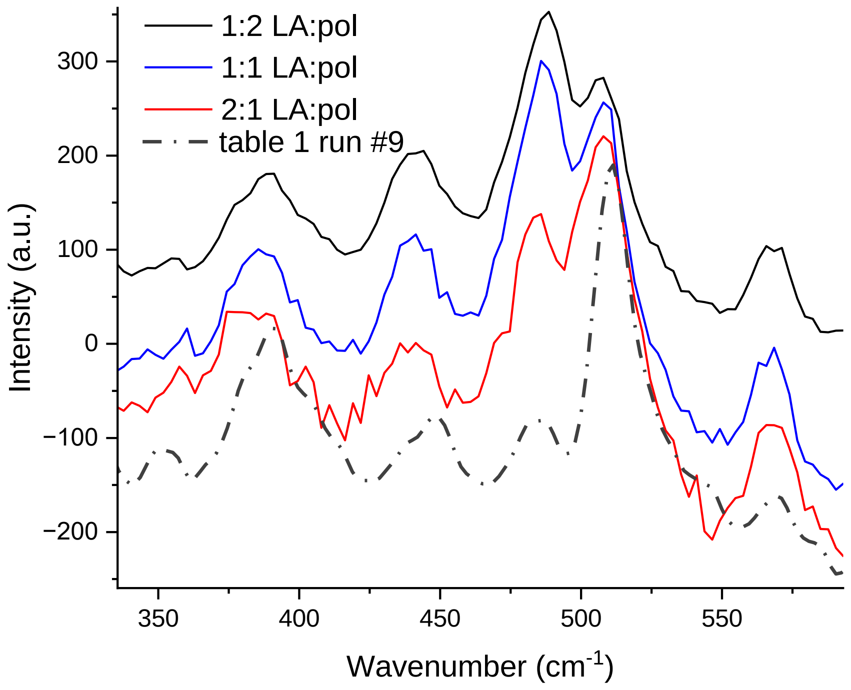

Figure S139: Zoom into the C-S_x-C stretching vibrations of the Raman spectrum of the polymer corresponding to table 1 run # 9 after the insertion of different amount of lipoic acid.

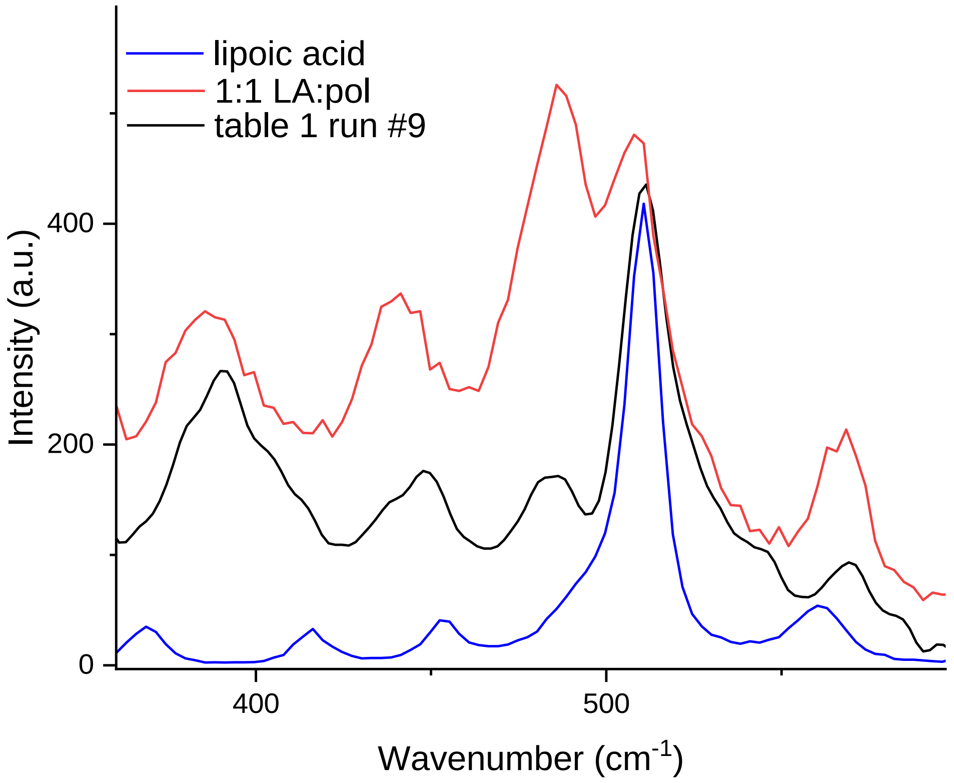

Figure S140: Zoom into the C-S_x-C stretching vibrations of the Raman spectrum of the polymer corresponding to table 1 run # 9, lipoic acid and the 1:1 mixture after insertion.

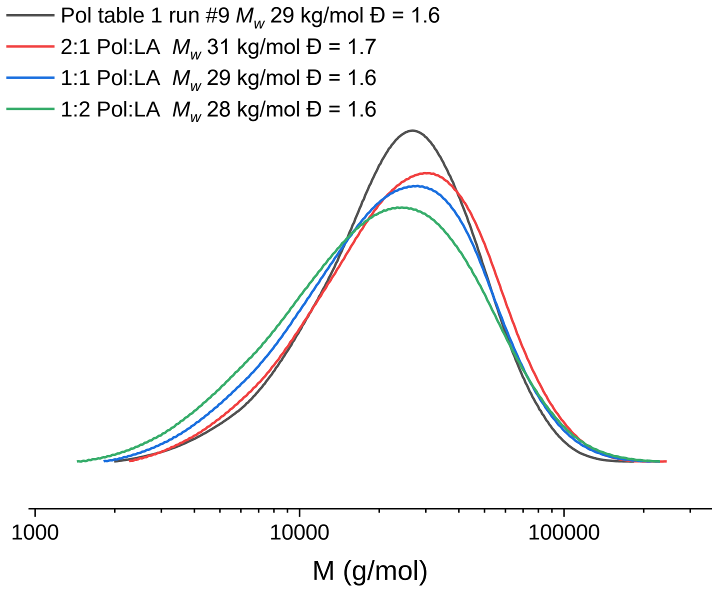

Figure S141: GPC curve of the precipitated polymer corresponding to table 1 run #9 and the polymers obtained from the lipoic acid insertion.

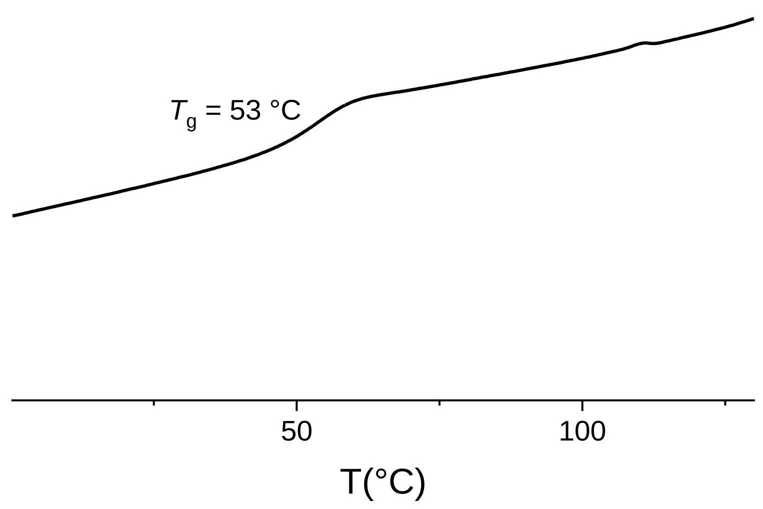

Figure S142: DSC 2nd heating curve of the imine cross-linked polymer after lipoic acid insertion.

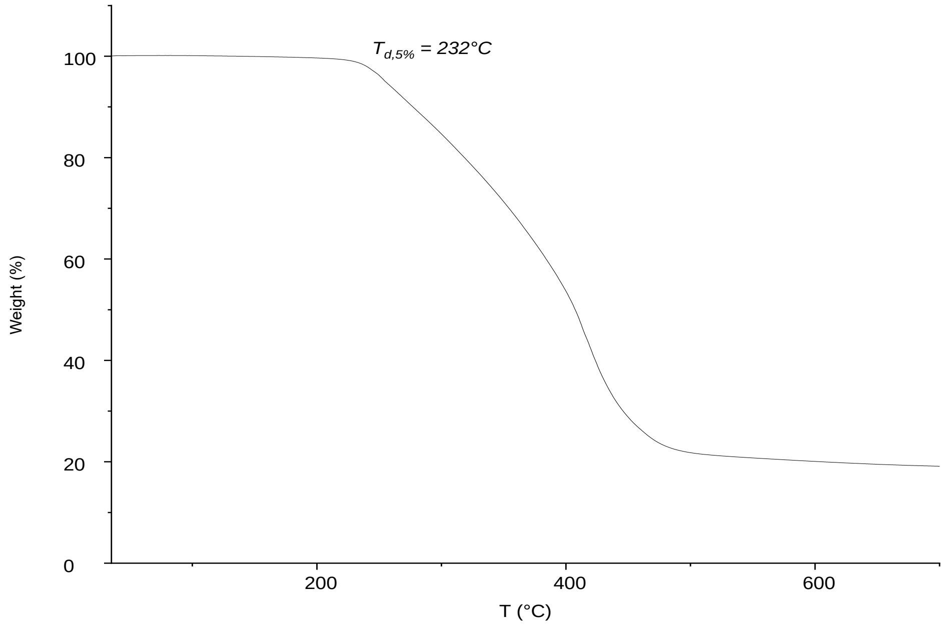

Figure S143: TGA data of the imine cross-linked polymer after lipoic acid insertion.

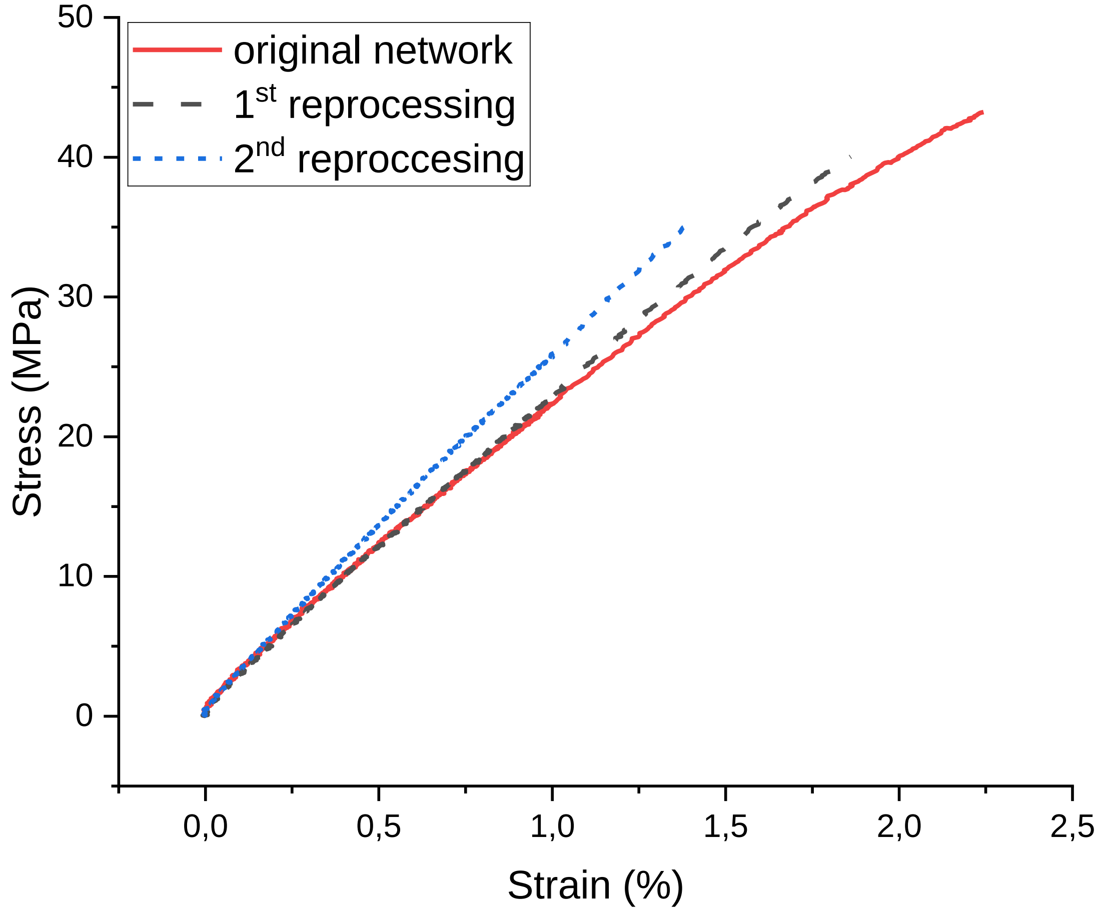

Figure S144: Evaluation of mechanical properties of the imine cross-linked material after reprocessing.

	E_γ [Gpa]	σ [Mpa]	ε [%]
Virgin material	2.337 ±0.096	49.93 ± 4.6	2.73 ± 0.34
1st reprocessing	2.450 ± 0.113	43.98 ± 10.6	2.12 ± 0.83
2nd reprocessing	2.521 ± 0.105	30.41 ± 7.1	1.31 ±0.24

Table S2: Results from the tensile test strength of dog bones cut from cross linked networks. Average values and associated errors are calculated from 3 samples.

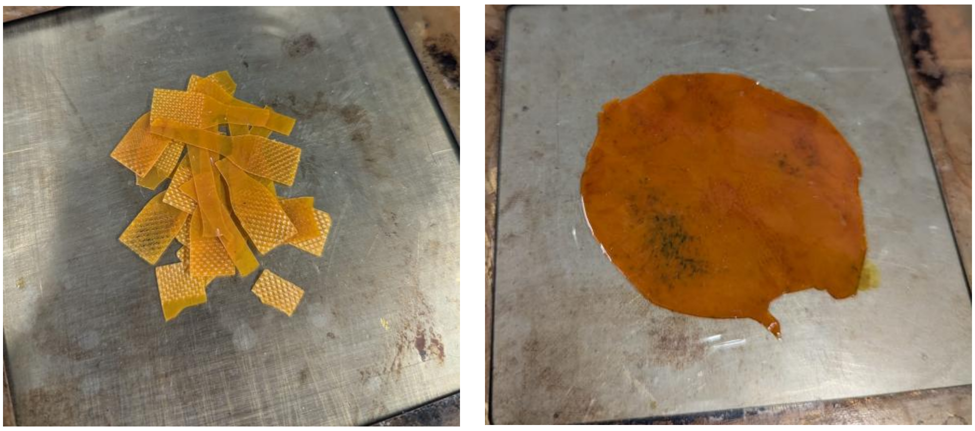

Figure S145: Before and after reprocessing for 1 h at 130°C.

1 h

12 h

48 h

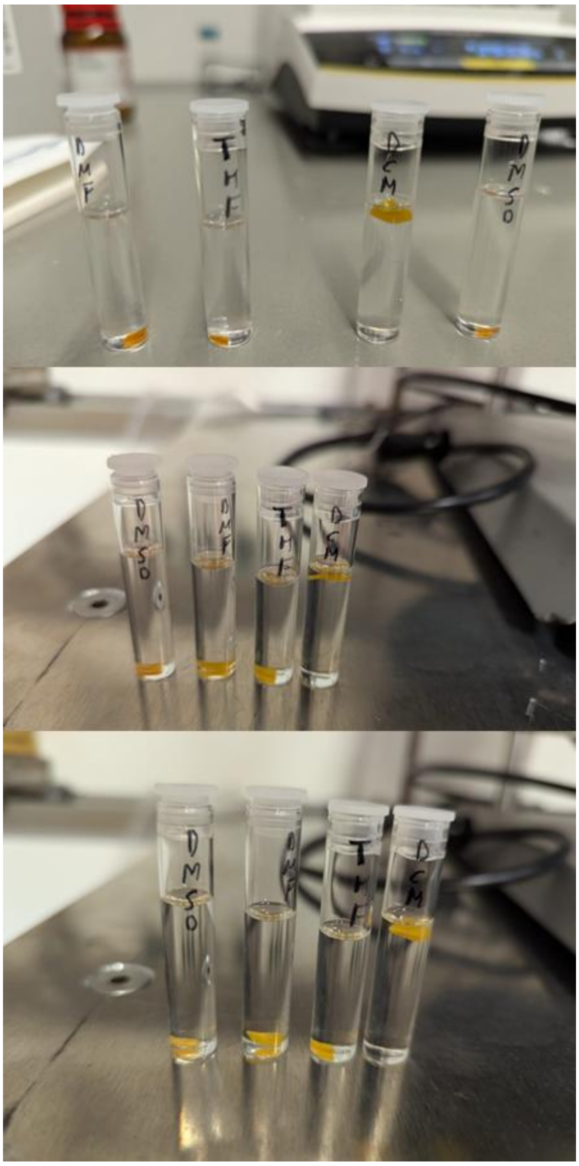

Figure S146: Solubility test on the imine cross-linked material.

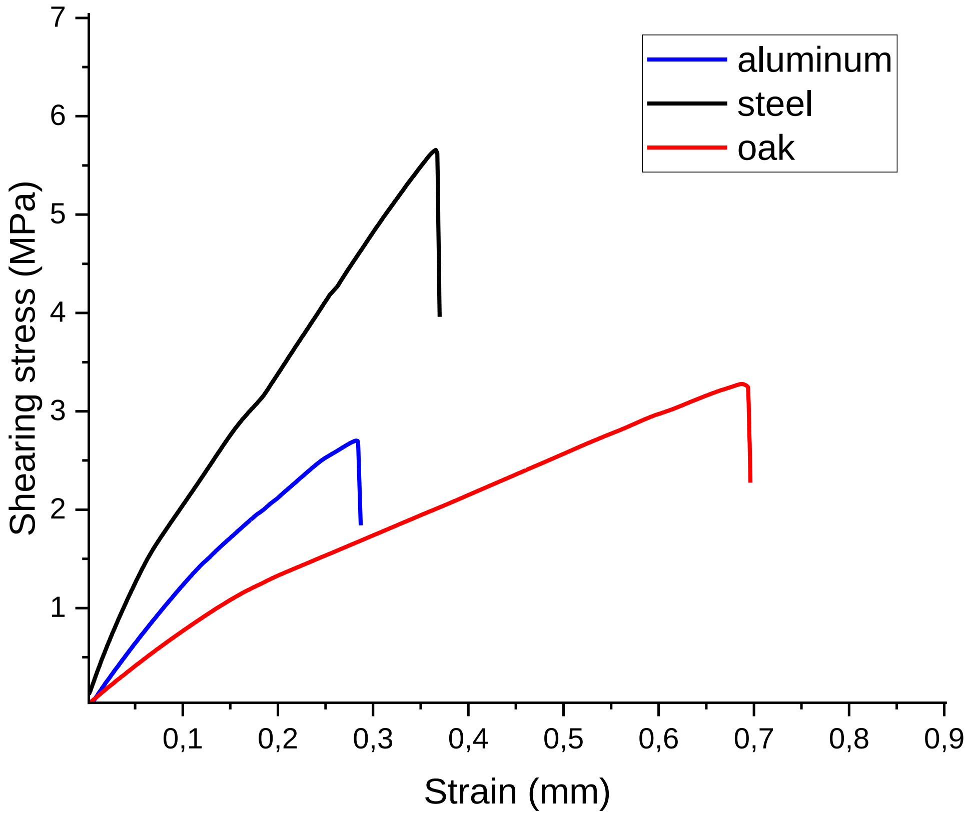

Figure S147: Example lap shear test on different substrates bonded with the imine cross-linked material.

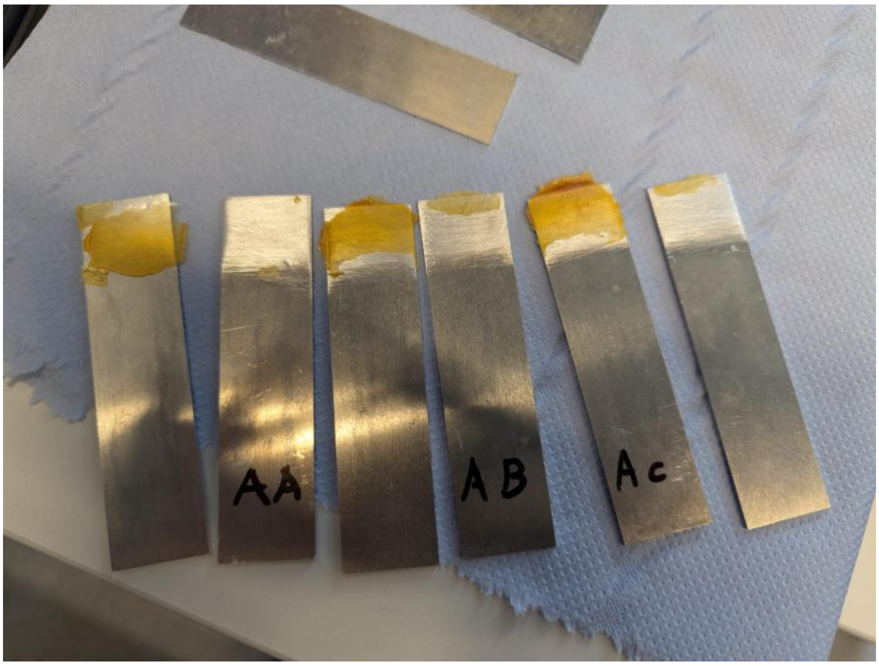

Figure S148: Aluminum plates after lap shear measurement, the almost complete detachment from one of the two plates suggests an adhesive type of failure.

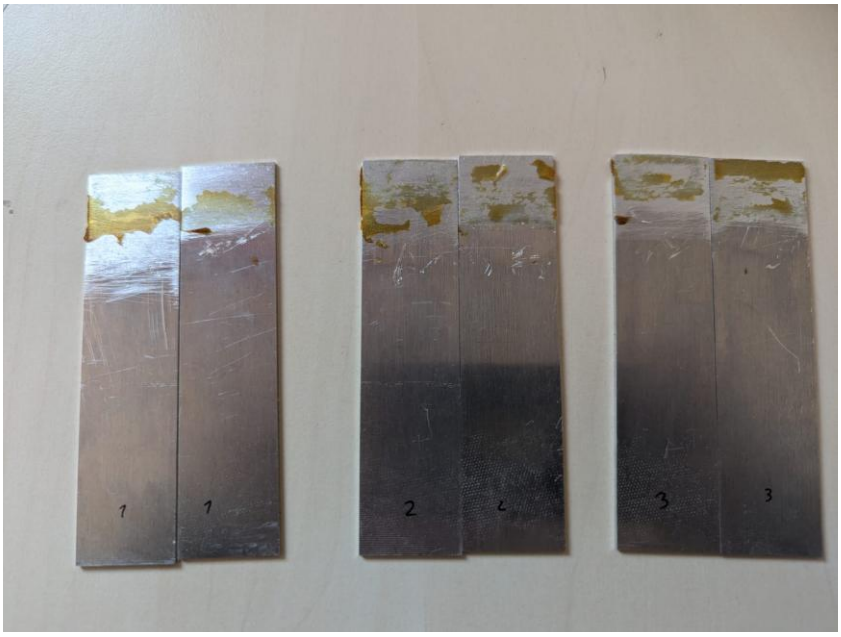

Figure S149: Aluminum plates after lap shear measurement with the lipoic acid functionalized material. The high presence of rupture points within the materials suggests a mostly cohesive failure type.

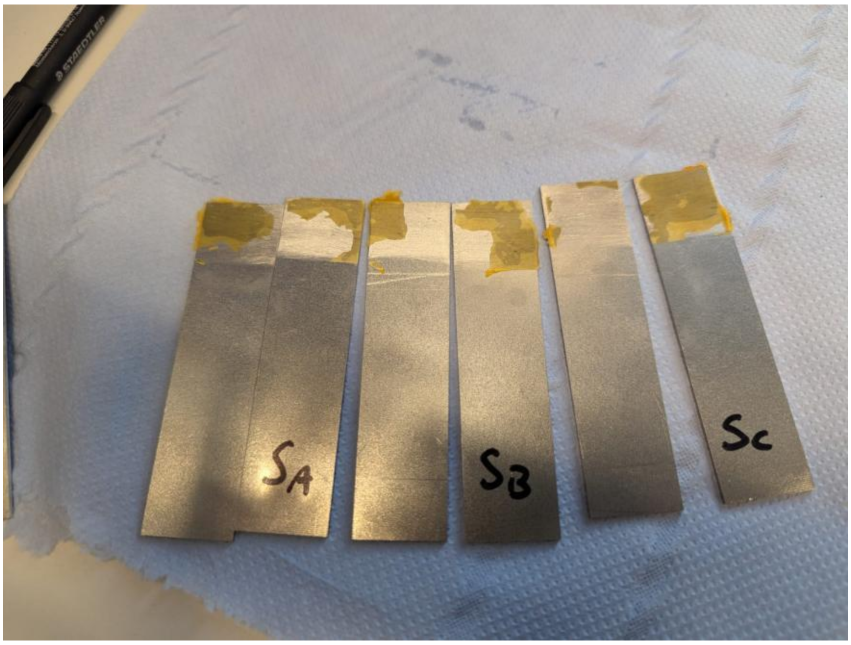

Figure S150: Steel plates after lap shear measurement, the almost complete detachment from one of the two plates suggests an adhesive type of failure but also some degree of cohesive failure can't be excluded.

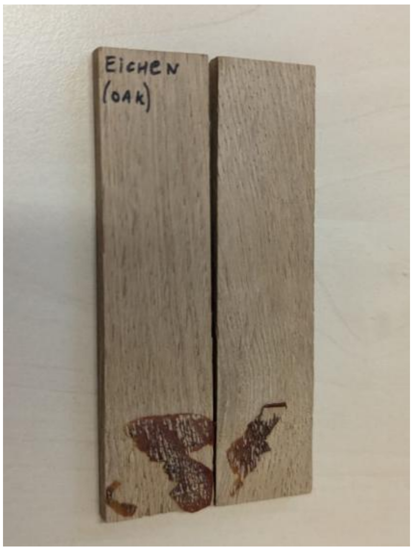

Figure S151: Wood (oak) plate after lap shear measurement, the chunks of wood that remain attached to the polymer suggest a structural type of failure.

	Steel	Steel reprocessed	Reference epoxy glue - steel	Aluminum	Aluminum + lipoic acid	Reference epoxy glue - aluminum
average	5.42	6.58	0.34	2.79	5.55	0.83
St. dev.	1.1829	1.103	0.162	0.774	0.297	0.498

Table S3: Averaged values and standard deviation (three runs) of lap shear tests on different substrates.

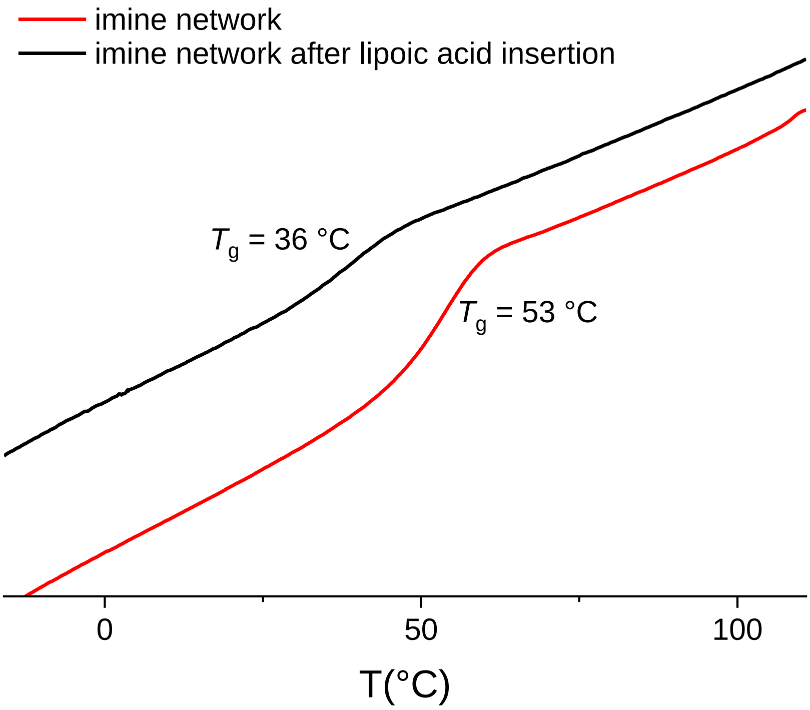

Figure S152: DSC 2nd heating curve of the imine cross-linked polymer before after lipoic acid insertion.

Section S6: DFT calculations

DFT:	M06-2X/def2-TZVPD|| TPSSH/def-TZVP/COSMO($\varepsilon=\infty$)
Solvation:	COSMO-RS (parameters: BP_TZVPD_FINE_C30_1801.ctd)
Solvent:	5:1 mixture of PO and PTA
Temperature:	30°C
Reference state:	x=1 for all species
Dimerization threshold:	-15.0 kJ/mol
“reaction”-call:	-qvib -ct_version 18

Primary Thiolate Addition to PTA (in the Absence of S_8)

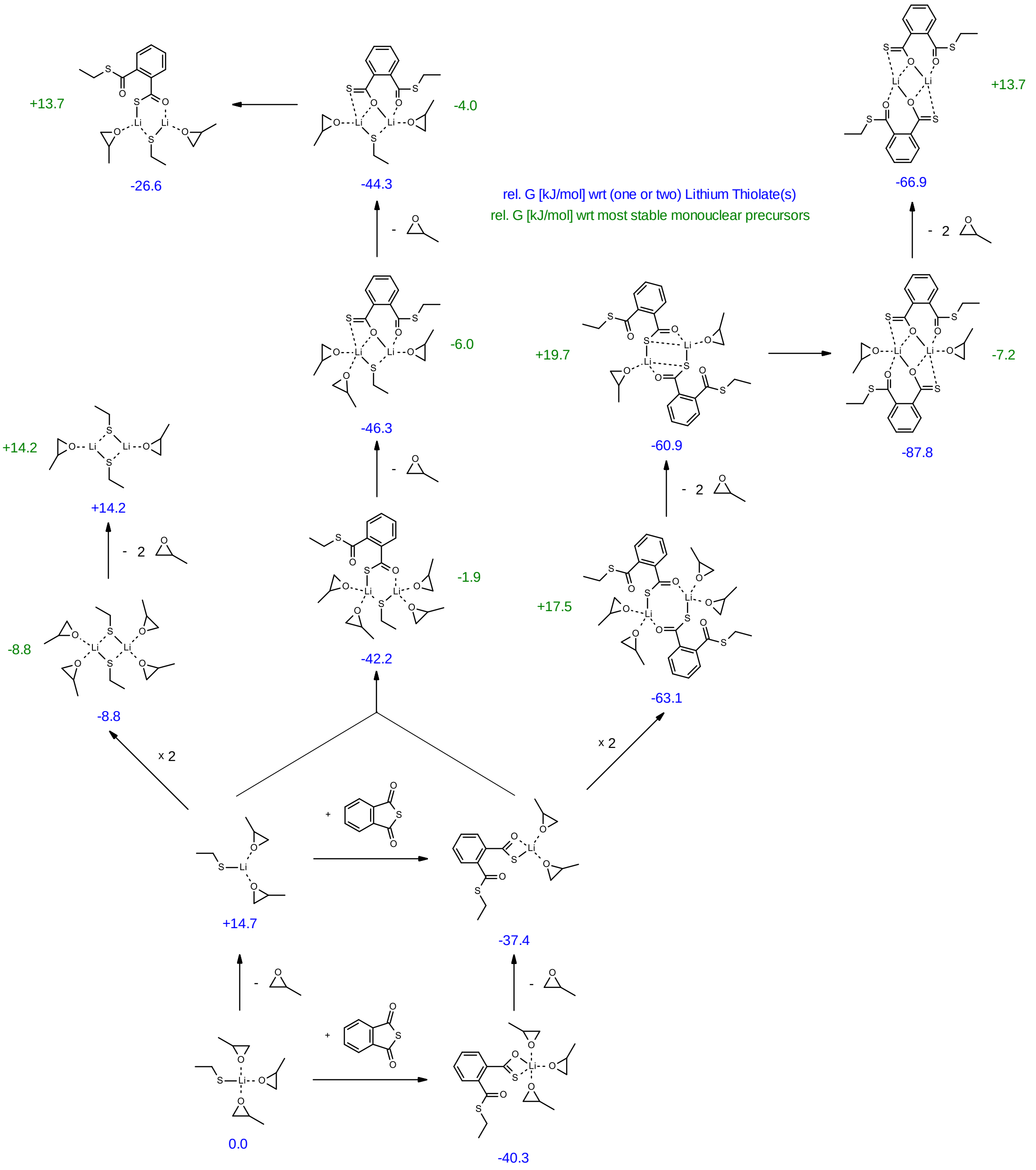

Thiocarboxylate formation by addition of thiolates to PTA (yielding at the same time a thioester linkage) is significantly exergonic. The addition reaction is not expected to exhibit huge activation barriers, PTA being an activated carbonyl compound. Thus, the resting state of the catalytic cycle in copolymerization of PO and PTA is expected to be the thiocarboxylate. With an only weakly exergonic dimerization, the resting state would be a dinuclear complex at the hypothetic reference state of x=1 but is the mononuclear complex at all realistic catalyst concentrations (x~0.001).

Secondary Alkoxide Addition to PTA (in the Presence of S_8) – Assuming a Thiocarboxylate Resting State

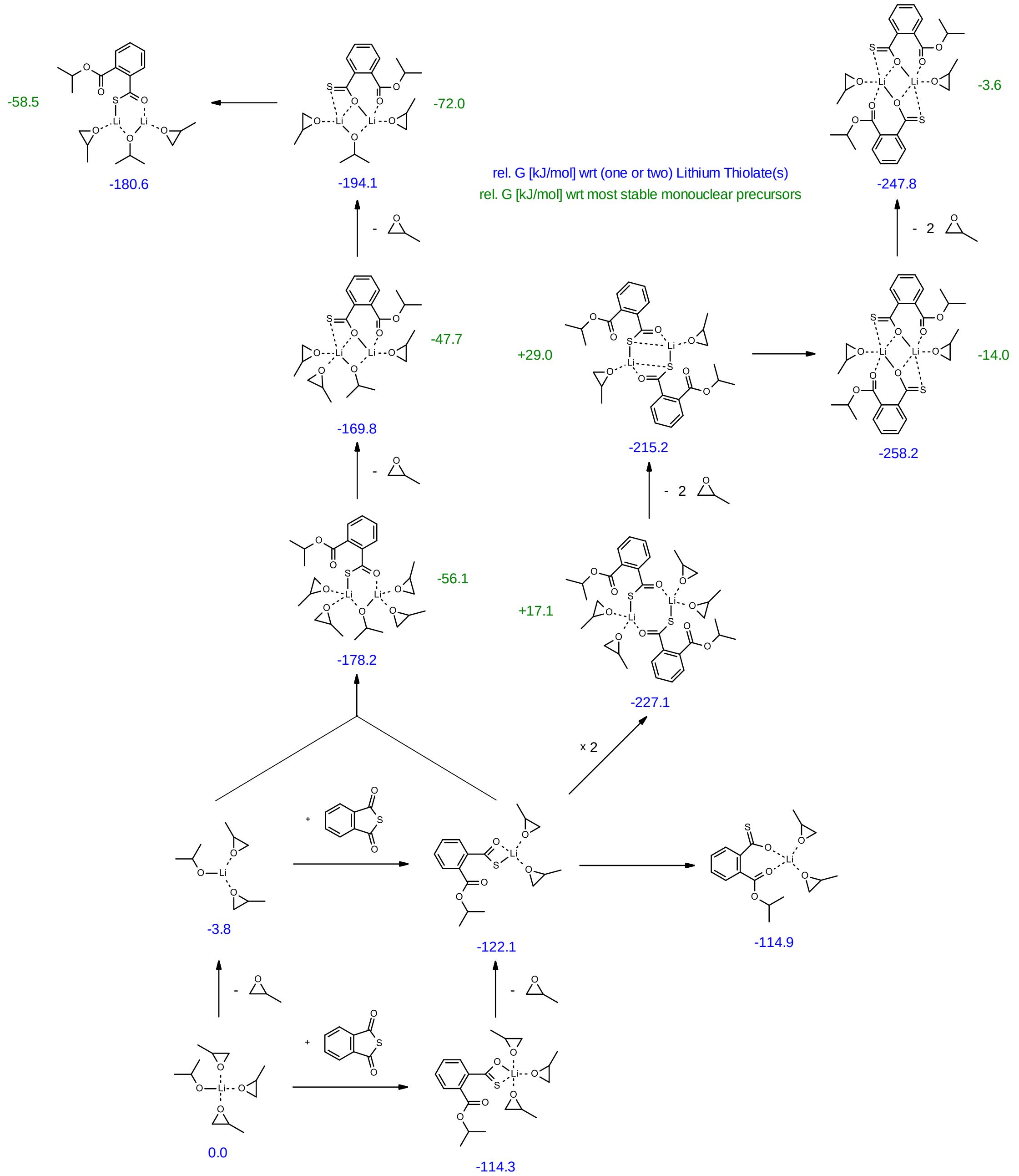

Thiocarboxylate formation by addition of alkoxides to PTA (yielding at the same time an ester linkage) is highly exergonic. The ester linkage is preferred by around 85 kJ/mol over the thioester linkage. Dimerization is also here not strongly exergonic (but more exergonic than in the above case), the resting state would be a dinuclear complex at the hypothetic reference state of x=1 and will be most probably a mixture of mono- and dinuclear complexes at realistic catalyst concentrations (at 30°C, a ΔG of -16.9 kJ would correspond to an equilibrium constant K=1000). Thus, also here for the sake of simplicity mononuclear resting states were assumed.

Trithiolate Speciation

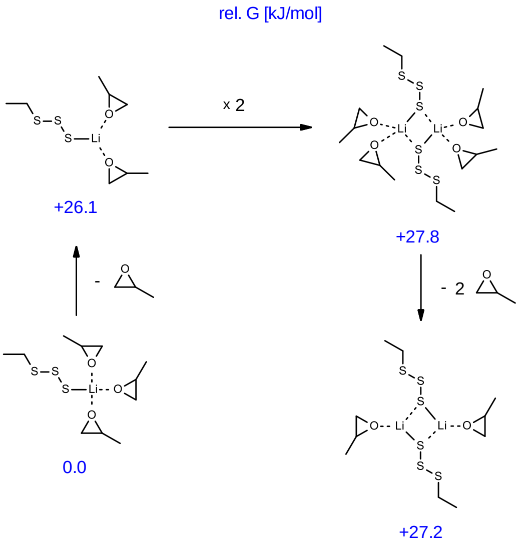

At the trithiolate stage, a mononuclear species with three PO ligands is clearly preferred.

Secondary Alkoxide Addition to PTA (in the Presence of S_8) – Assuming a Trithiolate Resting State

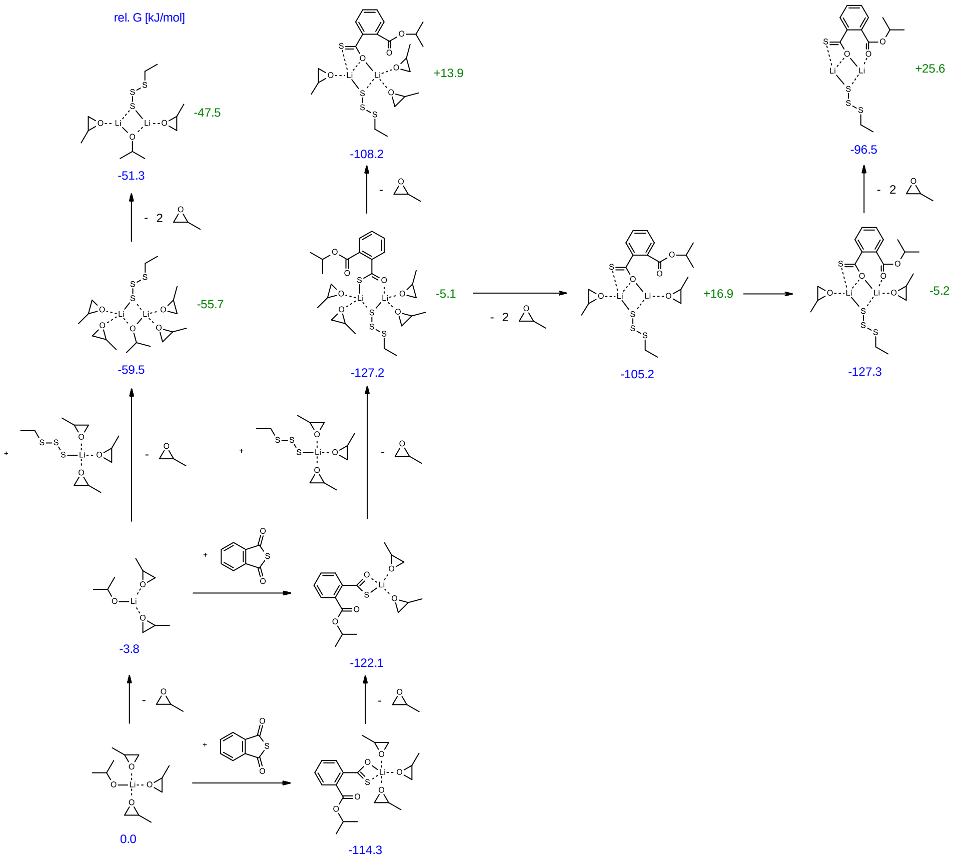

In case of trithiolate as counter ion to form dimeric species, there is a higher preference for mononuclear species than in case of thiocarboxylates, which can be explained by the weaker electron donating ability of trithiolate. However, the general trends are similar as above.

PO Ring Opening by Thiocarboxylates Starting from Potential Resting States

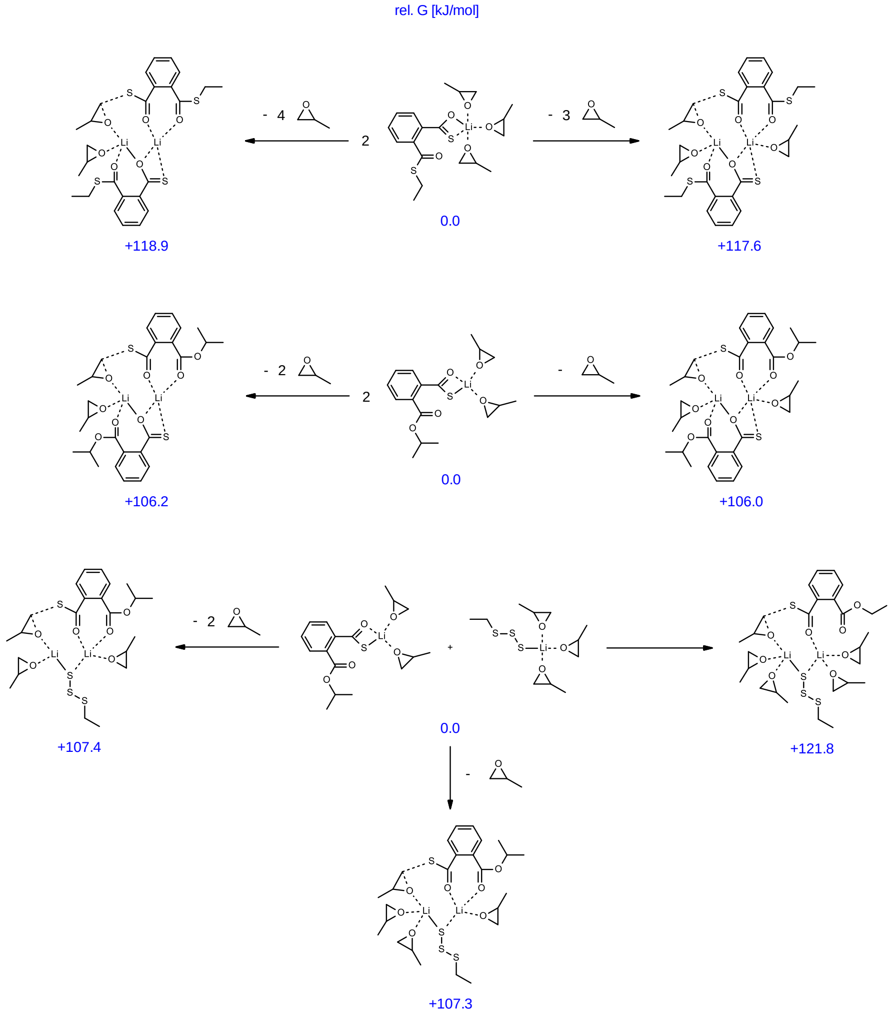

In case of thiocarboxylates with an ortho-ester group, the lowest barrier for PO ring opening is 106.0 kJ/mol. The barrier in case of thiocarboxylates with an ortho-thioester group is with 117.6 kJ/mol significantly higher.

PO Ring Opening by Trithiolates Starting from Potential Resting States

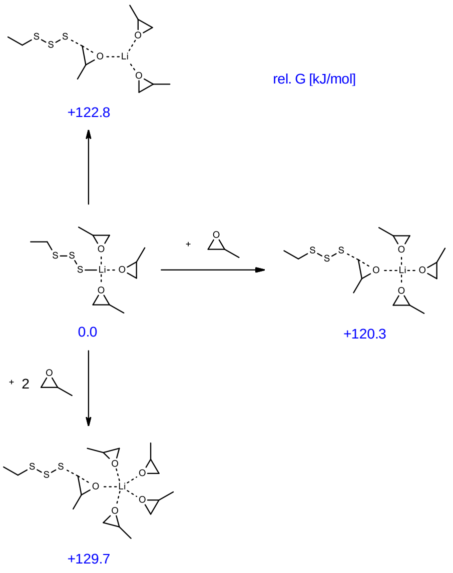

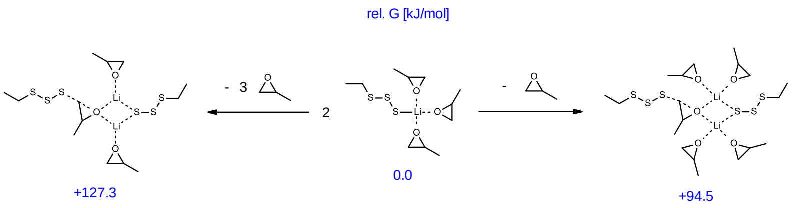

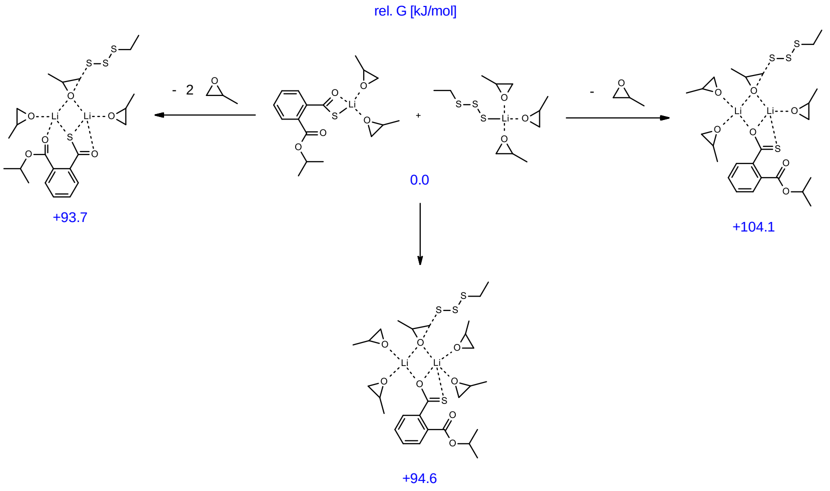

The lowest barriers for PO ring opening are obtained – counter ion independent – via zwitterionic transition states and amount to less than 95 kJ/mol. Thus, PO ring opening by trithiolate is faster than by thiocarboxylates, which leads to the conclusion that the systems exhibits a thiocarboxylate resting state.

Catalytic Cycle

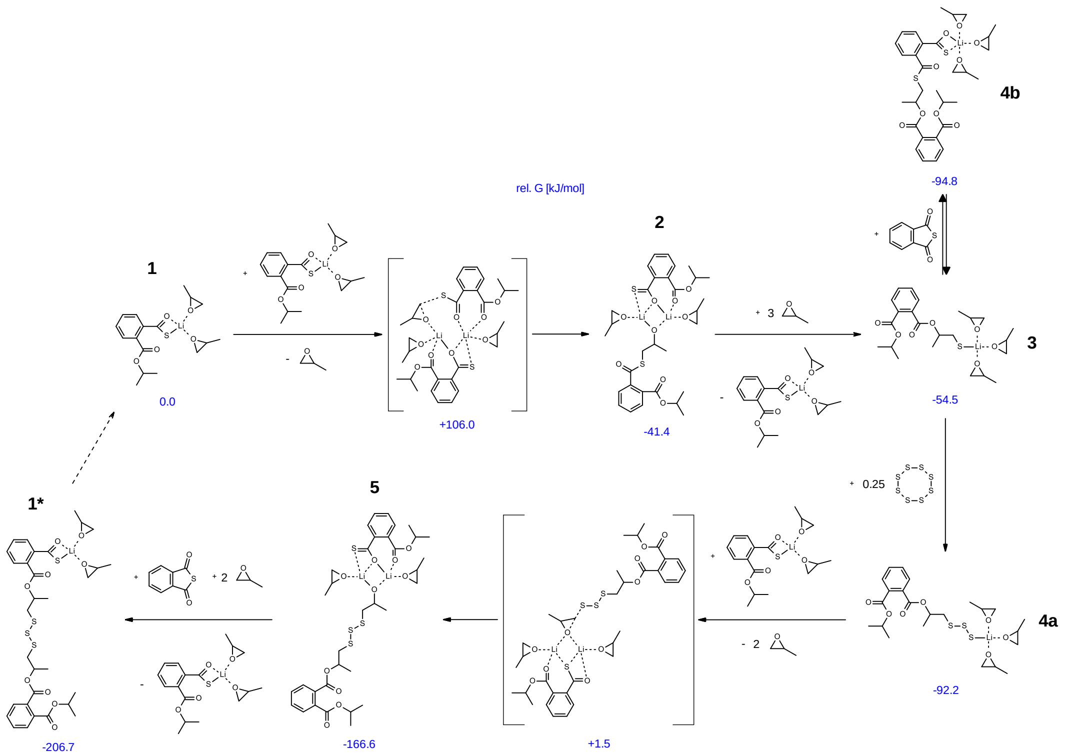

From the resting state **1**, the turnover limiting PO ring opening via a dinuclear transition state yields the preferentially dimeric alkoxide **2**. From previous studies it has been shown, that this species can easily undergo an S/O exchange at the anionic chain end via intermediary cyclization with barriers much lower than for any PO ring opening reaction. In combination with the fact that this rearrangement is exergonic, this means that further reactivity options have to be considered starting from thiolate **3**. One option is addition to PTA, another is reaction with elemental sulfur. From previous studies, both reactions are known to exhibit rather low activation barriers. For the example of a trithiolate product (as an example for several other oligosulfide species), both reactions to **4b** and **4a** are comparably exergonic. The experimental observation that a from thioester chain end (obtained by reaction of a thiolate with PTA) a release of PTA in the presence of sulfur is possible should therefore be a consequence of (a) the respective concentrations and (b) the fact that there is not only the trithiolate but other oligosulfide species in an equilibrium which also means a statistic preference. For the polymer sequence, **4b** represents a dead end, anyway, as the oligosulfides available in the equilibrium with it undergo subsequent PO ring opening much more efficiently. The latter reaction takes place via a zwitterionic mechanism, as the oligosulfides coordinate less strongly to Li^+ and leads to **5**, which is predicted to be a dimer like **2**. Unlike **2**, cannot undergo any rearrangement, thus the alkoxide can practically only perform an addition to PTA, which then concludes the catalytic cycle (**1***); also this step has already been studied before by us and should proceed easily.

Ligand Comparison

NBO population analysis at the M06-2X/def2-TZVPD level. All species are monoanionic.

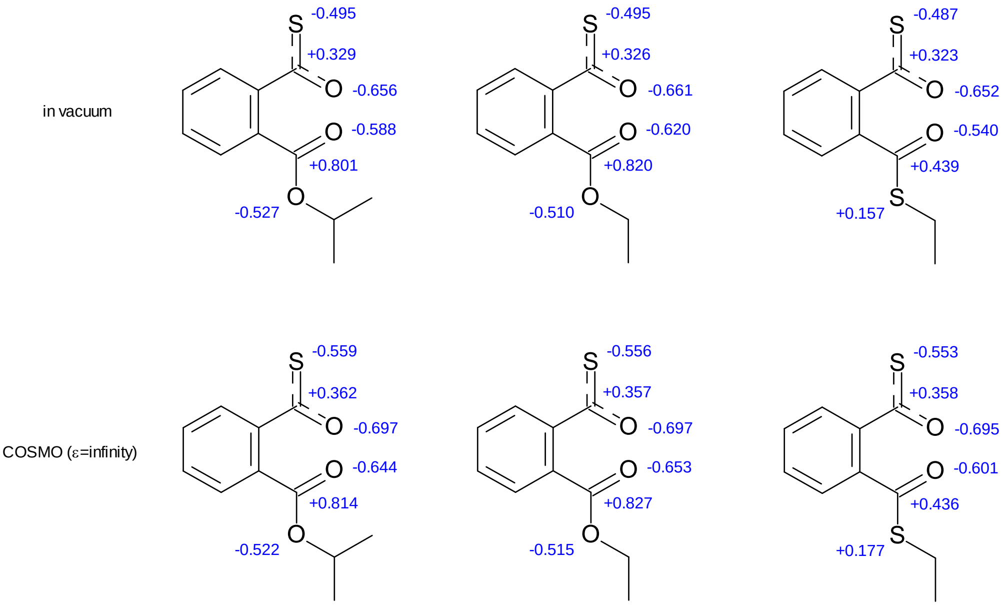

Whereas the nature of the alkyl group does not have a big influence on the partial charge at any potential donor atom, ester and thioester differ significantly: The donating ability of the carbonyl O is much higher in case of the ester than in case of the thioester.

Section S7: References

[1] M. J. Kates,J. H. Schauble, Synthesis of small-medium ring thioanhydrides, Journal of Heterocyclic Chemistry, 1995, 32: 971-978

[2] A. Roig, A. Petrauskaité, X. Ramis, S. De la Flor, À. Serra, Synthesis and characterization of new bio-based poly(acylhydrazone) vanillin vitrimer, Polym. Chem., 2022,13, 1510-1519

[3] Y. Zhang, V. K. R. Tangadanchu, Y. Cheng, R.-G. Yang, J.-M. Lin, C.-H. Zhou, Potential Antimicrobial Isopropanol-Conjugated Carbazole Azoles as Dual Targeting Inhibitors of Enterococcus faecalis, ACS Med. Chem. Lett. 2018, 9, 3, 244–249

[4] Cava, M. P., & Lakshmikantham, M. V. (Aspects of Thioanhydhide Chemistry. Phosphorus, Sulfur, and Silicon and the Related Elements, 1989. 43(1–2), 95–109. https://doi.org/10.1080/10426508908040280

[5] George L. Ellman, Tissue sulfhydryl groups, Archives of Biochemistry and Biophysics, 82, 70-77, 1959.

[6] R. Schmidt, M. G. Logan, S. Patty, J. L. Ferracane, C. S. Pfeifer, and A. J. Kendall, Thiol quantification using colorimetric thiol-disulfide exchange in nonaqueous solvents, ACS Omega, 10, 9356-9363, 2023.
